# Monitoring SARS-CoV-2 Circulation and Diversity through Community Wastewater Sequencing, the Netherlands and Belgium

**DOI:** 10.3201/eid2705.204410

**Published:** 2021-05

**Authors:** Ray Izquierdo-Lara, Goffe Elsinga, Leo Heijnen, Bas B. Oude Munnink, Claudia M.E. Schapendonk, David Nieuwenhuijse, Matthijs Kon, Lu Lu, Frank M. Aarestrup, Samantha Lycett, Gertjan Medema, Marion P.G. Koopmans, Miranda de Graaf

**Affiliations:** Erasmus University Medical Center, Rotterdam, the Netherlands (R. Izquierdo-Lara, B.B. Oude Munnink, C.M.E. Schapendonk, D. Nieuwenhuijse, M. Kon, M.P.G. Koopmans, M. de Graaf);; KWR Water Research Institute, Nieuwegein, the Netherlands (G. Elsinga, L. Heijnen, G. Medema);; University of Edinburgh, Edinburgh, Scotland, UK (L. Lu, S. Lycett);; Technical University of Denmark, Kongens Lyngby, Denmark (F.M. Aarestrup)

**Keywords:** sewage, wastewater, epidemiology, public health, CoV, SARS-CoV-2, Nanopore, Illumina, severe acute respiratory syndrome coronavirus 2, viruses, zoonoses, COVID-19, coronavirus disease, respiratory infections, Belgium, the Netherlands

## Abstract

Severe acute respiratory syndrome coronavirus 2 (SARS-CoV-2) has rapidly become a major global health problem, and public health surveillance is crucial to monitor and prevent virus spread. Wastewater-based epidemiology has been proposed as an addition to disease-based surveillance because virus is shed in the feces of ≈40% of infected persons. We used next-generation sequencing of sewage samples to evaluate the diversity of SARS-CoV-2 at the community level in the Netherlands and Belgium. Phylogenetic analysis revealed the presence of the most prevalent clades (19A, 20A, and 20B) and clustering of sewage samples with clinical samples from the same region. We distinguished multiple clades within a single sewage sample by using low-frequency variant analysis. In addition, several novel mutations in the SARS-CoV-2 genome were detected. Our results illustrate how wastewater can be used to investigate the diversity of SARS-CoV-2 viruses circulating in a community and identify new outbreaks.

Since its discovery, severe acute respiratory syndrome coronavirus 2 (SARS-CoV-2) has caused >100 million confirmed cases of coronavirus disease (COVID-19). The global effects of SARS-CoV-2 and the need to learn more about its origin and epidemiology have resulted in the sequencing of >416,000 genomes as of January 2021 (*1*). This work has enabled the identification of groups of viruses that, on the basis of their genetic diversity, can be associated with geographic and temporal patterns of virus spread (*2*). Nextstrain (https://nextstrain.org) currently divides SARS-CoV-2 diversity into 12 major global clades (19A, 19B, and 20A–20J), on the basis of high prevalence, signature mutations, and geographic spread (*3*).

Although SARS-CoV-2 primarily affects respiratory tract tissues, it can also replicate in the gastrointestinal tract, as evidenced by in vitro infection of enteroids (*4*), presence of viral proteins in gastrointestinal epithelium biopsy specimens (*5*), and detection of infectious virus in stool samples (*6*). Viral RNA is shed in the feces of ≈40% of infected persons, often for longer periods than the virus can be detected in nasal swab specimens. SARS-CoV-2 RNA has been detected in urine occasionally (<5% of infected patients) (*7*–*9*).

Because of the rapid spread of SARS-CoV-2, individual screening of clinical cases and study of viral diversity on a population level are challenging. Various reports have demonstrated that enteric and respiratory viruses can be detected in wastewater (*10*–*18*). This finding has led to the recognition of wastewater-based epidemiology as a potentially valuable tool to assess the spread of the disease at a community level. Recently, the Water Research Institute in the Netherlands and other groups have demonstrated temporal correlations between SARS-CoV-2 RNA titers in sewage and the number of reported cases in a city or county when >26 gene copies per liter could be detected (*14*,*19*–*21*). Therefore, sewage testing is currently considered globally to be an adjunct to patient-based surveillance and demonstrates promise as an early warning indicator of increasing virus circulation.

Enhanced surveillance is a key pillar of the current strategy to control the spread of SARS-CoV-2 and includes frequently testing mildly symptomatic persons, investigating infection clusters to identify possible common exposures, and monitoring hospital admission rates. Whole-genome sequencing of SARS-CoV-2 from clinical samples has been adopted as an additional tool to identify clusters. Particularly in geographic areas with minimal virus circulation, sequencing can help identify possible sources, provided that sufficient background sequencing has been performed. So far, little work has been done to correlate SARS-CoV-2 diversity in sewage samples with diversity in patients (*22*,*23*). We used next-generation sequencing (NGS) of SARS-CoV-2 from wastewater samples to assess whether these samples reflect the diversity of SARS-CoV-2 circulating within the population of the Netherlands and Belgium.

## Methods

### Sample Preparation

Wastewater specimens were collected as 24-h flow-dependent composite samples and processed as previously described (*14*). Debris of 100–200 mL of sewage samples was pelleted and the supernatant was concentrated by using 100 kDa Centricon ultrafilters (Millipore Sigma, https://www.emdmillipore.com); in vitro–transcribed dengue virus type-2 RNA was added as an internal extraction control. RNA was extracted by using the Nuclisens kit (bioMérieux, https://www.biomerieux.com) and KingFisher purification system (Thermo Fisher Scientific, https://www.thermofisher.com) (*14*). RNA was screened by quantitative reverse transcription PCR (qRT-PCR) with 5 primer–probe sets targeting the SARS-CoV-2 nucleocapsid (N) gene (N1–N3) (*24*), envelope (E) gene for all sarbecoviruses (*25*), and the internal control.

### NGS

We performed SARS-CoV-2–specific multiplex PCR for nanopore sequencing as described previously (*26*). Primers for 89 overlapping amplicons spanning the genome were used in 2 PCR pools. Libraries were generated by using the Oxford Nanopore native barcode kits (Oxford Nanopore Technologies, https://nanoporetech.com) and sequenced on a R9.4 flow cell.

Illumina sequencing was performed as described previously (*27*). Amplicons were generated by the multiplex PCR described previously. Amplicons were purified with 0.8X AMPure XP beads (Beckman Coulter, https://www.beckmancoulter.com) and 100 ng of DNA was converted into paired-end Illumina sequencing libraries by using the KAPA HyperPlus library preparation kit (Roche, https://www.roche.com). We used the KAPA Unique Dual-Indexed Adapters Kit (Roche) to enable subsequent sequencing of multiple libraries in a single Illumina MiSeq version 3 flowcell (2 × 300 cycles) (Illumina, https://www.illumina.com).

### Nanopore Sequence Analysis

Raw sequence data were processed as previously described (*26*). We used a snakemake script to demultiplex fastq raw reads by using Porechop (https://github.com/rrwick/Porechop), to trim primers by using Cutadapt (*28*), and to perform a reference-based alignment by using minimap2 to GISAID sequence EPI_ISL_412973 (https://www.gisaid.org). The run was monitored by using RAMPART (https://artic-network.github.io/rampart). The consensus genome was extracted by using 2 analyses for which positions with a coverage <10X or <30X were replaced with an N. We confirmed mutations in the genome by manually checking the alignment in Ugene (*29*) and resolved homopolymeric regions by consulting reference genomes. On the basis of previous studies (*30*), we considered mutations with >30X coverage high quality, whereas mutations >10X and <30X coverage were considered low quality.

### Illumina Sequence Analysis

We used a customized Galaxy workflow (*31*) for all processing, reference-based alignment, and variant analysis. Raw sequencing reads were filtered by using Fastp (*32*) to remove adaptor contamination, ambiguous bases, low quality reads (Phred score <30), and fragments <50 nt. Reads were mapped against GISAID sequence EPI_ISL_412973 by using the default settings of BWA-MEM (H. Li, unpub. data, https://arxiv.org/abs/1303.3997). Reads were realigned by using the leftalign utility from FreeBayes (E. Garrison, unpub. data, https://arxiv.org/abs/1207.3907). All reads with mapping scores of <30 were discarded. Consensus sequences and variants were generated by using iVar (*33*). Final consensus sequences (frequency >50%) were constructed by using all mapped reads with a coverage of >5X and Phred score of >30. For detection of low-frequency variants (LFVs), we used parameters as follows: minimum coverage of 50X, Phred score >30, and a minimum frequency threshold of 10%. Variant calling was confirmed by manual inspection of the aligned reads in Ugene (*29*). Variant positions are given with respect to the Wuhan-Hu-1 strain (MN908947) (*34*). We uploaded all consensus sequences with coverage >50% to GISAID (accession nos. EPI_ISL_539300–25).

### Phylogenetic Analysis

The first dataset included all full-length SARS-CoV-2 genomes from the Netherlands (1,544 genomes) and Belgium (888 genomes) from GISAID as of July 8, 2020. The second dataset was a subsample representative of the global diversity of all SARS-CoV-2 sequences in GISAID as of June 1, 2020. This global dataset contained 2,552 subsampled sequences (full length with Ns <5%) to include 1 unique genome per country or state per week. We aligned sequences with >75% genome coverage by using MAFFT (https://mafft.cbrc.jp/alignment/server) and inferred maximum-likelihood trees by using the best predicted models general time-reversible plus F plus R3 (global subsample) and general time-reversible plus F plus R2 (Netherlands–Belgium dataset) and bootstrap with 1,000 replicates. Trees were visualized by using Figtree version 1.4.4 (http://tree.bio.ed.ac.uk/software/figtree). Clades were assigned by using the Nextclade tool.

## Results

### Correlation between qRT-PCR and Percentage of Genome Recovered

Previously, sewage samples collected from 6 locations in the Netherlands (and Schiphol Airport) were tested by qRT-PCR to investigate the levels of SARS-CoV-2 RNA (*14*). To further investigate the genetic diversity of SARS-CoV-2, we subjected 55 wastewater samples obtained from 13 locations in the Netherlands (48 samples) and 7 locations in Belgium (7 samples) with cycle threshold (C_t_) values of <36 to whole-genome sequencing by using nanopore technology. The wastewater treatment plants in the Netherlands served ≈200,000–980,000 inhabitants; Schiphol was estimated to serve 54,000 persons (*14*). The samples covered a period of 70 days (March 25–June 3, 2020); of all 55 samples, 2 (Franeker-92719 and AmsterdamWest-92852) were sequenced by nanopore twice. Of the 55 samples, 24 were also sequenced by Illumina (Table 1).

**Table 1:** Overview of SARS-CoV-2 wastewater samples sequenced during study of circulation and diversity through community wastewater sequencing, the Netherlands and Belgium*

Sample no.	Sample ID	Date	Country	Sampling location	Target C_t_†					Coverage, %	
N1	N2	N3	E	Nanopore	Illumina
1	92499	2020 Mar 25	Netherlands	Heeswijk-Dinther	32.9	32.1	30.7	30.8		94.4	ND
2	92502	2020 Mar 25	Netherlands	Apeldoorn	36.6	34.9	33.2	33.3		74.9	19.4
3	92503	2020 Mar 25	Netherlands	Amersfoort	34.9	33.1	31.8	32.1		87.8	ND
4	92504	2020 Mar 25	Netherlands	Utrecht	31.8	30.9	29.8	29.9		95.2	ND
5	92505	2020 Mar 25	Netherlands	Utrecht Overvecht	32.3	31.1	30.1	30.1		92.4	ND
6	92506	2020 Mar 25	Netherlands	Schiphol	32.7	32.0	30.8	30.7		92.2	65.6
7	92508	2020 Mar 25	Netherlands	Amsterdam West	31.8	30.7	29.7	29.9		97.0	ND
8	92509	2020 Mar 25	Netherlands	Tilburg	33.0	32.2	31.2	31.0		78.8	65.5
9	92719	2020 Mar 30	Netherlands	Franeker	31.8	30.8	31.2	30.7		97.7/50.9‡	78.2
10	92721	2020 Mar 30	Netherlands	Beverwijk	32.6	31.4	31.8	30.8		93.7	47.7
11	92722	2020 Mar 30	Netherlands	Katwoude	32.9	32.6	32.7	31.4		84.6	53.9
12	92723	2020 Mar 30	Netherlands	Wervershoof	33.1	32.3	32.5	31.1		96.6	43.2
13	92848	2020 Apr 1	Netherlands	Amersfoort	33.6	32.1	32.3	31.6		96.6	39.4
14	92849	2020 Apr 1	Netherlands	Utrecht	32.4	31.4	31.8	30.6		57.8	48.5
15	92851	2020 Apr 1	Netherlands	Schiphol	33.7	33.1	33.4	32.3		89.4	53.5
16	92852	2020 Apr 1	Netherlands	Amsterdam West	31.8	30.6	30.9	29.9		99.2/97.1‡	59.1
17	92853	2020 Apr 1	Netherlands	Tilburg	33.5	32.6	32.6	32.0		91.2	ND
18	92943	2020 Apr 2	Belgium	Langemark	33.2	33.3	33.1	32.2		60.3	ND
19	92947	2020 Apr 2	Belgium	Lo-Reninge	34.6	34.2	34.5	33.4		71.3	ND
20	92949	2020 Apr 2	Belgium	Properinge	34.5	33.4	33.4	32.4		65.6	65.6
21	92965	2020 Apr 2	Netherlands	Delft	32.9	32.9	32.9	31.5		91.7	52.4
22	93030	2020 Apr 5	Belgium	Aartselaar	33.2	32.4	31.6	31.4		89.9	61.2
23	93032	2020 Apr 5	Belgium	Gent	34.2	33.7	32.6	32.1		63.2	46.9
24	93034	2020 Apr 5	Belgium	Leuven	33.6	33.4	32.1	31.4		70.2	37.6
25	93036	2020 Apr 5	Belgium	Tienen	33.3	32.6	31.2	30.8		88.1	41.5
26	93818	2020 Apr 8	Netherlands	Amersfoort	34.9	34.3	33.4	32.4		37.5	ND
27	93820	2020 Apr 9	Netherlands	Utrecht	32.8	32.2	31.2	30.8		55.2	ND
28	93822	2020 Apr 9	Netherlands	Amsterdam West	32.6	25.1	31.6	30.9		87.3	ND
29	93823	2020 Apr 9	Netherlands	Schiphol	33.0	33.2	32.2	31.3		67.3	43.5
30	93825	2020 Apr 8	Netherlands	Delft	33.9	33.7	32.7	32.0		63.9	64.3
31	93828	2020 Apr 9	Netherlands	Tilburg	35.2	34.6	33.1	32.7		31.2	ND
32	93948	2020 Apr 14	Netherlands	Heeswijk-Dinther	35.8	34.6	33.6	32.7		18.8	ND
33	93950	2020 Apr 15	Netherlands	Wervershoof	34.9	34.3	33.1	32.5		60.7	ND
34	94330	2020 Apr 21	Netherlands	Utrecht1	35.1	34.4	33.2	33.5		41.7	ND
35	94331	2020 Apr 21	Netherlands	Utrecht2	35.7	34.1	34.2	33.7		38.6	ND
36	94334	2020 Apr 21	Netherlands	Amsterdam West	34.0	33.3	32.4	32.0		66.7	ND
37	94335	2020 Apr 21	Netherlands	Schiphol	33.8	34.1	32.9	33.7		40.1	43.0
38	94337	2020 Apr 21	Netherlands	Delft	35.7	34.1	34.1	33.8		34.2	ND
39	94339	2020 Apr 21	Netherlands	Tilburg	34.8	35.4	34.7	36.0		11.2	80.3
40	94602	2020 Apr 29	Netherlands	Utrecht	35.6	34.3	33.0	34.2		29.6	ND
41	94604	2020 Apr 29	Netherlands	Amsterdam West	34.9	34.6	32.8	33.6		15.0	ND
42	94605	2020 Apr 29	Netherlands	Schiphol	34.6	35.1	33.6	33.2		21.3	35.2
43	94607	2020 Apr 25	Netherlands	Delft	35.8	36.2	34.4	34.0		15.3	ND
44	94976	2020 May 7	Netherlands	Utrecht	35.5	36.0	35.1	33.5		6.3	ND
45	94978	2020 May 7	Netherlands	Amsterdam West	35.0	34.8	34.5	33.7		19.8	ND
46	94982	2020 May 6	Netherlands	Delft	35.1	35.9	34.7	33.7		18.7	ND
47	95550	2020 May 13	Netherlands	Utrecht	ND	34.4	ND	32.0		3.0	ND
48	95552	2020 May 13	Netherlands	Amsterdam West	ND	34.2	ND	32.8		20.4	ND
49	95556	2020 May 12	Netherlands	Delft	ND	34.4	ND	34.1		0	ND
50	95558	2020 May 13	Netherlands	Tilburg	ND	34.3	ND	36.1		0	ND
51	95793	2020 May 19	Netherlands	Utrecht	ND	35.1	ND	34.9		0	ND
52	95794	2020 May 19	Netherlands	Amsterdam West	ND	35.1	ND	34.2		7.7	ND
53	96925	2020 Jun 2	Netherlands	Utrecht	ND	35.2	ND	37.1		0	ND
54	96927	2020 Jun 2	Netherlands	Schiphol	ND	32.5	ND	31.1		30.8	34.0
55	97044	2020 Jun 3	Netherlands	Delft	ND	35.7	ND	33.5		8.2	ND

*C_t_, cycle threshold; E, envelope; N, nucleocapsid; ND, not determined; SARS-CoV-2, severe acute respiratory syndrome coronavirus 2. †Three primer–probe sets targeting the N1–N3 genes and 1 targeting the E gene. ‡These samples were sequenced twice.

**Figure 1:**
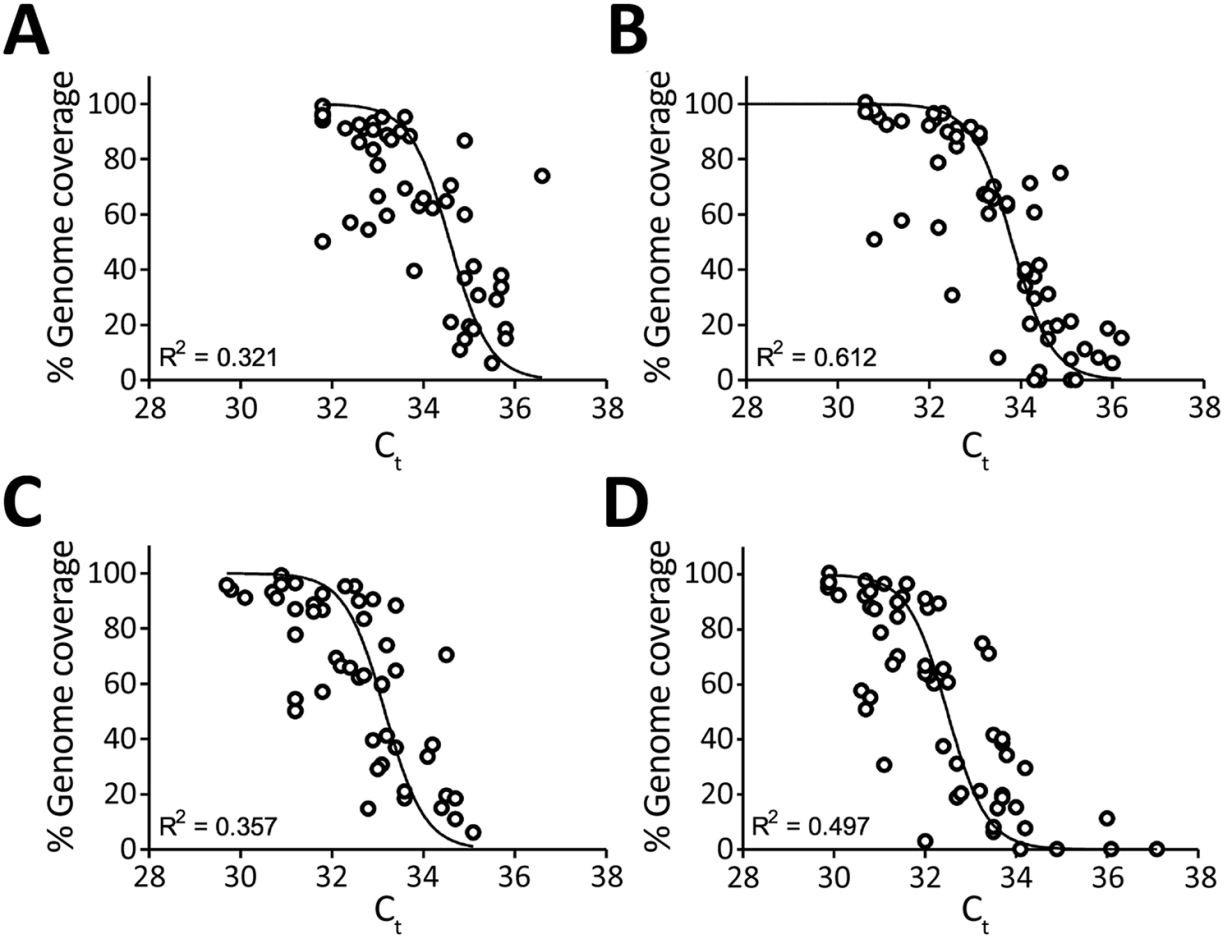
Quantitative reverse transcription PCR C_t_ of severe acute respiratory syndrome coronavirus 2 RNA in sewage samples as determined by N gene (N1–N3) and E gene assays against the percentage of genome covered (>10×) by nanopore reads, the Netherlands and Belgium. A) N1 gene; B) N2 gene; C) N3 gene; D) E gene. C_t_, cycle threshold.

We used 4 primer–probe sets targeting the N (N1–N3) genes and E gene to evaluate the concentration of SARS-CoV-2 in sewage samples (Table 1) (*14*). The percentage of the genome covered by the assembly of nanopore reads (>10X coverage) ranged from 0% to 99.2%. We found an inverse sigmoidal correlation between the percentage of the genome assembled from nanopore sequencing reads and the N and E gene C_t_ values (Figure 1). The C_t_ values at which half of the genome could be obtained were 34.6 for N1, 33.8 for N2, 33.2 for N3, and 32.5 for E. No correlation was observed between C_t_ values and the percentage of the genome assembled from Illumina reads (Appendix Figure 1).

### Consensus Sequences

**Figure 2:**
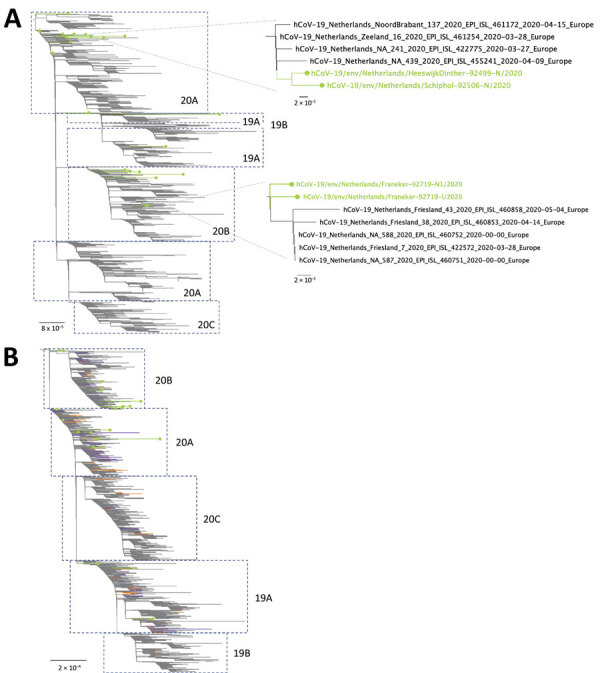
Phylogenetic analysis of severe acute respiratory syndrome coronavirus 2 genome consensus sequences detected in sewage samples, the Netherlands and Belgium. A) The Netherlands subsample dataset; B) global subsample dataset. Lines with dots in green indicate samples sequenced in this study. Clades (19A, 19B, 20A, 20B, and 20C) were assigned by using the Nextclade tool (https://clades.nextstrain.org). For the global subsample tree, samples in orange indicate the Netherlands sequences. Samples in purple indicate Belgium sequences. Scale bars indicate inferred number of nucleotide substitutions per site.

We performed phylogenetic analysis to assess whether consensus sequences from sewage could be associated with clinical samples from the same region. A total of 22 genomes (20 from nanopore and 2 from Illumina runs) with a coverage >75% of the genome were obtained from 20 samples. We used these sequences to infer a maximum-likelihood tree using all sequences from the Netherlands and Belgium available in GISAID and a maximum-likelihood tree using a subset representative of the global diversity of SARS-CoV-2 in GISAID. In general, the sequences from the Netherlands and Belgium grouped into 5 clades (Figure 2, panel A), and most of the sequences belonged to clade 20A (52.0% for the Netherlands and 47.7% for Belgium). The clades 19B and 20C were less prevalent; 8.9% of sequences from the Netherlands belonged to 19B and 1.2% to 20C, whereas 10.4% of Belgium sequences belonged to 19B and 0.3% to 20C. Both trees showed that sewage samples grouped within clades 19A, 20A, and 20B (Figure 2). Samples Franeker-92719 and HeeswijkDinther-92499 clustered with sequences isolated from patients from the same region (Figure 2, panel A), indicating that sewage samples can be linked to specific outbreaks. Included in the phylogenetic trees were 2 samples with 2 consensus sequences (AmsterdamWest-92852 and Franeker-92719), which demonstrated 2-mutation differences between consensus sequences of the same sample (Appendix Table 1). Despite this discrepancy, consensus sequences from the same sample clustered within the same clade (Appendix Figures 2, 3). Some sequences clustered close to the root of the tree, probably because of the presence of multiple strains within 1 sample, which resulted in a combination of mutations in their consensus sequences.

**Figure 3:**
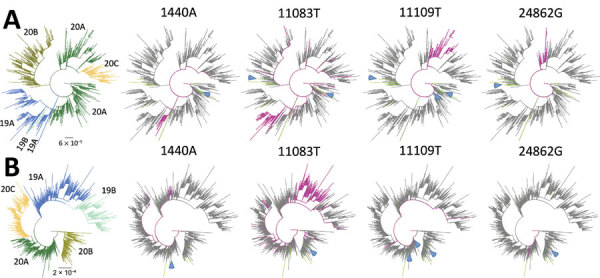
Phylogenetic trees showing 4 low-frequency variants detected in sewage samples in study of severe acute respiratory syndrome coronavirus 2 circulation and diversity through community wastewater sequencing, the Netherlands and Belgium. A) The Netherlands–Belgium subsample; B) global subsample. Patient sequences containing the mutation are shown in magenta. Lines in green indicate sewage samples sequenced in this study. Clades (19A, 19B, 20A, 20B, and 20C) are indicated in colors at the left of the figure. Blue arrows show the consensus sequences (if available) of the sewage samples in which the low-frequency variant was detected. Scale bars indicate the inferred number of nucleotide substitutions per site.

To associate samples with a particular clade or cluster, we compared all consensus sequences, including partial sequences, with the Wuhan-Hu-1 reference isolate. A total of 145 single-nucleotide polymorphisms (SNPs) were detected in our dataset (Appendix Table 1). Of these, 24 SNPs were detected in >1 sequence. We also detected SNPs in the Netherlands sewage sequences with a geographic regional signal, which were present in the Netherlands clinical samples at much higher frequencies than in global or Belgium clinical samples, such as T514C and C1594T (Appendix Table 2).

**Table 2:** Summary of LFVs detected in wastewater samples determined by Illumina sequencing in study of SARS-CoV-2 circulation and diversity through community wastewater sequencing, the Netherlands and Belgium*

Position†	Sample	MV	LFV	LFV, %	Total depth	Feature	AA MV	AA LFV	Frequency, %‡		
									NL	BE	Global
1440	NL/Schiphol-92506-I	G	A	13.2	53	ORF1a	G	N	1.619	4.167	1.903
3549	NL/Franeker-92719-I	GACCA CTTA	–§	46.8	201	ORF1a	GPLK	E	0	0	0
4497	NL/Beverwijk-92721-I	T	C	42.6	479	ORF1a	I	T	0	0	0.000
10514	NL/AmsterdamWest-92852-I	T	C	12.5	1,656	ORF1a	Y	H	0	0	0
10933	BE/Aartselaar-93030-I	C	T	18.0	50	ORF1a	P	P	100.000	100.000	99.996
	NL/Tilburg-94339-I	T	C	11.1	63	ORF1a	P	P	0	0	0.004
11083	BE/Properinge-92949-I	G	T	12.1	58	ORF1a	L	F	5.635	7.320	11.007
	BE/Aartselaar-93030-I	T	G	26.4	129	ORF1a	F	L	94.430	92.680	88.069
	NL/Tilburg-94339-I	G	T	12.7	150	ORF1a	L	F	5.635	7.320	11.007
11109	NL/AmsterdamWest-92852-I	C	T	48.3	230	ORF1a	A	V	15.220	0.338	0.534
	NL/Tilburg-94339-I	C	T	21.2	66	ORF1a	A	V	15.220	0.338	0.534
11484	NL/Beverwijk-92721-I	C	T	44.0	84	ORF1a	A	V	0	0	0
11494	NL/Franeker-92719-I	C	T	13.5	104	ORF1a	N	N	0	0	0.002
	BEAartselaar-93030-I	C	T	43.5	370	ORF1a	N	N	0	0	0.002
	NL/Tilburg-94339-I	C	T	13.8	247	ORF1a	N	N	0	0	0.002
13046	BE/Aartselaar-93030-I	C	A	36.7	98	ORF1a	P	T	0	0	0
13426	BE/Gent-93032-I	C	T	22.6	115	ORF1a	R	R	0	0	0.038
16538	BE/Gent-93032-I	–	ATA	27.6	348	ORF1b	–	N	100.000	100.000	100.000
16777	NL/Schiphol-92851-I	G	T	30.2	404	ORF1b	V	F	0	0	0
16806	NL/Tilburg-94339-I	C	A	22.1	77	ORF1b	N	K	0	0	0.016
16823	BE/Aartselaar-93030-I	G	T	12.0	192	ORF1b	G	V	0	0	0
24862	NL/Katwoude-92722-I	A	G	34.0	53	S	T	T	8.614	0.338	0.463
28115	NL/Delft-92965-I	T	C	47.8	67	ORF8	I	I	100.000	100.000	99.993
28139	NL/Tilburg-94339-I	C	A	36.0	136	ORF8	S	S	0.130	0.113	0.007
28375	NL/Tilburg-94339-I	G	A	30.8	146	N	G	G	0	0	0.002
28394	NL/AmsterdamWest-92852-I	C	T	31.5	54	N	R	W	0	0	0.004
	BE/Properinge-92949-I	C	T	16.7	60	N	R	W	0	0	0.004
	NL/Tilburg-94339-I	C	T	13.6	191	N	R	W	0	0	0.004
28736	BE/Leuven-93034-I	A	G	22.0	363	N	A	T	100.000	100.000	100.000

*BE, Belgium; LFV, low-frequency variant; MV, major variant; NL, Netherlands; SARS-CoV-2, severe acute respiratory syndrome coronavirus 2. †Positions are given with respect to Wuhan-Hu-1 (GenBank accession no. MN908947). ‡Frequency of the LFV of sample against GISAID database (as of July 8, 2020) of the Netherlands, Belgium, and global samples. §Dashes represent a gap at the given region, either as a MV or LFV.

Finding clade-defining mutations in the consensus sequence suggests the dominance of a certain clade within a sample; the presence of these mutations can also aid in the detection of virus mixtures in a sample. During the period of wastewater-sample collection, Nextstrain defined 5 major clades (19A, 19B, 20A, 20B, and 20C). Each clade is defined by the presence of >2 linked mutations. Clade 19A is the root clade and contains the Wuhan-Hu-1 reference sequence. Both 19B and 20A emerged from 19A, where 2 and 3 linked mutations define these major clades: T28144C and C8782T define 19B; and C3037T, C14408T, and A23403G define 20A. Clades 20B and 20C emerged from 20A, where the trinucleotide substitution GGG28881–28883AAC defines 20B and the linked mutations C1059T and G25563T define 20C. Nucleotide substitution A23403G, a signature mutation of clades 20A, 20B, and 20C that generates the D614G amino-acid substitution in the S glycoprotein, was detected in 83.6% (51/61) of the samples that were sequenced at this region (Appendix Table 1). The GGG28881–28883AAC substitution was detected in 41.9% (18/43) of the sequences. One of the 2 mutations defining the low-prevalence clades 20C and 19B (C1059T and T28144C) was found in 2 and 3 consensus sequences. However, these sequences could not be assigned to these clades because regions containing the additional clade-defining mutations were not sequenced with sufficient coverage. The hCoV-19/env/Netherlands/Amersfoort-92503-N/2020 sequence contained a mix of clade-defining mutations: C1059T, which defines 20C; T28144C, which defines 19B; and GGG28881–28883AAC, which defines 20B. This finding indicates that the obtained consensus sequence does not represent a single strain.

In addition to the clade-defining mutations, we detected 49 and 63 SNPs that were not present in either the Netherlands (1,544 sequences) or Belgium (888 sequences) datasets but were seen in the global dataset (55,074 sequences), although with <1% prevalence (Appendix Table 2). Moreover, we detected 51 novel mutations in sewage consensus sequences that were not previously reported, of which 48 were supported by coverage above the thresholds set for high quality (coverage >30× for Nanopore and coverage >5× and Phred score >30 for Illumina). Discrepancies between consensus sequences of the same sewage sample can occur. AmsterdamWest-92852 was sequenced 3 times and 4 positions varied (Appendix Table 1). These differences are explained by the presence of variant sites in a single sample in similar percentages, which resulted in differences in consensus sequences between sequencing runs.

### LFV Analysis

Given that sewage samples are likely to contain a mixture of SARS-CoV-2 strains, we performed a variant analysis with Illumina data to distinguish multiple strains within single samples. By using a coverage >50×, Phred score >30, and a frequency threshold of >10% as settings, we found 21 positions with at least 1 sample containing major and minor variants (Table 2). Of these, 14 mutations resulted in changes at the amino acid level (12 nonsynonymous mutations and 2 deletions). Of note, 8 of these (4497C, 10514C, 11484T, 13046A, 16538_16540delATA, 16777T, 16823T, and 28736A) are novel mutations that did not appear in the Netherlands–Belgium or global datasets. The other 7 variants appeared but demonstrated low prevalence in both datasets (0.002%–0.130%). The most prominent of these was the 28139A mutation in a wastewater sample from March, which was detected in only 4 sequences worldwide and demonstrated both a strong temporal (all detected in March 2020) and regional signal (2 sequences from the Netherlands [EPI_ISL_422640 and EPI_ISL_422880], 1 from Denmark [EPI_ISL_444879], and 1 from Belgium [EPI_ISL_458209]).

Finally, 4 variants (1440A, 11083T, 11109T, and 24862G) appeared at higher levels in both datasets (>0.5%); 11109T and 24862G were 28.5 and 14.3 times more prevalent in the Netherlands dataset than in the global dataset (Table 2). The other variants appeared at similar frequencies in all datasets.

In addition to consensus sequences, LFV analysis is of value identifying potential local outbreaks. This identification could be achieved by detecting cluster-defining mutations that are associated with sequences from a particular geographic area. To associate the presence of a minor variant to sequences belonging to unique clusters, we mapped the 4 most prevalent LFVs onto the Netherlands–Belgium subsample and global subsample phylogenetic trees (Figure 3). For 3 variants (1440A, 11109T, and 24862G), the presence of the mutation and their clustering on the phylogenies were clearly associated. However, when 1 of these 3 variants was detected as an LFV in a sewage sample, the consensus sequence of this sample did not group with the cluster of clinical samples that contains the variant. For example, the 24862G variant in sample Tilburg-94339 was detected in 2 unique clusters within clade 20A, whereas its consensus sequence (hCoV-19/env/Netherlands/Tilburg-94339-I/2020) clustered within clade 20B, suggesting the presence of both clades in this sample. Although mutation 11083T was most prevalent in clade 19A, it was also scattered along the trees, indicating poor association with a particular clade.

## Discussion

The use of wastewater sampling as a tool to learn more about the epidemiology and diversity of SARS-CoV-2 at a community level offers many advantages over human sampling. Sewage samples are relatively easy to collect, sampling bias toward severe cases does not occur, ethical issues are limited, and potentially fewer samples are required to determine temporal changes of viral infections in the community (*35*,*36*). Nevertheless, comprehensive comparisons with clinical surveillance are required to determine the extent and limits of using sewage as a surveillance or early-warning tool.

We used nanopore and Illumina NGS analysis to study the diversity of SARS-CoV-2 in sewage and compared these results to the viral diversity found in clinical samples. To evaluate this diversity in a comprehensive fashion, we used the Nextstrain clade classification system because it is based on the use of signature mutations to assign sequences to a clade (*3*), enabling the association of SNPs or LFV to a particular clade, especially for genome sequences with <75% coverage.

Our method enabled us to obtain complete or near-complete genomes from wastewater samples with C_t_ values of >5 C_t_s below the limit of detection and partial genomes for samples with higher C_t_ values. To increase the percentage of genome covered, a threshold of 10× coverage per position was used to generate consensus sequences from nanopore reads. The error rate with this threshold is <0.03%, and most of the mutations (132/145) listed have a coverage of >30×, which produces an error rate of 1/585,000 nt (*30*).

Of note, we found sewage samples that clustered with sequences isolated from patients of the same region and LFV with a strong regional signal. In a recent study from the United States, wastewater contained SARS-CoV-2 genomes identical to those in clinical samples from the same region (*37*). Sewage samples can contain a mixture of SARS-CoV-2 viruses, which can be an indication of multiple viruses circulating within a community and perhaps in domestic and livestock animals (*38*–*42*). We applied a targeted amplification method and thus did not assess the presence of other viruses. Consensus sequence genomes from a wastewater sample can identify the predominant virus strain in a population, which is suitable for locations with few introductions of the virus (*22*,*23*). However, this approach is not appropriate for a population in which multiple virus strains are circulating in parallel. Moreover, it might lead to artificial consensus genomes that do not represent an existing virus.

NGS analysis can unravel the diversity of viruses within a complex sample such as wastewater, particularly by using unbiased sequencing of the sewage virome (*43*). Nevertheless, the detection of variants of a virus in a single sample can be challenging because of the relatively low number of reads obtained for each virus. Targeted amplification and NGS of a small genome region of the virus of interest to determine the prevalence of virus variants within a single wastewater sample is more sensitive and less expensive; use of this approach has been reported for enteroviruses, human mastadenoviruses, and noroviruses (*12*,*18*,*44*). Because the diversity of SARS-CoV-2 is still limited, however, this approach would not be useful since no single small piece of the genome can reliably differentiate between clades or lineages. However, we demonstrated that some LFVs and SNPs can be linked to particular clusters or clades within trees without the need for a complete genome. To confidently determine the presence of a particular cluster within a sample, at least 2 LFVs associated with the cluster should be present at substantial levels. Furthermore, variant analysis can also be used to monitor the prevalence of biologically relevant mutations, such as D614G, which has been shown to increase infectivity in vitro (*45*) and might be associated with higher transmission and death rates (*46*; M. Cortey, unpub. data, https://www.biorxiv.org/content/10.1101/2020.05.16.099499v1). Within our dataset, clear temporal changes in the prevalence of LFVs or SNPs in sewage samples that correlated with changes in the clinical dataset were not detected during the first wave.

The combination of whole-genome sequencing of clinical samples with epidemiologic data is vital for public health decision-making (*26*) because it helps identify clusters of infection, new introductions of virus, and the expansion and decline of circulating strains. Cities with large numbers of visitors are expected to experience several introductions of the virus, whereas the opposite is expected for cities with low numbers of visitors. The use of NGS analysis of sewage samples to evaluate viral diversity within a geographic area and its changes over time can aid in decision-making. For example, in scenarios in which a large increase of viral diversity is detected in sewage, suggesting new introductions of virus, appropriate measures can be taken.

Wastewater can also be used to monitor novel mutations. Our consensus and LFV analyses revealed 57 mutations that were not seen in the global database. These novel mutations might not have been detected for several reasons: they represent technical errors; the mutations did not stay within the population; or the mutations are associated with asymptomatic or mild disease, viruses from animal hosts, enteric shedding, or defective genomes. The presence of defective genomes has previously been suggested for the detection of LFVs that generate stop codons in clinical samples (*47*). Phenotypic studies could help determine the likelihood and biologic relevance of these novel mutations.

In conclusion, this study illustrates the value of NGS analysis of wastewater to approximate the diversity of SARS-CoV-2 circulating in a community. Sequencing of wastewater samples could be a powerful tool to complement clinical surveillance or could be used independently in settings in which wide clinical sequencing is unfeasible. In addition, in-depth NGS analysis of wastewater samples can help in assessing changes in viral diversity, which can indicate the emergence of epidemiologically or clinically relevant mutations and thereby aid public health decision-making.

Appendix Additional information about monitoring SARS-CoV-2 circulation and diversity through community wastewater sequencing, the Netherlands and Belgium.

## References

[1] Shu Y, McCauley J. GISAID: Global initiative on sharing all influenza data - from vision to reality. Euro Surveill. 2017;22:30494. 10.2807/1560-7917.ES.2017.22.13.30494 28382917 PMC5388101

[2] Rambaut A, Holmes EC, O’Toole Á, Hill V, McCrone JT, Ruis C, et al. A dynamic nomenclature proposal for SARS-CoV-2 lineages to assist genomic epidemiology. Nat Microbiol. 2020;5:1403–7. 10.1038/s41564-020-0770-5 32669681 PMC7610519

[3] Hadfield J, Megill C, Bell SM, Huddleston J, Potter B, Callender C, et al. Nextstrain: real-time tracking of pathogen evolution. Bioinformatics. 2018;34:4121–3. 10.1093/bioinformatics/bty407 29790939 PMC6247931

[4] Lamers MM, Beumer J, van der Vaart J, Knoops K, Puschhof J, Breugem TI, et al. SARS-CoV-2 productively infects human gut enterocytes. Science. 2020;369:50–4. 10.1126/science.abc1669 32358202 PMC7199907

[5] Xiao F, Tang M, Zheng X, Liu Y, Li X, Shan H. Evidence for gastrointestinal infection of SARS-CoV-2. Gastroenterology. 2020;158:1831–1833.e3. 10.1053/j.gastro.2020.02.055 32142773 PMC7130181

[6] Xiao F, Sun J, Xu Y, Li F, Huang X, Li H, et al. Infectious SARS-CoV-2 in feces of patient with severe COVID-19. Emerg Infect Dis. 2020;26:1920–2. 10.3201/eid2608.200681 32421494 PMC7392466

[7] Parasa S, Desai M, Thoguluva Chandrasekar V, Patel HK, Kennedy KF, Roesch T, et al. Prevalence of gastrointestinal symptoms and fecal viral shedding in patients with coronavirus disease 2019: a systematic review and meta-analysis. JAMA Netw Open. 2020;3:e2011335. 10.1001/jamanetworkopen.2020.11335 32525549 PMC7290409

[8] Jones DL, Baluja MQ, Graham DW, Corbishley A, McDonald JE, Malham SK, et al. Shedding of SARS-CoV-2 in feces and urine and its potential role in person-to-person transmission and the environment-based spread of COVID-19. Sci Total Environ. 2020;749:141364. 10.1016/j.scitotenv.2020.141364 32836117 PMC7836549

[9] Foladori P, Cutrupi F, Segata N, Manara S, Pinto F, Malpei F, et al. SARS-CoV-2 from faeces to wastewater treatment: What do we know? A review. Sci Total Environ. 2020;743:140444. 10.1016/j.scitotenv.2020.140444 32649988 PMC7311891

[10] Strubbia S, Phan MVT, Schaeffer J, Koopmans M, Cotten M, Le Guyader FS. Characterization of norovirus and other human enteric viruses in sewage and stool samples through next-generation sequencing. Food Environ Virol. 2019;11:400–9. 10.1007/s12560-019-09402-3 31446609 PMC6848244

[11] Hellmér M, Paxéus N, Magnius L, Enache L, Arnholm B, Johansson A, et al. Detection of pathogenic viruses in sewage provided early warnings of hepatitis A virus and norovirus outbreaks. Appl Environ Microbiol. 2014;80:6771–81. 10.1128/AEM.01981-14 25172863 PMC4249052

[12] Bisseux M, Colombet J, Mirand A, Roque-Afonso A-M, Abravanel F, Izopet J, et al. Monitoring human enteric viruses in wastewater and relevance to infections encountered in the clinical setting: a one-year experiment in central France, 2014 to 2015. Euro Surveill. 2018;23:17–00237. 10.2807/1560-7917.ES.2018.23.7.17-00237 29471623 PMC5824128

[13] Wang W, Xu Y, Gao R, Lu R, Han K, Wu G, et al. Detection of SARS-CoV-2 in different types of clinical specimens. JAMA. 2020;323:1843–4. 10.1001/jama.2020.3786 32159775 PMC7066521

[14] Medema G, Heijnen L, Elsinga G, Italiaander R, Brouwer A. Presence of SARS-coronavirus-2 RNA in sewage and correlation with reported COVID-19 prevalence in the early stage of the epidemic in the Netherlands. Environ Sci Technol Lett. 2020;7:511–6. 10.1021/acs.estlett.0c00357 37566285

[15] Heijnen L, Medema G. Surveillance of influenza A and the pandemic influenza A (H1N1) 2009 in sewage and surface water in the Netherlands. J Water Health. 2011;9:434–42. 10.2166/wh.2011.019 21976191

[16] Patel JC, Diop OM, Gardner T, Chavan S, Jorba J, Wassilak SGF, et al. Surveillance to track progress toward polio eradication—worldwide, 2017–2018. MMWR Morb Mortal Wkly Rep. 2019;68:312–8. 10.15585/mmwr.mm6813a4 30946737 PMC6611474

[17] Suffredini E, Iaconelli M, Equestre M, Valdazo-González B, Ciccaglione AR, Marcantonio C, et al. Genetic diversity among genogroup II noroviruses and progressive emergence of GII.17 in wastewaters in Italy (2011–2016) revealed by next-generation and Sanger sequencing. Food Environ Virol. 2018;10:141–50. 10.1007/s12560-017-9328-y 29185203

[18] Fumian TM, Fioretti JM, Lun JH, Dos Santos IAL, White PA, Miagostovich MP. Detection of norovirus epidemic genotypes in raw sewage using next generation sequencing. Environ Int. 2019;123:282–91. 10.1016/j.envint.2018.11.054 30553201

[19] Wu F, Zhang J, Xiao A, Gu X, Lee WL, Armas F, et al. SARS-CoV-2 titers in wastewater are higher than expected from clinically confirmed cases. mSystems. 2020;5:e00614–20. 10.1128/mSystems.00614-20 32694130 PMC7566278

[20] Randazzo W, Truchado P, Cuevas-Ferrando E, Simón P, Allende A, Sánchez G. SARS-CoV-2 RNA in wastewater anticipated COVID-19 occurrence in a low prevalence area. Water Res. 2020;181:115942. 10.1016/j.watres.2020.115942 32425251 PMC7229723

[21] Wurtzer S, Marechal V, Mouchel JM, Maday Y, Teyssou R, Richard E, et al. Evaluation of lockdown effect on SARS-CoV-2 dynamics through viral genome quantification in waste water, Greater Paris, France, 5 March to 23 April 2020. Euro Surveill. 2020;25:2000776. 10.2807/1560-7917.ES.2020.25.50.2000776 33334397 PMC7812418

[22] Rimoldi SG, Stefani F, Gigantiello A, Polesello S, Comandatore F, Mileto D, et al. Presence and infectivity of SARS-CoV-2 virus in wastewaters and rivers. Sci Total Environ. 2020;744:140911. 10.1016/j.scitotenv.2020.140911 32693284 PMC7358170

[23] Nemudryi A, Nemudraia A, Wiegand T, Surya K, Buyukyoruk M, Cicha C, et al. Temporal detection and phylogenetic assessment of SARS-CoV-2 in municipal wastewater. Cell Rep Med. 2020;1:100098. 10.1016/j.xcrm.2020.100098 32904687 PMC7457911

[24] 2019-novel coronavirus (2019-nCoV) real-time rRT-PCR panel primers and probes [cited 2020 Jul 23]. https://www.fda.gov/media/134922/download

[25] Corman VM, Landt O, Kaiser M, Molenkamp R, Meijer A, Chu DK, et al. Detection of 2019 novel coronavirus (2019-nCoV) by real-time RT-PCR. Euro Surveill. 2020;25:2000045. 10.2807/1560-7917.ES.2020.25.3.2000045 31992387 PMC6988269

[26] Oude Munnink BB, Nieuwenhuijse DF, Stein M, O’Toole Á, Haverkate M, Mollers M, et al.; Dutch-Covid-19 response team. Rapid SARS-CoV-2 whole-genome sequencing and analysis for informed public health decision-making in the Netherlands. Nat Med. 2020;26:1405–10. 10.1038/s41591-020-0997-y 32678356

[27] Richard M, Kok A, de Meulder D, Bestebroer TM, Lamers MM, Okba NMA, et al. SARS-CoV-2 is transmitted via contact and via the air between ferrets. Nat Commun. 2020;11:3496. 10.1038/s41467-020-17367-2 32641684 PMC7343828

[28] Martin M. Cutadapt removes adapter sequences from high-throughput sequencing reads. EMBnet J. 2011;17:10–2. 10.14806/ej.17.1.200

[29] Okonechnikov K, Golosova O, Fursov M; UGENE team. Unipro UGENE: a unified bioinformatics toolkit. Bioinformatics. 2012;28:1166–7. 10.1093/bioinformatics/bts091 22368248

[30] Oude Munnink BB, Nieuwenhuijse DF, Sikkema RS, Koopmans M. Validating whole genome nanopore sequencing, using Usutu virus as an example. J Vis Exp. 2020;157:e60906. 10.3791/60906 32225162

[31] Afgan E, Baker D, Batut B, van den Beek M, Bouvier D, Cech M, et al. The Galaxy platform for accessible, reproducible and collaborative biomedical analyses: 2018 update. Nucleic Acids Res. 2018;46(W1):W537–44. 10.1093/nar/gky379 29790989 PMC6030816

[32] Chen S, Zhou Y, Chen Y, Gu J. fastp: an ultra-fast all-in-one FASTQ preprocessor. Bioinformatics. 2018;34:i884–90. 10.1093/bioinformatics/bty560 30423086 PMC6129281

[33] Grubaugh ND, Gangavarapu K, Quick J, Matteson NL, De Jesus JG, Main BJ, et al. An amplicon-based sequencing framework for accurately measuring intrahost virus diversity using PrimalSeq and iVar. Genome Biol. 2019;20:8. 10.1186/s13059-018-1618-7 30621750 PMC6325816

[34] Wu F, Zhao S, Yu B, Chen Y-M, Wang W, Song Z-G, et al. A new coronavirus associated with human respiratory disease in China. Nature. 2020;579:265–9. 10.1038/s41586-020-2008-3 32015508 PMC7094943

[35] Farkas K, Hillary LS, Malham SK, McDonald JE, Jones DL. Wastewater and public health: the potential of wastewater surveillance for monitoring COVID-19. Curr Opin Environ Sci Health. 2020;17:14–20. 10.1016/j.coesh.2020.06.001 32835157 PMC7291992

[36] Michael-Kordatou I, Karaolia P, Fatta-Kassinos D. Sewage analysis as a tool for the COVID-19 pandemic response and management: the urgent need for optimised protocols for SARS-CoV-2 detection and quantification. J Environ Chem Eng. 2020;8:104306. 10.1016/j.jece.2020.104306 32834990 PMC7384408

[37] Crits-Christoph A, Kantor RS, Olm MR, Whitney ON, Al-Shayeb B, Lou YC, et al. Genome sequencing of sewage detects regionally prevalent SARS-CoV-2 variants. mBio. 2021;12:e02703–20. 10.1128/mBio.02703-20 33468686 PMC7845645

[38] Mykytyn AZ, Lamers MM, Okba NMA, Breugem TI, Schipper D, van den Doel PB, et al. Susceptibility of rabbits to SARS-CoV-2. Emerg Microbes Infect. 2021;10:1–7. 10.1080/22221751.2020.1868951 33356979 PMC7832544

[39] Oreshkova N, Molenaar RJ, Vreman S, Harders F, Oude Munnink BB, Hakze-van der Honing RW, et al. SARS-CoV-2 infection in farmed minks, the Netherlands, April and May 2020. Euro Surveill. 2020;25:2001005. 10.2807/1560-7917.ES.2020.25.23.2001005 32553059 PMC7403642

[40] Halfmann PJ, Hatta M, Chiba S, Maemura T, Fan S, Takeda M, et al. Transmission of SARS-CoV-2 in domestic cats. N Engl J Med. 2020;383:592–4. 10.1056/NEJMc2013400 32402157 PMC9678187

[41] Schlottau K, Rissmann M, Graaf A, Schön J, Sehl J, Wylezich C, et al. SARS-CoV-2 in fruit bats, ferrets, pigs, and chickens: an experimental transmission study. Lancet Microbe. 2020;1:e218–25. 10.1016/S2666-5247(20)30089-6 32838346 PMC7340389

[42] Shi J, Wen Z, Zhong G, Yang H, Wang C, Huang B, et al. Susceptibility of ferrets, cats, dogs, and other domesticated animals to SARS-coronavirus 2. Science. 2020;368:1016–20. 10.1126/science.abb7015 32269068 PMC7164390

[43] Nieuwenhuijse DF, Oude Munnink BB, Phan MVT, Munk P, Venkatakrishnan S, Aarestrup FM, et al.; Global Sewage Surveillance project consortium. Setting a baseline for global urban virome surveillance in sewage. Sci Rep. 2020;10:13748. 10.1038/s41598-020-69869-0 32792677 PMC7426863

[44] Lun JH, Crosbie ND, White PA. Genetic diversity and quantification of human mastadenoviruses in wastewater from Sydney and Melbourne, Australia. Sci Total Environ. 2019;675:305–12. 10.1016/j.scitotenv.2019.04.162 31030137

[45] Zhang L, Jackson CB, Mou H, Ojha A, Peng H, Quinlan BD, et al. SARS-CoV-2 spike-protein D614G mutation increases virion spike density and infectivity. Nat Commun. 2020;11:6013. 10.1038/s41467-020-19808-4 33243994 PMC7693302

[46] Toyoshima Y, Nemoto K, Matsumoto S, Nakamura Y, Kiyotani K. SARS-CoV-2 genomic variations associated with mortality rate of COVID-19. J Hum Genet. 2020;65:1075–82. 10.1038/s10038-020-0808-9 32699345 PMC7375454

[47] Karamitros T, Papadopoulou G, Bousali M, Mexias A, Tsiodras S, Mentis A. SARS-CoV-2 exhibits intra-host genomic plasticity and low-frequency polymorphic quasispecies. J Clin Virol. 2020;131:104585. 10.1016/j.jcv.2020.104585 32818852 PMC7418792

