# Supplementary material for: Monitoring SARS-CoV-2 Circulation and Diversity through Community Wastewater Sequencing, the Netherlands and Belgium
Source: Emerg Infect Dis. 2021 May;27(5):1405–15. doi: 10.3201/eid2705.204410 (PMC8084483; doi:10.3201/eid2705.204410)
Supplement: Appendix — Additional information about monitoring SARS-CoV-2 circulation and diversity through community wastewater sequencing, the Netherlands and Belgium. [file 20-4410-Techapp-s1.pdf]

Article DOI: https://doi.org/10.3201/eid2705.204410

Monitoring SARS-CoV-2 Circulation and Diversity through Community Wastewater Sequencing, the Netherlands and Belgium

Appendix

Appendix Table 1. Summary of all SNPs detected in wastewater samples at the consensus level, the Netherlands and Belgium*

Isolate	104	187	241	331	430	448	493	514	515	702	1059	1191	1338	1594	1605
Wuhan-Hu-1 (MN908947)	T	A	C	G	A	G	T	T	G	G	C	C	G	C	ATG
hCoV-19/env/Netherlands/Amersfoort-92848-I/2020	T	A	T	G	A	G	T	C	G	G	Ø	Ø	Ø	Ø	Ø
hCoV-19/env/Netherlands/Amersfoort-92848-N/2020	T	A	C	G	A	G	T	T	G	G	C	C	G	C	---
hCoV-19/env/Netherlands/Amersfoort-93818-N/2020	Ø	Ø	Ø	Ø	A	G	T	T	G	G	Ø	Ø	Ø	C	ATG
hCoV-19/env/Netherlands/Amersfoort-92503-N/2020	T	A	T	G	A	G	T	T	G	G	C	C	G	C	ATG
hCoV-19/env/Netherlands/AmsterdamWest-92852-I/2020	C	A	C	G	A	G	T	T	G	G	C	C	G	Ø	Ø
hCoV-19/env/Netherlands/AmsterdamWest-92852-N1/2020	T	A	T	G	A	G	T	T	G	G	C	C	G	C	ATG
hCoV-19/env/Netherlands/AmsterdamWest-92852-N2/2020	T	A	T	G	A	G	T	T	G	G	C	C	G	C	ATG
hCoV-19/env/Netherlands/AmsterdamWest-94978-N/2020	Ø	Ø	Ø	Ø	Ø	Ø	Ø	Ø	Ø	Ø	Ø	Ø	Ø	Ø	Ø
hCoV-19/env/Netherlands/AmsterdamWest-93822-N/2020	Ø	Ø	Ø	Ø	A	G	T	T	G	G	T	C	G	C	ATG
hCoV-19/env/Netherlands/AmsterdamWest-95552-N/2020	Ø	Ø	Ø	Ø	A	G	T	T	G	G	Ø	Ø	Ø	Ø	Ø
hCoV-19/env/Netherlands/AmsterdamWest-95794-N/2020	Ø	Ø	Ø	Ø	Ø	Ø	Ø	Ø	Ø	Ø	Ø	Ø	Ø	Ø	Ø
hCoV-19/env/Netherlands/AmsterdamWest-94334-N/2020	Ø	Ø	Ø	Ø	A	G	T	T	G	G	C	C	G	C	ATG
hCoV-19/env/Netherlands/AmsterdamWest-92508-N/2020	T	A	T	G	A	G	T	T	G	G	C	C	G	C	ATG
hCoV-19/env/Netherlands/AmsterdamWest-94604-N/2020	Ø	Ø	Ø	Ø	Ø	Ø	Ø	Ø	Ø	Ø	C	C	G	Ø	Ø
hCoV-19/env/Netherlands/Apeldoorn-92502-I/2020	Ø	Ø	Ø	Ø	Ø	Ø	Ø	Ø	Ø	Ø	C	C	Ø	C	ATG
hCoV-19/env/Netherlands/Apeldoorn-92502-N/2020	T	A	C	G	A	G	Ø	Ø	Ø	Ø	C	C	Ø	C	---
hCoV-19/env/Belgium/Properinge-92949-I/2020	T	A	T	G	A	G	T	T	G	T	C	C	G	C	ATG
hCoV-19/env/Belgium/Properinge-92949-N/2020	T	A	T	G	A	G	Ø	Ø	Ø	Ø	C	C	Ø	C	ATG
hCoV-19/env/Belgium/Aartselaar-93030-I/2020	T	A	T	G	A	G	T	T	G	G	C	C	Ø	Ø	Ø
hCoV-19/env/Belgium/Aartselaar-93030-N/2020	T	A	T	G	A	G	T	T	G	G	C	Ø	Ø	C	ATG
hCoV-19/env/Belgium/Gent-93032-I/2020	T	A	T	G	A	G	T	T	G	G	C	Ø	Ø	Ø	Ø
hCoV-19/env/Belgium/Gent-93032-N/2020	T	A	T	G	A	G	T	T	G	G	C	Ø	Ø	C	ATG
hCoV-19/env/Belgium/Leuven-93034-I/2020	T	A	T	G	A	G	T	Ø	Ø	Ø	C	C	Ø	C	ATG
hCoV-19/env/Belgium/Leuven-93034-N/2020	T	A	T	G	A	G	T	T	G	G	C	C	G	C	ATG
hCoV-19/env/Belgium/Tienen-93036-I/2020	Ø	Ø	T	G	A	G	T	T	G	G	C	Ø	Ø	C	ATG
hCoV-19/env/Belgium/Tienen-93036-N/2020	Ø	Ø	Ø	Ø	Ø	Ø	Ø	Ø	Ø	Ø	C	C	G	C	ATG
hCoV-19/env/Belgium/Langemark-92943-N/2020	T	A	T	G	A	G	T	T	G	G	C	C	G	C	ATG
hCoV-19/env/Belgium/Lo-Reninge-92947-N/2020	Ø	Ø	Ø	Ø	Ø	Ø	Ø	Ø	Ø	Ø	Ø	Ø	Ø	C	ATG
hCoV-19/env/Netherlands/Beverwijk-92721-I/2020	T	A	T	G	A	G	Ø	Ø	Ø	Ø	C	C	G	T	ATG
hCoV-19/env/Netherlands/Beverwijk-92721-N/2020	T	A	T	G	A	G	C	T	G	G	C	C	G	C	ATG

Isolate	104	187	241	331	430	448	493	514	515	702	1059	1191	1338	1594	1605
hCoV-19/env/Netherlands/Delft-92965-I/2020	Ø	A	t	G	A	G	T	T	G	G	C	Ø	Ø	Ø	Ø
hCoV-19/env/Netherlands/Delft-92965-N/2020	T	A	T	G	A	G	T	T	G	G	C	C	G	C	ATG
hCoV-19/env/Netherlands/Delft-97044-N/2020	Ø	Ø	Ø	Ø	Ø	Ø	Ø	Ø	Ø	Ø	Ø	Ø	Ø	Ø	Ø
hCoV-19/env/Netherlands/Delft-94982-N/2020	Ø	Ø	Ø	Ø	A	G	T	T	G	G	C	C	G	Ø	Ø
hCoV-19/env/Netherlands/Delft-93825-I/2020	Ø	A	T	G	A	G	T	T	G	G	C	Ø	Ø	C	ATG
hCoV-19/env/Netherlands/Delft-93825-N/2020	T	A	T	G	A	G	T	T	G	G	Ø	Ø	Ø	C	---
hCoV-19/env/Netherlands/Delft-94337-N/2020	Ø	Ø	Ø	Ø	Ø	Ø	Ø	Ø	Ø	Ø	Ø	Ø	Ø	Ø	Ø
hCoV-19/env/Netherlands/Delft-94607-N/2020	Ø	Ø	Ø	Ø	Ø	Ø	Ø	Ø	Ø	Ø	Ø	C	G	Ø	Ø
hCoV-19/env/Netherlands/Franeker-92719-I/2020	Ø	A	T	G	A	G	T	T	G	G	C	C	G	C	ATG
hCoV-19/env/Netherlands/Franeker-92719-N1/2020	T	A	T	G	A	G	T	T	G	G	C	C	G	C	ATG
hCoV-19/env/Netherlands/Franeker-92719-N2/2020	Ø	Ø	Ø	Ø	A	G	T	T	G	G	C	C	G	Ø	Ø
hCoV-19/env/Netherlands/HeeswijkDinther-93948-N/2020	Ø	Ø	Ø	Ø	A	G	T	T	G	G	Ø	Ø	Ø	Ø	Ø
hCoV-19/env/Netherlands/HeeswijkDinther-92499-N/2020	T	A	T	G	A	G	T	T	G	G	C	C	G	C	ATG
hCoV-19/env/Netherlands/Katwoude-92722-I/2020	T	A	T	G	A	G	T	T	G	G	C	C	T	C	ATG
hCoV-19/env/Netherlands/Katwoude-92722-N/2020	T	A	T	T	A	G	T	T	G	G	C	C	G	C	ATG
hCoV-19/env/Netherlands/Schiphol-92851-I/2020	T	A	T	G	A	G	T	T	G	G	C	C	G	C	ATG
hCoV-19/env/Netherlands/Schiphol-92851-N/2020	T	G	T	G	A	G	T	C	G	G	T	C	G	C	ATG
hCoV-19/env/Netherlands/Schiphol-96927-I/2020	Ø	Ø	Ø	Ø	A	G	T	T	G	G	Ø	Ø	Ø	C	ATG
hCoV-19/env/Netherlands/Schiphol-96927-N/2020	Ø	Ø	Ø	Ø	Ø	Ø	Ø	Ø	Ø	Ø	Ø	Ø	Ø	Ø	Ø
hCoV-19/env/Netherlands/Schiphol-93823-I/2020	Ø	A	T	G	A	G	T	Ø	Ø	G	C	C	Ø	C	ATG
hCoV-19/env/Netherlands/Schiphol-93823-N/2020	T	A	T	G	A	G	T	T	G	G	T	Ø	Ø	Ø	Ø
hCoV-19/env/Netherlands/Schiphol-94335-I/2020	Ø	Ø	Ø	Ø	A	T	T	T	G	G	C	C	Ø	Ø	Ø
hCoV-19/env/Netherlands/Schiphol-94335-N/2020	T	A	T	G	A	G	Ø	Ø	Ø	Ø	C	C	G	C	ATG
hCoV-19/env/Netherlands/Schiphol-92506-I/2020	Ø	A	T	G	A	G	T	T	G	G	C	C	G	C	ATG
hCoV-19/env/Netherlands/Schiphol-92506-N/2020	T	A	T	G	A	G	T	T	G	G	C	C	G	C	ATG
hCoV-19/env/Netherlands/Schiphol-94605-I/2020	Ø	Ø	Ø	Ø	C	G	T	T	G	G	C	Ø	G	Ø	Ø
hCoV-19/env/Netherlands/Schiphol-94605-N/2020	Ø	Ø	Ø	Ø	A	G	T	T	G	G	Ø	Ø	Ø	Ø	Ø
hCoV-19/env/Netherlands/Tilburg-92853-I/2020	T	A	C	G	A	G	T	T	G	G	C	C	G	C	---
hCoV-19/env/Netherlands/Tilburg-92853-N/2020	Ø	Ø	Ø	Ø	A	G	T	C	G	G	C	C	G	C	ATG
hCoV-19/env/Netherlands/Tilburg-93828-N/2020	Ø	Ø	Ø	Ø	A	G	T	T	G	G	Ø	Ø	Ø	Ø	Ø
hCoV-19/env/Netherlands/Tilburg-94339-I/2020	Ø	A	T	G	A	G	T	T	G	G	C	C	T	C	ATG
hCoV-19/env/Netherlands/Tilburg-94339-N/2020	Ø	Ø	Ø	Ø	Ø	Ø	Ø	Ø	Ø	Ø	Ø	Ø	Ø	Ø	Ø
hCoV-19/env/Netherlands/Tilburg-92509-I/2020	T	A	C	G	A	G	T	C	G	G	C	C	Ø	C	---
hCoV-19/env/Netherlands/Tilburg-92509-N/2020	T	A	C	G	A	G	T	T	G	G	C	C	G	C	ATG
hCoV-19/env/Netherlands/UtrechtOvervecht-92505-N/2020	T	A	T	G	A	G	T	T	G	G	C	T	G	C	ATG
hCoV-19/env/Netherlands/Utrecht-92849-I/2020	T	A	T	G	A	G	T	T	G	G	C	C	Ø	Ø	Ø
hCoV-19/env/Netherlands/Utrecht-92849-N/2020	Ø	Ø	Ø	Ø	A	G	T	T	G	G	C	C	G	Ø	Ø
hCoV-19/env/Netherlands/Utrecht-94976-N/2020	Ø	Ø	Ø	Ø	Ø	Ø	Ø	Ø	Ø	Ø	Ø	Ø	Ø	Ø	Ø
hCoV-19/env/Netherlands/Utrecht-93820-N/2020	Ø	Ø	Ø	Ø	A	G	T	T	G	G	Ø	Ø	Ø	C	ATG
hCoV-19/env/Netherlands/Utrecht-95550-N/2020	Ø	Ø	Ø	Ø	Ø	Ø	Ø	Ø	Ø	Ø	Ø	Ø	Ø	Ø	Ø
hCoV-19/env/Netherlands/Utrecht-94330-N/2020	Ø	Ø	Ø	Ø	A	G	T	T	G	G	C	C	G	Ø	Ø
hCoV-19/env/Netherlands/Utrecht-94331-N/2020	T	A	T	G	A	G	Ø	Ø	Ø	Ø	Ø	Ø	Ø	Ø	Ø
hCoV-19/env/Netherlands/Utrecht-92504-N/2020	T	A	T	G	A	G	T	T	G	G	C	C	G	C	ATG
hCoV-19/env/Netherlands/Utrecht-94602-N/2020	Ø	Ø	Ø	Ø	A	G	T	T	G	G	C	Ø	Ø	Ø	Ø
hCoV-19/env/Netherlands/Wervershoof-93950-N/2020	T	A	T	G	A	G	T	T	G	G	Ø	Ø	Ø	Ø	Ø
hCoV-19/env/Netherlands/Wervershoof-92723-I/2020	T	A	T	G	A	G	T	C	T	G	Ø	Ø	Ø	C	ATG
hCoV-19/env/Netherlands/Wervershoof-92723-N/2020	T	A	T	G	A	G	T	T	G	G	C	C	G	C	---

Isolate	1704	1802	1839	1944	1997	2144	2527	3025	3037	3194	3373	3521	3778	4391
Wuhan-Hu-1 (MN908947)	T	A	A	G	C	G	G	G	C	G	C	G	A	C
hCoV-19/env/Netherlands/Amersfoort-92848-I/2020	Ø	Ø	Ø	Ø	Ø	Ø	Ø	Ø	Ø	Ø	Ø	Ø	Ø	Ø
hCoV-19/env/Netherlands/Amersfoort-92848-N/2020	T	A	A	G	T	G	G	G	T	G	C	G	A	C
hCoV-19/env/Netherlands/Amersfoort-93818-N/2020	T	A	A	Ø	Ø	Ø	Ø	Ø	Ø	Ø	Ø	Ø	Ø	C
hCoV-19/env/Netherlands/Amersfoort-92503-N/2020	T	A	A	G	C	G	G	Ø	Ø	G	C	G	A	Ø
hCoV-19/env/Netherlands/AmsterdamWest-92852-I/2020	Ø	Ø	Ø	Ø	Ø	Ø	Ø	Ø	Ø	G	C	G	A	C
hCoV-19/env/Netherlands/AmsterdamWest-92852-N1/2020	T	A	A	G	C	G	G	G	T	G	C	G	A	C
hCoV-19/env/Netherlands/AmsterdamWest-92852-N2/2020	T	A	A	T	C	G	G	G	T	G	C	G	A	C
hCoV-19/env/Netherlands/AmsterdamWest-94978-N/2020	Ø	Ø	Ø	Ø	Ø	Ø	Ø	Ø	Ø	Ø	Ø	Ø	Ø	Ø
hCoV-19/env/Netherlands/AmsterdamWest-93822-N/2020	T	A	A	Ø	Ø	G	G	T	C	G	C	G	A	C
hCoV-19/env/Netherlands/AmsterdamWest-95552-N/2020	Ø	Ø	Ø	Ø	Ø	Ø	Ø	Ø	Ø	Ø	Ø	Ø	Ø	Ø
hCoV-19/env/Netherlands/AmsterdamWest-95794-N/2020	Ø	A	A	G	C	G	Ø	Ø	Ø	Ø	Ø	Ø	Ø	Ø
hCoV-19/env/Netherlands/AmsterdamWest-94334-N/2020	T	A	A	G	C	G	Ø	Ø	Ø	T	C	G	Ø	C
hCoV-19/env/Netherlands/AmsterdamWest-92508-N/2020	T	A	A	G	C	G	G	G	C	G	C	G	A	C
hCoV-19/env/Netherlands/AmsterdamWest-94604-N/2020	Ø	Ø	Ø	Ø	Ø	Ø	Ø	Ø	Ø	Ø	Ø	Ø	Ø	Ø
hCoV-19/env/Netherlands/Apeldoorn-92502-I/2020	T	A	A	Ø	Ø	G	T	G	T	G	Ø	Ø	Ø	Ø
hCoV-19/env/Netherlands/Apeldoorn-92502-N/2020	T	A	A	G	C	A	G	Ø	Ø	G	C	G	A	T
hCoV-19/env/Belgium/Properinge-92949-I/2020	T	A	A	Ø	Ø	G	G	Ø	Ø	Ø	Ø	G	A	C
hCoV-19/env/Belgium/Properinge-92949-N/2020	A	A	A	Ø	Ø	G	G	G	T	G	Ø	G	A	C
hCoV-19/env/Belgium/Aartselaar-93030-I/2020	Ø	Ø	Ø	Ø	Ø	Ø	Ø	Ø	Ø	G	C	Ø	Ø	C
hCoV-19/env/Belgium/Aartselaar-93030-N/2020	T	A	A	G	C	G	G	G	T	G	C	G	A	C
hCoV-19/env/Belgium/Gent-93032-I/2020	Ø	Ø	Ø	Ø	Ø	Ø	Ø	Ø	Ø	Ø	Ø	Ø	Ø	Ø
hCoV-19/env/Belgium/Gent-93032-N/2020	T	A	A	Ø	Ø	Ø	Ø	Ø	Ø	G	C	G	Ø	C
hCoV-19/env/Belgium/Leuven-93034-I/2020	T	A	A	Ø	Ø	Ø	Ø	Ø	Ø	Ø	Ø	Ø	Ø	C
hCoV-19/env/Belgium/Leuven-93034-N/2020	T	A	A	Ø	Ø	Ø	Ø	Ø	Ø	G	C	G	Ø	C
hCoV-19/env/Belgium/Tienen-93036-I/2020	T	A	C	Ø	Ø	G	G	Ø	Ø	Ø	Ø	G	Ø	Ø
hCoV-19/env/Belgium/Tienen-93036-N/2020	T	A	A	G	C	G	G	Ø	Ø	G	C	G	A	C
hCoV-19/env/Belgium/Langemark-92943-N/2020	T	A	A	Ø	Ø	Ø	Ø	G	T	G	Ø	Ø	Ø	C
hCoV-19/env/Belgium/Lo-Reninge-92947-N/2020	T	A	A	Ø	Ø	Ø	G	Ø	Ø	G	C	G	A	C
hCoV-19/env/Netherlands/Beverwijk-92721-I/2020	T	A	A	Ø	Ø	Ø	Ø	Ø	Ø	Ø	Ø	G	Ø	C
hCoV-19/env/Netherlands/Beverwijk-92721-N/2020	T	A	A	G	C	G	G	Ø	Ø	G	C	G	A	C
hCoV-19/env/Netherlands/Delft-92965-I/2020	Ø	Ø	Ø	Ø	Ø	Ø	Ø	Ø	Ø	G	Ø	G	Ø	Ø
hCoV-19/env/Netherlands/Delft-92965-N/2020	T	A	A	G	C	G	G	G	T	G	C	G	A	Ø
hCoV-19/env/Netherlands/Delft-97044-N/2020	Ø	Ø	Ø	Ø	Ø	Ø	Ø	Ø	Ø	Ø	Ø	Ø	Ø	Ø
hCoV-19/env/Netherlands/Delft-94982-N/2020	Ø	Ø	Ø	Ø	Ø	Ø	Ø	Ø	Ø	G	C	G	Ø	Ø
hCoV-19/env/Netherlands/Delft-93825-I/2020	T	A	A	Ø	Ø	Ø	T	Ø	Ø	Ø	Ø	G	A	Ø
hCoV-19/env/Netherlands/Delft-93825-N/2020	T	A	A	G	C	G	Ø	Ø	Ø	G	C	G	Ø	C
hCoV-19/env/Netherlands/Delft-94337-N/2020	Ø	Ø	Ø	Ø	Ø	Ø	G	Ø	Ø	G	C	G	Ø	Ø

Isolate	1704	1802	1839	1944	1997	2144	2527	3025	3037	3194	3373	3521	3778	4391
hCoV-19/env/Netherlands/Delft-94607-N/2020	Ø	Ø	Ø	Ø	Ø	Ø	Ø	Ø	Ø	Ø	Ø	Ø	Ø	Ø
hCoV-19/env/Netherlands/Franeker-92719-I/2020	T	A	Ø	Ø	Ø	Ø	T	G	T	G	A	G	A	C
hCoV-19/env/Netherlands/Franeker-92719-N1/2020	T	A	A	G	C	G	G	G	T	G	A	G	A	C
hCoV-19/env/Netherlands/Franeker-92719-N2/2020	Ø	Ø	Ø	Ø	Ø	Ø	G	Ø	Ø	G	A	G	G	Ø
hCoV-19/env/Netherlands/HeeswijkDinther-93948-N/2020	Ø	Ø	Ø	Ø	Ø	Ø	Ø	Ø	Ø	Ø	Ø	Ø	Ø	Ø
hCoV-19/env/Netherlands/HeeswijkDinther-92499-N/2020	T	A	A	G	C	G	G	Ø	Ø	G	C	G	A	Ø
hCoV-19/env/Netherlands/Katwoude-92722-I/2020	T	A	A	Ø	Ø	G	G	Ø	Ø	Ø	Ø	Ø	Ø	Ø
hCoV-19/env/Netherlands/Katwoude-92722-N/2020	T	A	A	G	C	G	G	G	T	G	C	G	A	C
hCoV-19/env/Netherlands/Schiphol-92851-I/2020	T	A	A	Ø	Ø	Ø	G	Ø	Ø	Ø	Ø	Ø	A	Ø
hCoV-19/env/Netherlands/Schiphol-92851-N/2020	T	A	A	G	C	G	G	G	T	G	C	G	A	C
hCoV-19/env/Netherlands/Schiphol-96927-I/2020	T	G	A	Ø	Ø	Ø	Ø	Ø	Ø	Ø	Ø	G	Ø	C
hCoV-19/env/Netherlands/Schiphol-96927-N/2020	Ø	G	A	G	C	G	G	Ø	Ø	G	C	G	Ø	Ø
hCoV-19/env/Netherlands/Schiphol-93823-I/2020	T	A	Ø	Ø	Ø	Ø	G	Ø	Ø	Ø	Ø	G	A	Ø
hCoV-19/env/Netherlands/Schiphol-93823-N/2020	Ø	A	A	G	C	G	Ø	Ø	Ø	Ø	Ø	Ø	Ø	C
hCoV-19/env/Netherlands/Schiphol-94335-I/2020	Ø	Ø	Ø	Ø	Ø	Ø	Ø	Ø	Ø	Ø	Ø	Ø	Ø	Ø
hCoV-19/env/Netherlands/Schiphol-94335-N/2020	T	A	A	G	C	G	G	Ø	Ø	G	C	G	Ø	Ø
hCoV-19/env/Netherlands/Schiphol-92506-I/2020	T	A	A	Ø	Ø	G	G	Ø	Ø	G	C	G	A	C
hCoV-19/env/Netherlands/Schiphol-92506-N/2020	T	A	A	G	C	G	G	G	T	G	C	G	A	C
hCoV-19/env/Netherlands/Schiphol-94605-I/2020	Ø	Ø	Ø	Ø	Ø	Ø	Ø	Ø	Ø	G	C	G	Ø	Ø
hCoV-19/env/Netherlands/Schiphol-94605-N/2020	Ø	Ø	Ø	Ø	Ø	Ø	Ø	Ø	Ø	G	C	G	Ø	Ø
hCoV-19/env/Netherlands/Tilburg-92853-I/2020	T	A	A	Ø	Ø	G	G	Ø	Ø	Ø	Ø	G	A	Ø
hCoV-19/env/Netherlands/Tilburg-92853-N/2020	T	A	A	Ø	Ø	G	G	G	C	G	C	G	A	C
hCoV-19/env/Netherlands/Tilburg-93828-N/2020	Ø	Ø	Ø	Ø	Ø	Ø	Ø	Ø	Ø	Ø	Ø	Ø	Ø	Ø
hCoV-19/env/Netherlands/Tilburg-94339-I/2020	T	A	Ø	Ø	Ø	Ø	T	G	T	Ø	C	G	A	C
hCoV-19/env/Netherlands/Tilburg-94339-N/2020	Ø	Ø	Ø	Ø	Ø	Ø	Ø	Ø	Ø	Ø	Ø	Ø	Ø	Ø
hCoV-19/env/Netherlands/Tilburg-92509-I/2020	T	A	Ø	Ø	Ø	Ø	Ø	Ø	Ø	Ø	Ø	Ø	A	Ø
hCoV-19/env/Netherlands/Tilburg-92509-N/2020	T	A	A	Ø	Ø	G	G	G	C	G	C	T	A	C
hCoV-19/env/Netherlands/UtrechtOvervecht-92505-N/2020	T	A	A	Ø	Ø	Ø	Ø	Ø	Ø	G	C	G	A	C
hCoV-19/env/Netherlands/Utrecht-92849-I/2020	T	Ø	Ø	Ø	Ø	G	Ø	Ø	Ø	Ø	Ø	G	Ø	Ø
hCoV-19/env/Netherlands/Utrecht-92849-N/2020	Ø	A	A	G	C	G	G	Ø	Ø	G	C	G	Ø	Ø
hCoV-19/env/Netherlands/Utrecht-94976-N/2020	Ø	Ø	Ø	Ø	Ø	Ø	Ø	Ø	Ø	Ø	Ø	Ø	Ø	Ø
hCoV-19/env/Netherlands/Utrecht-93820-N/2020	T	A	A	Ø	Ø	Ø	Ø	Ø	Ø	Ø	Ø	G	A	C
hCoV-19/env/Netherlands/Utrecht-95550-N/2020	Ø	Ø	Ø	Ø	Ø	Ø	Ø	Ø	Ø	G	C	G	Ø	Ø
hCoV-19/env/Netherlands/Utrecht-94330-N/2020	Ø	Ø	Ø	Ø	Ø	Ø	Ø	Ø	Ø	Ø	Ø	Ø	Ø	C
hCoV-19/env/Netherlands/Utrecht-94331-N/2020	Ø	Ø	Ø	Ø	Ø	Ø	Ø	Ø	Ø	Ø	Ø	Ø	Ø	Ø
hCoV-19/env/Netherlands/Utrecht-92504-N/2020	T	A	A	G	C	G	G	Ø	Ø	G	C	G	A	C
hCoV-19/env/Netherlands/Utrecht-94602-N/2020	Ø	Ø	Ø	Ø	Ø	Ø	Ø	Ø	Ø	Ø	Ø	Ø	Ø	Ø
hCoV-19/env/Netherlands/Wervershoof-93950-N/2020	Ø	Ø	Ø	Ø	Ø	Ø	Ø	Ø	Ø	G	Ø	G	A	C

Isolate	1704	1802	1839	1944	1997	2144	2527	3025	3037	3194	3373	3521	3778	4391
hCoV-19/env/Netherlands/Wervershoof-92723-I/2020	T	A	A	Ø	Ø	Ø	Ø	Ø	Ø	G	C	G	Ø	Ø
hCoV-19/env/Netherlands/Wervershoof-92723-N/2020	T	A	A	G	C	G	G	G	T	G	C	G	A	C

Isolate	4655	4720	4870	5230	5633	6075	6077	7063	7577	8266	8290	8322	8946	9063
Wuhan-Hu-1 (MN908947)	C	G	A	G	G	T	G	C	G	C	C	G	A	G
hCoV-19/env/Netherlands/Amersfoort-92848-I/2020	C	G	A	Ø	Ø	Ø	Ø	Ø	G	Ø	Ø	Ø	Ø	Ø
hCoV-19/env/Netherlands/Amersfoort-92848-N/2020	C	G	A	G	G	T	G	C	G	C	C	G	A	G
hCoV-19/env/Netherlands/Amersfoort-93818-N/2020	Ø	Ø	A	G	Ø	Ø	Ø	Ø	G	Ø	Ø	Ø	A	A
hCoV-19/env/Netherlands/Amersfoort-92503-N/2020	C	G	A	G	G	T	G	C	G	C	C	G	A	G
hCoV-19/env/Netherlands/AmsterdamWest-92852-I/2020	C	G	A	G	G	T	G	C	G	Ø	Ø	Ø	A	G
hCoV-19/env/Netherlands/AmsterdamWest-92852-N1/2020	C	G	A	G	G	T	G	C	G	C	C	G	A	G
hCoV-19/env/Netherlands/AmsterdamWest-92852-N2/2020	C	G	A	G	G	T	G	C	G	C	C	G	A	G
hCoV-19/env/Netherlands/AmsterdamWest-94978-N/2020	Ø	Ø	Ø	G	G	Ø	Ø	Ø	Ø	Ø	Ø	Ø	Ø	Ø
hCoV-19/env/Netherlands/AmsterdamWest-93822-N/2020	C	G	A	G	G	Ø	Ø	C	G	C	C	G	A	G
hCoV-19/env/Netherlands/AmsterdamWest-95552-N/2020	Ø	Ø	Ø	Ø	Ø	Ø	Ø	Ø	Ø	Ø	Ø	Ø	Ø	Ø
hCoV-19/env/Netherlands/AmsterdamWest-95794-N/2020	Ø	Ø	Ø	Ø	Ø	Ø	Ø	Ø	Ø	Ø	Ø	Ø	Ø	Ø
hCoV-19/env/Netherlands/AmsterdamWest-94334-N/2020	C	G	A	G	G	T	G	Ø	G	C	C	G	A	G
hCoV-19/env/Netherlands/AmsterdamWest-92508-N/2020	C	T	A	G	G	T	G	C	G	C	C	G	A	G
hCoV-19/env/Netherlands/AmsterdamWest-94604-N/2020	Ø	Ø	Ø	Ø	Ø	Ø	Ø	Ø	G	C	C	G	A	Ø
hCoV-19/env/Netherlands/Apeldoorn-92502-I/2020	Ø	Ø	Ø	Ø	Ø	Ø	Ø	Ø	Ø	Ø	Ø	Ø	Ø	Ø
hCoV-19/env/Netherlands/Apeldoorn-92502-N/2020	C	G	A	Ø	G	T	G	C	G	C	T	G	A	G
hCoV-19/env/Belgium/Properinge-92949-I/2020	C	G	A	Ø	G	T	G	Ø	G	Ø	Ø	Ø	Ø	Ø
hCoV-19/env/Belgium/Properinge-92949-N/2020	C	G	A	G	Ø	Ø	Ø	C	Ø	Ø	Ø	Ø	A	Ø
hCoV-19/env/Belgium/Aartselaar-93030-I/2020	C	Ø	A	G	Ø	T	G	Ø	G	C	Ø	Ø	A	G
hCoV-19/env/Belgium/Aartselaar-93030-N/2020	C	G	A	G	G	T	G	C	G	C	C	G	A	G
hCoV-19/env/Belgium/Gent-93032-I/2020	C	G	A	Ø	G	Ø	Ø	Ø	G	Ø	Ø	Ø	Ø	Ø
hCoV-19/env/Belgium/Gent-93032-N/2020	C	G	A	G	G	Ø	Ø	Ø	G	C	C	G	A	Ø
hCoV-19/env/Belgium/Leuven-93034-I/2020	Ø	Ø	Ø	Ø	Ø	Ø	Ø	Ø	G	Ø	Ø	Ø	T	G
hCoV-19/env/Belgium/Leuven-93034-N/2020	C	G	A	G	G	T	G	Ø	G	C	C	G	A	G
hCoV-19/env/Belgium/Tienen-93036-I/2020	C	Ø	A	Ø	Ø	Ø	Ø	Ø	G	Ø	Ø	Ø	Ø	Ø
hCoV-19/env/Belgium/Tienen-93036-N/2020	C	G	A	G	G	T	G	C	G	C	C	G	A	G
hCoV-19/env/Belgium/Langemark-92943-N/2020	C	G	A	G	T	T	G	Ø	G	C	C	G	A	G
hCoV-19/env/Belgium/Lo-Reninge-92947-N/2020	C	G	A	G	G	T	G	C	Ø	C	C	G	A	Ø
hCoV-19/env/Netherlands/Beverwijk-92721-I/2020	C	G	A	G	G	Ø	Ø	Ø	Ø	Ø	Ø	Ø	Ø	Ø
hCoV-19/env/Netherlands/Beverwijk-92721-N/2020	C	G	A	G	G	T	G	Ø	G	C	C	G	A	G
hCoV-19/env/Netherlands/Delft-92965-I/2020	C	G	A	G	Ø	Ø	Ø	Ø	G	Ø	Ø	Ø	Ø	G

Isolate	4655	4720	4870	5230	5633	6075	6077	7063	7577	8266	8290	8322	8946	9063
hCoV-19/env/Netherlands/Delft-92965-N/2020	C	G	A	G	G	Ø	Ø	C	G	C	C	G	A	G
hCoV-19/env/Netherlands/Delft-97044-N/2020	C	G	A	Ø	Ø	Ø	Ø	Ø	Ø	Ø	Ø	Ø	Ø	Ø
hCoV-19/env/Netherlands/Delft-94982-N/2020	Ø	Ø	Ø	Ø	Ø	Ø	Ø	Ø	Ø	Ø	Ø	Ø	Ø	Ø
hCoV-19/env/Netherlands/Delft-93825-I/2020	T	G	A	G	G	Ø	Ø	Ø	G	Ø	Ø	Ø	A	G
hCoV-19/env/Netherlands/Delft-93825-N/2020	Ø	Ø	A	G	G	Ø	Ø	T	Ø	C	C	G	A	G
hCoV-19/env/Netherlands/Delft-94337-N/2020	C	G	Ø	Ø	Ø	Ø	Ø	Ø	Ø	Ø	Ø	Ø	Ø	Ø
hCoV-19/env/Netherlands/Delft-94607-N/2020	Ø	Ø	Ø	Ø	Ø	Ø	Ø	Ø	Ø	Ø	Ø	Ø	Ø	Ø
hCoV-19/env/Netherlands/Franeker-92719-I/2020	C	G	A	G	G	T	G	Ø	G	Ø	Ø	Ø	A	G
hCoV-19/env/Netherlands/Franeker-92719-N1/2020	C	G	A	G	G	T	G	C	G	C	C	G	A	G
hCoV-19/env/Netherlands/Franeker-92719-N2/2020	C	G	A	G	G	T	G	Ø	G	Ø	Ø	Ø	Ø	Ø
hCoV-19/env/Netherlands/HeeswijkDinther-93948-N/2020	C	G	A	G	G	Ø	Ø	Ø	Ø	Ø	Ø	Ø	Ø	Ø
hCoV-19/env/Netherlands/HeeswijkDinther-92499-N/2020	C	G	A	G	G	T	G	C	G	C	C	G	A	G
hCoV-19/env/Netherlands/Katwoude-92722-I/2020	C	G	A	G	G	T	G	Ø	G	Ø	Ø	Ø	Ø	Ø
hCoV-19/env/Netherlands/Katwoude-92722-N/2020	C	G	A	G	G	T	G	Ø	G	Ø	Ø	Ø	A	G
hCoV-19/env/Netherlands/Schiphol-92851-I/2020	C	G	A	Ø	Ø	Ø	Ø	Ø	G	Ø	Ø	Ø	Ø	G
hCoV-19/env/Netherlands/Schiphol-92851-N/2020	C	G	A	G	G	T	G	C	G	C	C	T	A	G
hCoV-19/env/Netherlands/Schiphol-96927-I/2020	C	G	A	Ø	Ø	T	G	Ø	Ø	Ø	Ø	Ø	Ø	Ø
hCoV-19/env/Netherlands/Schiphol-96927-N/2020	Ø	Ø	Ø	G	G	Ø	Ø	Ø	Ø	Ø	Ø	Ø	Ø	Ø
hCoV-19/env/Netherlands/Schiphol-93823-I/2020	Ø	Ø	A	Ø	Ø	Ø	Ø	Ø	G	Ø	Ø	Ø	Ø	G
hCoV-19/env/Netherlands/Schiphol-93823-N/2020	T	G	G	G	G	Ø	Ø	C	G	C	C	G	A	G
hCoV-19/env/Netherlands/Schiphol-94335-I/2020	T	G	A	Ø	Ø	Ø	Ø	Ø	G	Ø	Ø	Ø	A	G
hCoV-19/env/Netherlands/Schiphol-94335-N/2020	Ø	Ø	Ø	Ø	Ø	T	G	Ø	Ø	Ø	Ø	Ø	Ø	Ø
hCoV-19/env/Netherlands/Schiphol-92506-I/2020	C	G	A	G	G	T	G	Ø	G	C	C	G	Ø	G
hCoV-19/env/Netherlands/Schiphol-92506-N/2020	C	G	A	G	G	T	G	C	G	C	C	G	A	G
hCoV-19/env/Netherlands/Schiphol-94605-I/2020	T	G	A	Ø	Ø	Ø	Ø	Ø	G	Ø	Ø	Ø	Ø	Ø
hCoV-19/env/Netherlands/Schiphol-94605-N/2020	Ø	Ø	Ø	G	G	Ø	Ø	Ø	Ø	C	C	G	Ø	Ø
hCoV-19/env/Netherlands/Tilburg-92853-I/2020	C	G	A	Ø	G	Ø	Ø	Ø	G	Ø	Ø	Ø	A	G
hCoV-19/env/Netherlands/Tilburg-92853-N/2020	C	G	A	G	G	Ø	Ø	Ø	G	T	C	G	A	G
hCoV-19/env/Netherlands/Tilburg-93828-N/2020	C	G	A	G	G	Ø	Ø	Ø	Ø	Ø	Ø	Ø	Ø	Ø
hCoV-19/env/Netherlands/Tilburg-94339-I/2020	C	G	A	Ø	G	T	G	Ø	G	Ø	Ø	Ø	A	G
hCoV-19/env/Netherlands/Tilburg-94339-N/2020	Ø	Ø	Ø	Ø	Ø	T	G	Ø	Ø	Ø	Ø	Ø	Ø	Ø
hCoV-19/env/Netherlands/Tilburg-92509-I/2020	C	G	A	Ø	Ø	Ø	Ø	Ø	G	Ø	Ø	Ø	A	G
hCoV-19/env/Netherlands/Tilburg-92509-N/2020	C	G	A	T	G	T	G	C	T	Ø	Ø	Ø	A	Ø
hCoV-19/env/Netherlands/UtrechtOvervecht-92505-N/2020	C	G	A	G	G	T	G	C	G	C	C	G	A	G
hCoV-19/env/Netherlands/Utrecht-92849-I/2020	C	Ø	A	Ø	G	Ø	Ø	Ø	G	Ø	Ø	Ø	A	G
hCoV-19/env/Netherlands/Utrecht-92849-N/2020	Ø	Ø	Ø	G	G	T	G	Ø	G	Ø	Ø	Ø	A	Ø
hCoV-19/env/Netherlands/Utrecht-94976-N/2020	Ø	Ø	Ø	Ø	Ø	Ø	Ø	Ø	Ø	Ø	Ø	Ø	Ø	Ø
hCoV-19/env/Netherlands/Utrecht-93820-N/2020	C	G	A	G	G	Ø	Ø	C	G	Ø	Ø	Ø	A	G

Isolate	4655	4720	4870	5230	5633	6075	6077	7063	7577	8266	8290	8322	8946	9063
hCoV-19/env/Netherlands/Utrecht-95550-N/2020	Ø	Ø	Ø	Ø	Ø	Ø	Ø	Ø	Ø	Ø	Ø	Ø	Ø	Ø
hCoV-19/env/Netherlands/Utrecht-94330-N/2020	Ø	Ø	Ø	Ø	G	Ø	Ø	Ø	Ø	Ø	Ø	Ø	Ø	Ø
hCoV-19/env/Netherlands/Utrecht-94331-N/2020	Ø	Ø	Ø	Ø	Ø	Ø	Ø	Ø	G	Ø	Ø	Ø	A	G
hCoV-19/env/Netherlands/Utrecht-92504-N/2020	C	G	A	G	G	T	G	C	G	C	C	G	A	G
hCoV-19/env/Netherlands/Utrecht-94602-N/2020	Ø	Ø	Ø	Ø	Ø	C	T	Ø	G	Ø	Ø	Ø	Ø	Ø
hCoV-19/env/Netherlands/Wervershoof-93950-N/2020	C	G	A	G	G	T	G	Ø	Ø	Ø	Ø	Ø	Ø	Ø
hCoV-19/env/Netherlands/Wervershoof-92723-I/2020	C	G	A	Ø	G	Ø	Ø	Ø	G	Ø	Ø	Ø	A	Ø
hCoV-19/env/Netherlands/Wervershoof-92723-N/2020	C	G	A	G	G	T	G	C	G	C	C	G	A	G

Isolate	9269	9426	9477	9733	10097	10349	10525	10561	10819	10929	10933	11083
Wuhan-Hu-1 (MN908947)	G	C	T	C	G	C	C	T	T	C	T	G
hCoV-19/env/Netherlands/Amersfoort-92848-I/2020	Ø	Ø	Ø	Ø	G	C	C	T	T	C	T	G
hCoV-19/env/Netherlands/Amersfoort-92848-N/2020	G	C	T	C	G	C	C	T	T	C	T	G
hCoV-19/env/Netherlands/Amersfoort-93818-N/2020	G	Ø	Ø	C	Ø	Ø	Ø	Ø	T	C	T	G
hCoV-19/env/Netherlands/Amersfoort-92503-N/2020	G	Ø	A	C	G	C	C	T	T	C	T	G
hCoV-19/env/Netherlands/AmsterdamWest-92852-I/2020	G	Ø	Ø	C	G	C	C	T	T	C	T	G
hCoV-19/env/Netherlands/AmsterdamWest-92852-N1/2020	G	C	T	C	G	C	C	T	T	C	T	G
hCoV-19/env/Netherlands/AmsterdamWest-92852-N2/2020	G	C	T	C	G	C	C	T	T	C	T	G
hCoV-19/env/Netherlands/AmsterdamWest-94978-N/2020	Ø	Ø	Ø	Ø	Ø	Ø	Ø	Ø	Ø	Ø	Ø	Ø
hCoV-19/env/Netherlands/AmsterdamWest-93822-N/2020	G	C	T	C	Ø	C	C	T	T	C	T	G
hCoV-19/env/Netherlands/AmsterdamWest-95552-N/2020	Ø	Ø	Ø	Ø	Ø	Ø	Ø	Ø	Ø	Ø	Ø	Ø
hCoV-19/env/Netherlands/AmsterdamWest-95794-N/2020	Ø	Ø	Ø	Ø	Ø	Ø	Ø	Ø	Ø	Ø	Ø	Ø
hCoV-19/env/Netherlands/AmsterdamWest-94334-N/2020	G	C	T	C	G	C	C	T	Ø	Ø	Ø	Ø
hCoV-19/env/Netherlands/AmsterdamWest-92508-N/2020	G	C	T	C	G	C	C	T	T	C	T	G
hCoV-19/env/Netherlands/AmsterdamWest-94604-N/2020	Ø	T	T	Ø	Ø	Ø	C	T	T	Ø	Ø	Ø
hCoV-19/env/Netherlands/Apeldoorn-92502-I/2020	Ø	Ø	Ø	Ø	Ø	Ø	Ø	Ø	Ø	Ø	Ø	Ø
hCoV-19/env/Netherlands/Apeldoorn-92502-N/2020	G	Ø	Ø	C	G	C	C	T	T	C	T	G
hCoV-19/env/Belgium/Properinge-92949-I/2020	Ø	Ø	Ø	C	G	C	C	T	T	C	T	G
hCoV-19/env/Belgium/Properinge-92949-N/2020	G	C	T	Ø	A	Ø	Ø	Ø	T	C	T	G
hCoV-19/env/Belgium/Aartselaar-93030-I/2020	Ø	Ø	Ø	C	G	C	C	T	T	C	C	T
hCoV-19/env/Belgium/Aartselaar-93030-N/2020	G	C	T	C	G	C	C	T	T	C	T	G
hCoV-19/env/Belgium/Gent-93032-I/2020	Ø	Ø	Ø	Ø	Ø	Ø	C	T	T	Ø	Ø	G
hCoV-19/env/Belgium/Gent-93032-N/2020	G	C	T	Ø	G	Ø	C	T	T	Ø	Ø	G
hCoV-19/env/Belgium/Leuven-93034-I/2020	G	Ø	Ø	C	G	Ø	C	Ø	Ø	Ø	Ø	G
hCoV-19/env/Belgium/Leuven-93034-N/2020	G	Ø	Ø	C	Ø	C	C	T	T	C	T	G
hCoV-19/env/Belgium/Tienen-93036-I/2020	Ø	Ø	Ø	C	G	Ø	C	T	T	C	T	G
hCoV-19/env/Belgium/Tienen-93036-N/2020	G	C	T	C	G	C	C	T	T	C	T	G
hCoV-19/env/Belgium/Langemark-92943-N/2020	G	C	T	C	Ø	Ø	Ø	Ø	T	C	T	G
hCoV-19/env/Belgium/Lo-Reninge-92947-N/2020	G	C	T	Ø	Ø	C	C	T	T	Ø	Ø	G
hCoV-19/env/Netherlands/Beverwijk-92721-I/2020	Ø	Ø	Ø	Ø	G	Ø	Ø	Ø	Ø	Ø	Ø	G
hCoV-19/env/Netherlands/Beverwijk-92721-N/2020	G	C	T	C	G	C	C	T	T	C	T	G
hCoV-19/env/Netherlands/Delft-92965-I/2020	G	Ø	Ø	Ø	G	C	C	T	T	C	T	G
hCoV-19/env/Netherlands/Delft-92965-N/2020	T	C	T	C	G	T	C	T	T	C	T	G
hCoV-19/env/Netherlands/Delft-97044-N/2020	Ø	Ø	Ø	Ø	Ø	Ø	Ø	Ø	Ø	Ø	Ø	Ø
hCoV-19/env/Netherlands/Delft-94982-N/2020	Ø	Ø	Ø	Ø	Ø	Ø	Ø	Ø	Ø	Ø	Ø	Ø

Isolate	9269	9426	9477	9733	10097	10349	10525	10561	10819	10929	10933	11083
hCoV-19/env/Netherlands/Delft-93825-I/2020	Ø	Ø	Ø	C	G	Ø	C	C	T	C	T	G
hCoV-19/env/Netherlands/Delft-93825-N/2020	G	C	T	C	G	C	C	C	T	C	T	G
hCoV-19/env/Netherlands/Delft-94337-N/2020	Ø	Ø	Ø	Ø	G	Ø	Ø	Ø	Ø	Ø	Ø	Ø
hCoV-19/env/Netherlands/Delft-94607-N/2020	Ø	Ø	Ø	Ø	Ø	Ø	Ø	Ø	Ø	Ø	Ø	Ø
hCoV-19/env/Netherlands/Franeker-92719-I/2020	Ø	Ø	Ø	T	G	C	C	T	T	C	T	G
hCoV-19/env/Netherlands/Franeker-92719-N1/2020	G	C	T	T	G	C	C	T	T	C	T	G
hCoV-19/env/Netherlands/Franeker-92719-N2/2020	G	C	T	Ø	G	Ø	Ø	Ø	Ø	Ø	Ø	G
hCoV-19/env/Netherlands/HeeswijkDinther-93948-N/2020	Ø	Ø	Ø	Ø	Ø	Ø	C	T	T	Ø	Ø	Ø
hCoV-19/env/Netherlands/HeeswijkDinther-92499-N/2020	G	C	T	C	G	C	C	T	T	C	T	G
hCoV-19/env/Netherlands/Katwoude-92722-I/2020	Ø	Ø	Ø	Ø	G	C	C	T	T	C	T	G
hCoV-19/env/Netherlands/Katwoude-92722-N/2020	G	C	T	C	G	C	T	T	T	C	T	G
hCoV-19/env/Netherlands/Schiphol-92851-I/2020	Ø	Ø	Ø	C	G	Ø	C	T	T	C	T	G
hCoV-19/env/Netherlands/Schiphol-92851-N/2020	G	C	T	C	G	C	C	T	T	T	T	G
hCoV-19/env/Netherlands/Schiphol-96927-I/2020	Ø	Ø	Ø	Ø	Ø	Ø	Ø	Ø	C	C	T	G
hCoV-19/env/Netherlands/Schiphol-96927-N/2020	Ø	Ø	Ø	Ø	Ø	Ø	Ø	Ø	Ø	Ø	Ø	Ø
hCoV-19/env/Netherlands/Schiphol-93823-I/2020	Ø	Ø	Ø	Ø	G	C	C	T	T	C	T	G
hCoV-19/env/Netherlands/Schiphol-93823-N/2020	G	C	T	C	G	C	C	T	T	C	T	G
hCoV-19/env/Netherlands/Schiphol-94335-I/2020	Ø	Ø	Ø	C	G	C	C	T	T	C	T	T
hCoV-19/env/Netherlands/Schiphol-94335-N/2020	Ø	Ø	Ø	Ø	Ø	C	C	T	T	Ø	Ø	Ø
hCoV-19/env/Netherlands/Schiphol-92506-I/2020	Ø	Ø	Ø	C	G	C	C	T	T	C	T	G
hCoV-19/env/Netherlands/Schiphol-92506-N/2020	G	Ø	Ø	C	G	C	C	T	T	C	T	G
hCoV-19/env/Netherlands/Schiphol-94605-I/2020	Ø	Ø	Ø	Ø	G	C	C	T	T	C	T	G
hCoV-19/env/Netherlands/Schiphol-94605-N/2020	Ø	Ø	Ø	Ø	Ø	Ø	Ø	Ø	Ø	Ø	Ø	Ø
hCoV-19/env/Netherlands/Tilburg-92853-I/2020	G	Ø	Ø	C	G	C	Ø	T	T	C	T	G
hCoV-19/env/Netherlands/Tilburg-92853-N/2020	G	C	T	C	G	C	C	T	T	C	T	G
hCoV-19/env/Netherlands/Tilburg-93828-N/2020	Ø	Ø	Ø	C	Ø	Ø	C	T	T	Ø	Ø	Ø
hCoV-19/env/Netherlands/Tilburg-94339-I/2020	Ø	Ø	Ø	C	G	C	C	T	T	C	T	G
hCoV-19/env/Netherlands/Tilburg-94339-N/2020	Ø	Ø	Ø	Ø	Ø	Ø	Ø	Ø	Ø	Ø	Ø	Ø
hCoV-19/env/Netherlands/Tilburg-92509-I/2020	Ø	Ø	Ø	C	G	Ø	C	T	T	C	T	G
hCoV-19/env/Netherlands/Tilburg-92509-N/2020	G	C	T	C	Ø	C	C	T	T	C	T	G
hCoV-19/env/Netherlands/UtrechtOvervecht-92505-N/2020	G	C	T	C	G	C	C	T	T	C	T	G
hCoV-19/env/Netherlands/Utrecht-92849-I/2020	G	Ø	Ø	Ø	Ø	Ø	C	T	T	C	T	G
hCoV-19/env/Netherlands/Utrecht-92849-N/2020	Ø	Ø	Ø	Ø	G	C	C	T	T	Ø	Ø	G
hCoV-19/env/Netherlands/Utrecht-94976-N/2020	Ø	Ø	Ø	Ø	Ø	Ø	Ø	Ø	Ø	Ø	Ø	Ø
hCoV-19/env/Netherlands/Utrecht-93820-N/2020	G	Ø	Ø	C	G	Ø	C	T	T	C	T	T
hCoV-19/env/Netherlands/Utrecht-95550-N/2020	Ø	Ø	Ø	Ø	Ø	Ø	Ø	Ø	Ø	Ø	Ø	Ø
hCoV-19/env/Netherlands/Utrecht-94330-N/2020	Ø	Ø	Ø	Ø	G	Ø	Ø	Ø	Ø	Ø	Ø	Ø
hCoV-19/env/Netherlands/Utrecht-94331-N/2020	G	C	T	Ø	Ø	C	C	T	Ø	Ø	Ø	Ø
hCoV-19/env/Netherlands/Utrecht-92504-N/2020	G	C	T	C	G	C	C	T	T	C	T	G
hCoV-19/env/Netherlands/Utrecht-94602-N/2020	Ø	Ø	Ø	Ø	Ø	Ø	Ø	Ø	Ø	Ø	Ø	Ø
hCoV-19/env/Netherlands/Wervershoof-93950-N/2020	G	C	T	C	G	C	C	T	T	C	T	G
hCoV-19/env/Netherlands/Wervershoof-92723-I/2020	Ø	Ø	Ø	Ø	Ø	Ø	C	T	T	Ø	Ø	Ø
hCoV-19/env/Netherlands/Wervershoof-92723-N/2020	G	C	T	C	G	C	C	T	T	C	T	G

Isolate	11484	11596	11627	11648	11839	12754	12970	13210	13214	13237	13242
Wuhan-Hu-1 (MN908947)	T	G	T	G	A	C	C	T	G	A	G
hCoV-19/env/Netherlands/Amersfoort-92848-I/2020	T	T	T	G	Ø	C	C	T	G	A	G

Isolate	11484	11596	11627	11648	11839	12754	12970	13210	13214	13237	13242
hCoV-19/env/Netherlands/Amersfoort-92848-N/2020	T	G	T	G	A	C	C	T	G	A	G
hCoV-19/env/Netherlands/Amersfoort-93818-N/2020	Ø	Ø	Ø	Ø	A	C	Ø	Ø	Ø	Ø	Ø
hCoV-19/env/Netherlands/Amersfoort-92503-N/2020	T	G	T	G	A	C	C	T	G	A	G
hCoV-19/env/Netherlands/AmsterdamWest-92852-I/2020	T	G	T	G	Ø	Ø	Ø	Ø	Ø	Ø	Ø
hCoV-19/env/Netherlands/AmsterdamWest-92852-N1/2020	T	G	T	G	A	C	C	T	G	A	G
hCoV-19/env/Netherlands/AmsterdamWest-92852-N2/2020	T	G	T	G	A	C	C	T	G	A	G
hCoV-19/env/Netherlands/AmsterdamWest-94978-N/2020	Ø	Ø	Ø	Ø	A	C	Ø	Ø	Ø	Ø	Ø
hCoV-19/env/Netherlands/AmsterdamWest-93822-N/2020	T	G	T	G	A	C	C	T	G	A	G
hCoV-19/env/Netherlands/AmsterdamWest-95552-N/2020	Ø	Ø	Ø	Ø	Ø	C	Ø	Ø	Ø	Ø	Ø
hCoV-19/env/Netherlands/AmsterdamWest-95794-N/2020	Ø	Ø	Ø	Ø	Ø	Ø	Ø	Ø	Ø	Ø	Ø
hCoV-19/env/Netherlands/AmsterdamWest-94334-N/2020	T	G	T	G	Ø	C	C	T	G	A	G
hCoV-19/env/Netherlands/AmsterdamWest-92508-N/2020	T	G	T	G	A	C	C	T	G	A	G
hCoV-19/env/Netherlands/AmsterdamWest-94604-N/2020	Ø	Ø	Ø	Ø	Ø	Ø	Ø	Ø	Ø	Ø	Ø
hCoV-19/env/Netherlands/Apeldoorn-92502-I/2020	T	G	T	G	Ø	C	C	Ø	Ø	Ø	Ø
hCoV-19/env/Netherlands/Apeldoorn-92502-N/2020	T	G	T	G	A	C	C	T	G	A	G
hCoV-19/env/Belgium/Properinge-92949-I/2020	T	G	T	G	A	C	C	T	G	A	G
hCoV-19/env/Belgium/Properinge-92949-N/2020	T	G	C	T	Ø	C	C	Ø	Ø	Ø	Ø
hCoV-19/env/Belgium/Aartselaar-93030-I/2020	T	G	T	G	A	C	C	T	G	A	G
hCoV-19/env/Belgium/Aartselaar-93030-N/2020	T	G	T	G	Ø	C	C	Ø	Ø	Ø	Ø
hCoV-19/env/Belgium/Gent-93032-I/2020	T	G	T	G	A	C	Ø	T	G	A	G
hCoV-19/env/Belgium/Gent-93032-N/2020	T	G	T	G	A	C	C	Ø	Ø	Ø	Ø
hCoV-19/env/Belgium/Leuven-93034-I/2020	T	G	T	G	Ø	C	C	Ø	Ø	Ø	Ø
hCoV-19/env/Belgium/Leuven-93034-N/2020	T	G	T	G	G	C	C	T	G	A	G
hCoV-19/env/Belgium/Tienen-93036-I/2020	T	G	T	G	A	C	Ø	T	G	A	G
hCoV-19/env/Belgium/Tienen-93036-N/2020	T	G	T	G	A	C	T	Ø	Ø	Ø	Ø
hCoV-19/env/Belgium/Langemark-92943-N/2020	T	G	T	G	Ø	C	Ø	Ø	Ø	Ø	Ø
hCoV-19/env/Belgium/Lo-Reninge-92947-N/2020	T	Ø	Ø	Ø	Ø	C	C	Ø	Ø	Ø	Ø
hCoV-19/env/Netherlands/Beverwijk-92721-I/2020	C	G	T	G	A	C	Ø	T	G	A	G
hCoV-19/env/Netherlands/Beverwijk-92721-N/2020	T	G	T	G	A	C	C	T	G	A	G
hCoV-19/env/Netherlands/Delft-92965-I/2020	T	G	T	G	A	C	C	T	G	A	G
hCoV-19/env/Netherlands/Delft-92965-N/2020	T	G	T	G	A	C	C	T	G	A	G
hCoV-19/env/Netherlands/Delft-97044-N/2020	Ø	Ø	Ø	Ø	Ø	Ø	Ø	Ø	Ø	Ø	Ø
hCoV-19/env/Netherlands/Delft-94982-N/2020	Ø	Ø	Ø	Ø	Ø	Ø	Ø	T	G	A	G
hCoV-19/env/Netherlands/Delft-93825-I/2020	T	G	T	G	A	C	C	T	G	A	G
hCoV-19/env/Netherlands/Delft-93825-N/2020	T	G	T	G	Ø	C	Ø	Ø	Ø	Ø	Ø
hCoV-19/env/Netherlands/Delft-94337-N/2020	Ø	Ø	Ø	Ø	Ø	C	Ø	T	G	A	G
hCoV-19/env/Netherlands/Delft-94607-N/2020	Ø	Ø	Ø	Ø	Ø	Ø	Ø	Ø	Ø	Ø	Ø
hCoV-19/env/Netherlands/Franeker-92719-I/2020	T	G	T	G	A	C	C	T	G	A	G

Isolate	11484	11596	11627	11648	11839	12754	12970	13210	13214	13237	13242
hCoV-19/env/Netherlands/Franeker-92719-N1/2020	T	G	T	G	A	C	C	T	G	A	G
hCoV-19/env/Netherlands/Franeker-92719-N2/2020	T	Ø	Ø	Ø	Ø	Ø	Ø	Ø	Ø	Ø	Ø
hCoV-19/env/Netherlands/HeeswijkDinther-93948-N/2020	Ø	Ø	Ø	Ø	G	Ø	Ø	Ø	Ø	Ø	Ø
hCoV-19/env/Netherlands/HeeswijkDinther-92499-N/2020	T	Ø	Ø	Ø	A	C	C	T	G	A	G
hCoV-19/env/Netherlands/Katwoude-92722-I/2020	T	G	T	G	A	C	C	Ø	Ø	Ø	Ø
hCoV-19/env/Netherlands/Katwoude-92722-N/2020	T	Ø	Ø	Ø	A	C	Ø	T	G	A	G
hCoV-19/env/Netherlands/Schiphol-92851-I/2020	T	G	T	G	A	C	C	T	G	A	G
hCoV-19/env/Netherlands/Schiphol-92851-N/2020	T	Ø	Ø	Ø	A	C	C	T	G	A	G
hCoV-19/env/Netherlands/Schiphol-96927-I/2020	T	G	T	G	A	C	Ø	T	G	A	G
hCoV-19/env/Netherlands/Schiphol-96927-N/2020	Ø	Ø	Ø	Ø	A	Ø	Ø	Ø	Ø	Ø	Ø
hCoV-19/env/Netherlands/Schiphol-93823-I/2020	T	G	T	G	A	C	C	T	G	A	G
hCoV-19/env/Netherlands/Schiphol-93823-N/2020	T	Ø	Ø	Ø	A	C	Ø	T	G	G	G
hCoV-19/env/Netherlands/Schiphol-94335-I/2020	T	G	T	G	A	C	C	T	G	A	G
hCoV-19/env/Netherlands/Schiphol-94335-N/2020	Ø	Ø	Ø	Ø	A	C	C	Ø	Ø	Ø	Ø
hCoV-19/env/Netherlands/Schiphol-92506-I/2020	T	G	T	G	A	C	C	T	G	A	G
hCoV-19/env/Netherlands/Schiphol-92506-N/2020	T	G	T	G	A	C	C	T	G	A	G
hCoV-19/env/Netherlands/Schiphol-94605-I/2020	T	G	T	G	Ø	C	Ø	T	G	A	G
hCoV-19/env/Netherlands/Schiphol-94605-N/2020	Ø	Ø	Ø	Ø	A	Ø	Ø	Ø	Ø	Ø	Ø
hCoV-19/env/Netherlands/Tilburg-92853-I/2020	T	G	T	G	A	C	C	T	G	A	G
hCoV-19/env/Netherlands/Tilburg-92853-N/2020	T	G	T	G	Ø	C	C	Ø	Ø	Ø	Ø
hCoV-19/env/Netherlands/Tilburg-93828-N/2020	Ø	Ø	Ø	Ø	A	Ø	Ø	T	G	A	G
hCoV-19/env/Netherlands/Tilburg-94339-I/2020	T	G	T	G	A	C	C	T	G	A	G
hCoV-19/env/Netherlands/Tilburg-94339-N/2020	Ø	Ø	Ø	Ø	Ø	Ø	Ø	Ø	Ø	Ø	Ø
hCoV-19/env/Netherlands/Tilburg-92509-I/2020	T	G	T	G	A	Ø	C	T	G	A	G
hCoV-19/env/Netherlands/Tilburg-92509-N/2020	Ø	Ø	Ø	Ø	A	C	C	T	G	A	T
hCoV-19/env/Netherlands/UtrechtOvervecht-92505-N/2020	T	G	T	G	A	C	C	T	G	A	G
hCoV-19/env/Netherlands/Utrecht-92849-I/2020	T	G	T	G	Ø	C	C	T	G	A	G
hCoV-19/env/Netherlands/Utrecht-92849-N/2020	T	Ø	Ø	Ø	A	C	Ø	Ø	Ø	Ø	Ø
hCoV-19/env/Netherlands/Utrecht-94976-N/2020	Ø	Ø	Ø	Ø	Ø	Ø	Ø	Ø	Ø	Ø	Ø
hCoV-19/env/Netherlands/Utrecht-93820-N/2020	T	G	T	G	A	C	C	Ø	Ø	Ø	Ø
hCoV-19/env/Netherlands/Utrecht-95550-N/2020	Ø	Ø	Ø	Ø	Ø	Ø	Ø	Ø	Ø	Ø	Ø
hCoV-19/env/Netherlands/Utrecht-94330-N/2020	Ø	Ø	Ø	Ø	Ø	T	C	T	G	A	G
hCoV-19/env/Netherlands/Utrecht-94331-N/2020	Ø	Ø	Ø	Ø	A	C	Ø	T	G	A	G
hCoV-19/env/Netherlands/Utrecht-92504-N/2020	T	G	T	G	A	C	C	A	T	A	G
hCoV-19/env/Netherlands/Utrecht-94602-N/2020	Ø	Ø	Ø	Ø	Ø	C	Ø	T	G	A	G
hCoV-19/env/Netherlands/Wervershoof-93950-N/2020	T	G	T	G	A	C	C	Ø	Ø	Ø	Ø
hCoV-19/env/Netherlands/Wervershoof-92723-I/2020	T	G	T	G	Ø	C	C	T	G	A	G
hCoV-19/env/Netherlands/Wervershoof-92723-N/2020	T	G	T	G	A	C	C	Ø	Ø	Ø	Ø

Isolate	13374	13536	13779	13972	14408	14618	15324	15495	15939	16017	16061
Wuhan-Hu-1 (MN908947)	G	C	T	T	C	C	C	C	T	C	A
hCoV-19/env/Netherlands/Amersfoort-92848-I/2020	G	Ø	T	T	Ø	C	C	C	T	C	A
hCoV-19/env/Netherlands/Amersfoort-92848-N/2020	G	C	T	T	T	C	C	C	T	C	A
hCoV-19/env/Netherlands/Amersfoort-93818-N/2020	Ø	Ø	T	T	Ø	Ø	C	C	Ø	Ø	Ø
hCoV-19/env/Netherlands/Amersfoort-92503-N/2020	G	C	T	T	T	C	C	C	T	C	C
hCoV-19/env/Netherlands/AmsterdamWest-92852-I/2020	G	C	T	Ø	Ø	Ø	C	Ø	T	C	A
hCoV-19/env/Netherlands/AmsterdamWest-92852-N1/2020	G	C	T	T	T	C	C	C	T	C	A
hCoV-19/env/Netherlands/AmsterdamWest-92852-N2/2020	G	C	T	T	T	C	C	C	T	T	A
hCoV-19/env/Netherlands/AmsterdamWest-94978-N/2020	Ø	Ø	Ø	Ø	Ø	Ø	Ø	Ø	Ø	Ø	Ø
hCoV-19/env/Netherlands/AmsterdamWest-93822-N/2020	G	Ø	Ø	Ø	Ø	C	C	C	T	C	A
hCoV-19/env/Netherlands/AmsterdamWest-95552-N/2020	Ø	Ø	Ø	Ø	Ø	C	Ø	Ø	Ø	Ø	Ø
hCoV-19/env/Netherlands/AmsterdamWest-95794-N/2020	Ø	Ø	Ø	Ø	Ø	Ø	C	C	T	C	A
hCoV-19/env/Netherlands/AmsterdamWest-94334-N/2020	G	C	T	Ø	Ø	Ø	C	C	T	C	A
hCoV-19/env/Netherlands/AmsterdamWest-92508-N/2020	G	C	T	T	T	C	C	C	T	C	A
hCoV-19/env/Netherlands/AmsterdamWest-94604-N/2020	Ø	Ø	T	T	Ø	Ø	Ø	Ø	Ø	Ø	Ø
hCoV-19/env/Netherlands/Apeldoorn-92502-I/2020	Ø	Ø	Ø	Ø	Ø	Ø	C	Ø	Ø	Ø	Ø
hCoV-19/env/Netherlands/Apeldoorn-92502-N/2020	G	C	T	Ø	Ø	Ø	C	A	T	C	A
hCoV-19/env/Belgium/Properinge-92949-I/2020	G	Ø	T	T	Ø	C	C	C	T	C	A
hCoV-19/env/Belgium/Properinge-92949-N/2020	Ø	Ø	T	T	Ø	T	T	C	T	C	A
hCoV-19/env/Belgium/Aartselaar-93030-I/2020	G	Ø	T	Ø	Ø	C	C	C	T	C	A
hCoV-19/env/Belgium/Aartselaar-93030-N/2020	Ø	Ø	T	T	T	C	C	C	T	C	A
hCoV-19/env/Belgium/Gent-93032-I/2020	G	Ø	T	T	Ø	C	C	C	T	C	A
hCoV-19/env/Belgium/Gent-93032-N/2020	Ø	Ø	Ø	Ø	Ø	Ø	C	C	T	C	A
hCoV-19/env/Belgium/Leuven-93034-I/2020	G	Ø	Ø	Ø	Ø	C	C	C	T	C	A
hCoV-19/env/Belgium/Leuven-93034-N/2020	G	Ø	T	T	T	Ø	C	C	T	C	A
hCoV-19/env/Belgium/Tienen-93036-I/2020	G	Ø	Ø	Ø	Ø	C	C	C	T	C	A
hCoV-19/env/Belgium/Tienen-93036-N/2020	Ø	Ø	T	T	C	C	Ø	C	T	C	A
hCoV-19/env/Belgium/Langemark-92943-N/2020	Ø	Ø	Ø	Ø	Ø	Ø	C	C	T	C	A
hCoV-19/env/Belgium/Lo-Reninge-92947-N/2020	G	C	T	Ø	T	Ø	Ø	C	T	C	A
hCoV-19/env/Netherlands/Beverwijk-92721-I/2020	G	C	T	T	Ø	Ø	Ø	C	T	C	A
hCoV-19/env/Netherlands/Beverwijk-92721-N/2020	G	Ø	T	T	T	C	C	C	T	C	A
hCoV-19/env/Netherlands/Delft-92965-I/2020	G	Ø	Ø	T	Ø	C	C	C	C	C	A
hCoV-19/env/Netherlands/Delft-92965-N/2020	G	Ø	T	T	T	C	C	C	T	C	A
hCoV-19/env/Netherlands/Delft-97044-N/2020	Ø	Ø	Ø	Ø	Ø	Ø	Ø	Ø	Ø	Ø	Ø
hCoV-19/env/Netherlands/Delft-94982-N/2020	T	Ø	T	T	Ø	C	C	C	Ø	Ø	Ø
hCoV-19/env/Netherlands/Delft-93825-I/2020	G	Ø	T	T	Ø	C	C	C	T	C	A
hCoV-19/env/Netherlands/Delft-93825-N/2020	Ø	Ø	T	T	T	C	C	C	T	C	A
hCoV-19/env/Netherlands/Delft-94337-N/2020	G	Ø	C	T	Ø	C	Ø	Ø	Ø	Ø	Ø
hCoV-19/env/Netherlands/Delft-94607-N/2020	Ø	Ø	Ø	Ø	Ø	C	Ø	Ø	Ø	Ø	Ø
hCoV-19/env/Netherlands/Franeker-92719-I/2020	G	Ø	T	T	Ø	C	C	C	T	C	A
hCoV-19/env/Netherlands/Franeker-92719-N1/2020	G	C	T	G	T	C	C	C	T	C	A
hCoV-19/env/Netherlands/Franeker-92719-N2/2020	Ø	Ø	Ø	Ø	Ø	C	C	C	Ø	Ø	Ø
hCoV-19/env/Netherlands/HeeswijkDinther-93948-N/2020	Ø	Ø	Ø	Ø	Ø	Ø	C	C	Ø	Ø	Ø
hCoV-19/env/Netherlands/HeeswijkDinther-92499-N/2020	G	C	T	T	C	C	C	C	T	C	A
hCoV-19/env/Netherlands/Katwoude-92722-I/2020	Ø	Ø	Ø	Ø	Ø	Ø	Ø	C	Ø	Ø	Ø
hCoV-19/env/Netherlands/Katwoude-92722-N/2020	G	C	T	T	T	C	C	C	T	C	A

Isolate	13374	13536	13779	13972	14408	14618	15324	15495	15939	16017	16061
hCoV-19/env/Netherlands/Schiphol-92851-I/2020	G	Ø	Ø	Ø	Ø	C	C	Ø	T	Ø	A
hCoV-19/env/Netherlands/Schiphol-92851-N/2020	G	C	T	T	Ø	C	C	C	T	C	A
hCoV-19/env/Netherlands/Schiphol-96927-I/2020	G	Ø	Ø	Ø	Ø	C	C	C	T	Ø	Ø
hCoV-19/env/Netherlands/Schiphol-96927-N/2020	Ø	Ø	Ø	Ø	Ø	C	C	C	T	C	A
hCoV-19/env/Netherlands/Schiphol-93823-I/2020	G	Ø	Ø	Ø	Ø	C	C	C	T	Ø	Ø
hCoV-19/env/Netherlands/Schiphol-93823-N/2020	G	Ø	Ø	Ø	T	Ø	Ø	C	Ø	Ø	Ø
hCoV-19/env/Netherlands/Schiphol-94335-I/2020	G	Ø	Ø	Ø	Ø	C	C	C	T	C	A
hCoV-19/env/Netherlands/Schiphol-94335-N/2020	Ø	Ø	Ø	Ø	Ø	Ø	Ø	C	T	C	A
hCoV-19/env/Netherlands/Schiphol-92506-I/2020	G	Ø	Ø	Ø	Ø	C	C	C	T	C	A
hCoV-19/env/Netherlands/Schiphol-92506-N/2020	G	C	T	T	C	Ø	C	C	T	C	A
hCoV-19/env/Netherlands/Schiphol-94605-I/2020	G	Ø	Ø	Ø	Ø	C	C	C	T	C	A
hCoV-19/env/Netherlands/Schiphol-94605-N/2020	Ø	Ø	Ø	Ø	Ø	C	C	C	T	C	A
hCoV-19/env/Netherlands/Tilburg-92853-I/2020	G	Ø	Ø	T	C	C	C	C	T	C	A
hCoV-19/env/Netherlands/Tilburg-92853-N/2020	G	T	T	T	C	C	C	C	T	C	A
hCoV-19/env/Netherlands/Tilburg-93828-N/2020	G	Ø	T	T	Ø	Ø	C	C	T	C	A
hCoV-19/env/Netherlands/Tilburg-94339-I/2020	G	Ø	T	Ø	Ø	C	C	C	T	C	A
hCoV-19/env/Netherlands/Tilburg-94339-N/2020	Ø	Ø	T	T	Ø	C	C	C	T	C	A
hCoV-19/env/Netherlands/Tilburg-92509-I/2020	G	Ø	T	Ø	Ø	C	C	Ø	T	C	A
hCoV-19/env/Netherlands/Tilburg-92509-N/2020	G	C	T	T	C	C	C	C	T	C	A
hCoV-19/env/Netherlands/UtrechtOvervecht-92505-N/2020	G	C	T	T	T	C	C	C	T	C	A
hCoV-19/env/Netherlands/Utrecht-92849-I/2020	G	Ø	Ø	Ø	Ø	C	C	C	T	C	A
hCoV-19/env/Netherlands/Utrecht-92849-N/2020	Ø	Ø	T	T	Ø	C	C	C	T	C	A
hCoV-19/env/Netherlands/Utrecht-94976-N/2020	Ø	Ø	Ø	Ø	Ø	Ø	Ø	Ø	Ø	Ø	Ø
hCoV-19/env/Netherlands/Utrecht-93820-N/2020	G	C	T	Ø	T	Ø	C	C	Ø	Ø	Ø
hCoV-19/env/Netherlands/Utrecht-95550-N/2020	Ø	Ø	Ø	Ø	Ø	Ø	Ø	Ø	Ø	Ø	Ø
hCoV-19/env/Netherlands/Utrecht-94330-N/2020	G	Ø	T	T	T	C	C	C	T	C	A
hCoV-19/env/Netherlands/Utrecht-94331-N/2020	G	Ø	T	T	Ø	C	C	C	T	C	A
hCoV-19/env/Netherlands/Utrecht-92504-N/2020	G	C	T	T	T	C	C	C	T	C	A
hCoV-19/env/Netherlands/Utrecht-94602-N/2020	G	Ø	Ø	Ø	T	Ø	Ø	Ø	T	C	A
hCoV-19/env/Netherlands/Wervershoof-93950-N/2020	Ø	Ø	Ø	Ø	T	C	C	C	T	C	A
hCoV-19/env/Netherlands/Wervershoof-92723-I/2020	G	Ø	Ø	Ø	Ø	Ø	C	C	Ø	Ø	Ø
hCoV-19/env/Netherlands/Wervershoof-92723-N/2020	G	C	T	Ø	T	C	C	C	T	T	A

Isolate	16468	16538	16690	17129	17212	17410	17504	17822	17827	18029	18484	18838
Wuhan-Hu-1 (MN908947)	C	ATA	G	A	C	C	A	C	C	C	C	G
hCoV-19/env/Netherlands/Amersfoort-92848-I/2020	C	ATA	G	A	Ø	C	A	Ø	Ø	Ø	Ø	Ø
hCoV-19/env/Netherlands/Amersfoort-92848-N/2020	C	ATA	G	A	C	C	A	C	C	C	C	G
hCoV-19/env/Netherlands/Amersfoort-93818-N/2020	C	ATA	Ø	A	Ø	Ø	Ø	Ø	Ø	Ø	C	G
hCoV-19/env/Netherlands/Amersfoort-92503-N/2020	C	ATA	G	A	C	C	A	Ø	Ø	Ø	C	G
hCoV-19/env/Netherlands/AmsterdamWest-92852-I/2020	C	ATA	G	A	C	C	A	Ø	Ø	Ø	C	G
hCoV-19/env/Netherlands/AmsterdamWest-92852-N1/2020	C	ATA	G	A	C	C	A	C	C	C	C	G
hCoV-19/env/Netherlands/AmsterdamWest-92852-N2/2020	C	ATA	G	A	C	C	A	C	C	C	C	G
hCoV-19/env/Netherlands/AmsterdamWest-94978-N/2020	C	ATA	G	A	C	C	A	C	C	C	Ø	Ø
hCoV-19/env/Netherlands/AmsterdamWest-93822-N/2020	C	ATA	G	A	C	C	A	C	C	C	C	G
hCoV-19/env/Netherlands/AmsterdamWest-95552-N/2020	C	ATA	G	A	C	C	A	Ø	Ø	Ø	C	G
hCoV-19/env/Netherlands/AmsterdamWest-95794-N/2020	Ø	Ø	Ø	A	C	C	A	Ø	Ø	Ø	Ø	Ø
hCoV-19/env/Netherlands/AmsterdamWest-94334-N/2020	C	ATA	G	Ø	Ø	Ø	Ø	T	C	C	Ø	G

Isolate	16468	16538	16690	17129	17212	17410	17504	17822	17827	18029	18484	18838
hCoV-19/env/Netherlands/AmsterdamWest-92508-N/2020	C	ATA	G	A	C	C	A	C	C	C	C	G
hCoV-19/env/Netherlands/AmsterdamWest-94604-N/2020	T	ATA	G	Ø	Ø	Ø	Ø	Ø	Ø	Ø	Ø	Ø
hCoV-19/env/Netherlands/Apeldoorn-92502-I/2020	Ø	Ø	Ø	Ø	Ø	Ø	Ø	Ø	Ø	Ø	Ø	Ø
hCoV-19/env/Netherlands/Apeldoorn-92502-N/2020	C	ATA	G	A	C	C	A	Ø	Ø	Ø	C	Ø
hCoV-19/env/Belgium/Properinge-92949-I/2020	C	ATA	G	A	Ø	Ø	Ø	Ø	Ø	Ø	C	G
hCoV-19/env/Belgium/Properinge-92949-N/2020	C	ATA	G	A	C	C	A	Ø	Ø	Ø	C	G
hCoV-19/env/Belgium/Aartselaar-93030-I/2020	C	ATA	G	A	C	Ø	Ø	Ø	Ø	Ø	C	G
hCoV-19/env/Belgium/Aartselaar-93030-N/2020	C	ATA	G	A	Ø	Ø	Ø	C	C	C	C	G
hCoV-19/env/Belgium/Gent-93032-I/2020	C	---	G	Ø	C	Ø	Ø	Ø	Ø	Ø	C	Ø
hCoV-19/env/Belgium/Gent-93032-N/2020	C	ATA	G	A	C	C	A	Ø	Ø	Ø	C	G
hCoV-19/env/Belgium/Leuven-93034-I/2020	C	ATA	G	A	Ø	Ø	Ø	Ø	Ø	Ø	C	G
hCoV-19/env/Belgium/Leuven-93034-N/2020	C	ATA	G	A	C	C	A	C	C	C	C	G
hCoV-19/env/Belgium/Tienen-93036-I/2020	C	ATA	G	A	Ø	Ø	Ø	Ø	Ø	Ø	C	Ø
hCoV-19/env/Belgium/Tienen-93036-N/2020	C	ATA	G	A	C	C	A	Ø	Ø	Ø	C	G
hCoV-19/env/Belgium/Langemark-92943-N/2020	C	ATA	G	A	Ø	Ø	Ø	Ø	Ø	Ø	C	G
hCoV-19/env/Belgium/Lo-Reninge-92947-N/2020	C	ATA	G	A	C	C	A	C	C	C	C	G
hCoV-19/env/Netherlands/Beverwijk-92721-I/2020	C	ATA	G	Ø	Ø	Ø	Ø	Ø	Ø	Ø	C	G
hCoV-19/env/Netherlands/Beverwijk-92721-N/2020	C	ATA	G	A	C	C	A	C	C	C	C	G
hCoV-19/env/Netherlands/Delft-92965-I/2020	C	ATA	G	A	Ø	C	A	Ø	Ø	Ø	C	G
hCoV-19/env/Netherlands/Delft-92965-N/2020	C	ATA	G	A	C	C	A	C	T	C	C	G
hCoV-19/env/Netherlands/Delft-97044-N/2020	Ø	Ø	Ø	Ø	Ø	Ø	Ø	C	C	C	C	G
hCoV-19/env/Netherlands/Delft-94982-N/2020	Ø	Ø	Ø	Ø	Ø	Ø	Ø	C	C	C	C	G
hCoV-19/env/Netherlands/Delft-93825-I/2020	C	ATA	G	Ø	Ø	Ø	Ø	Ø	Ø	Ø	T	G
hCoV-19/env/Netherlands/Delft-93825-N/2020	C	ATA	G	A	A	C	A	C	C	Ø	C	G
hCoV-19/env/Netherlands/Delft-94337-N/2020	C	ATA	G	A	C	C	A	Ø	Ø	Ø	C	G
hCoV-19/env/Netherlands/Delft-94607-N/2020	C	ATA	G	A	C	C	A	Ø	Ø	Ø	C	G
hCoV-19/env/Netherlands/Franeker-92719-I/2020	C	ATA	G	A	C	C	A	Ø	Ø	Ø	C	G
hCoV-19/env/Netherlands/Franeker-92719-N1/2020	C	ATA	G	A	C	C	A	C	C	C	C	G
hCoV-19/env/Netherlands/Franeker-92719-N2/2020	C	ATA	G	Ø	Ø	Ø	Ø	Ø	Ø	Ø	Ø	Ø
hCoV-19/env/Netherlands/HeeswijkDinther-93948-N/2020	C	ATA	G	A	Ø	Ø	Ø	Ø	Ø	Ø	Ø	Ø
hCoV-19/env/Netherlands/HeeswijkDinther-92499-N/2020	C	ATA	G	A	C	C	A	C	C	C	C	G
hCoV-19/env/Netherlands/Katwoude-92722-I/2020	C	ATA	T	A	Ø	Ø	Ø	Ø	Ø	Ø	C	G
hCoV-19/env/Netherlands/Katwoude-92722-N/2020	C	ATA	G	A	C	C	A	C	C	C	C	G
hCoV-19/env/Netherlands/Schiphol-92851-I/2020	C	ATA	G	A	Ø	Ø	Ø	Ø	Ø	Ø	C	Ø
hCoV-19/env/Netherlands/Schiphol-92851-N/2020	C	ATA	G	A	Ø	Ø	A	C	C	C	C	G
hCoV-19/env/Netherlands/Schiphol-96927-I/2020	C	ATA	Ø	Ø	Ø	Ø	Ø	Ø	Ø	Ø	Ø	Ø
hCoV-19/env/Netherlands/Schiphol-96927-N/2020	C	ATA	G	A	C	C	A	Ø	Ø	Ø	Ø	Ø
hCoV-19/env/Netherlands/Schiphol-93823-I/2020	C	ATA	G	A	Ø	Ø	Ø	Ø	Ø	Ø	Ø	Ø
hCoV-19/env/Netherlands/Schiphol-93823-N/2020	C	ATA	G	A	C	C	A	Ø	Ø	Ø	Ø	Ø
hCoV-19/env/Netherlands/Schiphol-94335-I/2020	C	ATA	G	G	Ø	Ø	Ø	C	C	C	C	G
hCoV-19/env/Netherlands/Schiphol-94335-N/2020	C	ATA	G	Ø	Ø	Ø	Ø	C	C	C	Ø	G
hCoV-19/env/Netherlands/Schiphol-92506-I/2020	C	ATA	G	A	Ø	Ø	Ø	Ø	Ø	Ø	Ø	Ø
hCoV-19/env/Netherlands/Schiphol-92506-N/2020	C	ATA	G	A	C	C	A	C	C	C	C	G
hCoV-19/env/Netherlands/Schiphol-94605-I/2020	C	ATA	G	A	Ø	Ø	Ø	Ø	Ø	Ø	C	G
hCoV-19/env/Netherlands/Schiphol-94605-N/2020	Ø	Ø	Ø	A	C	C	A	Ø	Ø	Ø	Ø	Ø
hCoV-19/env/Netherlands/Tilburg-92853-I/2020	C	ATA	G	A	Ø	Ø	Ø	Ø	Ø	Ø	C	G
hCoV-19/env/Netherlands/Tilburg-92853-N/2020	C	ATA	G	A	C	C	A	C	C	C	C	G
hCoV-19/env/Netherlands/Tilburg-93828-N/2020	Ø	Ø	Ø	A	Ø	Ø	Ø	C	C	C	C	G
hCoV-19/env/Netherlands/Tilburg-94339-I/2020	C	ATA	G	Ø	Ø	C	Ø	Ø	Ø	Ø	C	G

Isolate	16468	16538	16690	17129	17212	17410	17504	17822	17827	18029	18484	18838
hCoV-19/env/Netherlands/Tilburg-94339-N/2020	C	ATA	G	A	C	C	A	Ø	Ø	Ø	Ø	Ø
hCoV-19/env/Netherlands/Tilburg-92509-I/2020	Ø	ATA	G	Ø	Ø	Ø	Ø	Ø	Ø	Ø	Ø	Ø
hCoV-19/env/Netherlands/Tilburg-92509-N/2020	C	ATA	G	A	C	T	A	C	C	C	C	T
hCoV-19/env/Netherlands/UtrechtOvervecht-92505-N/2020	C	ATA	G	A	C	C	A	C	C	T	C	G
hCoV-19/env/Netherlands/Utrecht-92849-I/2020	C	ATA	G	A	Ø	Ø	Ø	Ø	Ø	Ø	C	G
hCoV-19/env/Netherlands/Utrecht-92849-N/2020	C	ATA	G	A	C	C	A	C	C	C	C	G
hCoV-19/env/Netherlands/Utrecht-94976-N/2020	Ø	Ø	Ø	Ø	Ø	Ø	Ø	Ø	Ø	Ø	C	G
hCoV-19/env/Netherlands/Utrecht-93820-N/2020	C	ATA	G	A	C	C	A	Ø	Ø	Ø	C	G
hCoV-19/env/Netherlands/Utrecht-95550-N/2020	Ø	Ø	Ø	Ø	Ø	Ø	Ø	Ø	Ø	Ø	Ø	Ø
hCoV-19/env/Netherlands/Utrecht-94330-N/2020	C	ATA	G	Ø	Ø	Ø	Ø	C	C	C	C	G
hCoV-19/env/Netherlands/Utrecht-94331-N/2020	C	ATA	G	A	C	C	A	Ø	Ø	Ø	C	G
hCoV-19/env/Netherlands/Utrecht-92504-N/2020	C	ATA	G	A	C	C	A	C	C	C	C	G
hCoV-19/env/Netherlands/Utrecht-94602-N/2020	C	ATA	G	Ø	Ø	Ø	Ø	Ø	Ø	Ø	Ø	Ø
hCoV-19/env/Netherlands/Wervershoof-93950-N/2020	C	ATA	G	A	C	C	A	C	C	C	C	G
hCoV-19/env/Netherlands/Wervershoof-92723-I/2020	Ø	Ø	Ø	A	C	C	G	Ø	Ø	Ø	C	G
hCoV-19/env/Netherlands/Wervershoof-92723-N/2020	C	ATA	G	A	C	C	A	C	C	C	C	G

Isolate	18998	19072	19086	19170	19273	19480	19509	19586	19839	20087	20234	20268
Wuhan-Hu-1 (MN908947)	C	G	G	C	C	G	G	C	T	G	C	A
hCoV-19/env/Netherlands/Amersfoort-92848-I/2020	Ø	Ø	Ø	Ø	Ø	G	G	C	T	G	C	G
hCoV-19/env/Netherlands/Amersfoort-92848-N/2020	C	G	G	C	C	G	G	C	T	G	C	A
hCoV-19/env/Netherlands/Amersfoort-93818-N/2020	Ø	Ø	Ø	C	C	G	G	Ø	Ø	Ø	Ø	Ø
hCoV-19/env/Netherlands/Amersfoort-92503-N/2020	C	G	G	C	C	G	G	C	T	G	C	A
hCoV-19/env/Netherlands/AmsterdamWest-92852-I/2020	Ø	Ø	Ø	C	C	G	G	Ø	Ø	Ø	Ø	Ø
hCoV-19/env/Netherlands/AmsterdamWest-92852-N1/2020	C	G	G	C	C	G	G	C	T	G	C	A
hCoV-19/env/Netherlands/AmsterdamWest-92852-N2/2020	C	G	G	C	C	G	G	C	T	G	C	A
hCoV-19/env/Netherlands/AmsterdamWest-94978-N/2020	Ø	Ø	Ø	Ø	Ø	Ø	Ø	Ø	Ø	Ø	Ø	Ø
hCoV-19/env/Netherlands/AmsterdamWest-93822-N/2020	Ø	Ø	Ø	C	C	G	G	C	T	G	C	A
hCoV-19/env/Netherlands/AmsterdamWest-95552-N/2020	Ø	Ø	Ø	Ø	Ø	Ø	Ø	Ø	T	G	Ø	Ø
hCoV-19/env/Netherlands/AmsterdamWest-95794-N/2020	Ø	Ø	Ø	Ø	Ø	Ø	Ø	Ø	Ø	Ø	Ø	Ø
hCoV-19/env/Netherlands/AmsterdamWest-94334-N/2020	C	G	G	C	Ø	G	G	C	T	G	Ø	Ø
hCoV-19/env/Netherlands/AmsterdamWest-92508-N/2020	C	G	G	C	C	G	G	C	T	G	C	A
hCoV-19/env/Netherlands/AmsterdamWest-94604-N/2020	Ø	Ø	Ø	Ø	Ø	Ø	Ø	Ø	Ø	Ø	Ø	Ø
hCoV-19/env/Netherlands/Apeldoorn-92502-I/2020	Ø	Ø	Ø	Ø	Ø	Ø	Ø	Ø	Ø	Ø	Ø	Ø
hCoV-19/env/Netherlands/Apeldoorn-92502-N/2020	Ø	Ø	Ø	Ø	Ø	G	G	C	T	Ø	Ø	Ø
hCoV-19/env/Belgium/Properinge-92949-I/2020	Ø	Ø	Ø	Ø	C	Ø	Ø	C	Ø	Ø	C	A
hCoV-19/env/Belgium/Properinge-92949-N/2020	C	G	G	C	C	G	G	C	C	Ø	Ø	Ø
hCoV-19/env/Belgium/Aartselaar-93030-I/2020	Ø	Ø	Ø	Ø	Ø	G	G	C	T	G	C	A
hCoV-19/env/Belgium/Aartselaar-93030-N/2020	Ø	Ø	Ø	C	C	G	G	C	T	G	C	A
hCoV-19/env/Belgium/Gent-93032-I/2020	Ø	Ø	Ø	Ø	Ø	G	Ø	C	T	G	C	A
hCoV-19/env/Belgium/Gent-93032-N/2020	C	G	G	T	C	G	A	C	C	G	C	A
hCoV-19/env/Belgium/Leuven-93034-I/2020	Ø	Ø	Ø	Ø	Ø	Ø	Ø	Ø	Ø	G	C	A
hCoV-19/env/Belgium/Leuven-93034-N/2020	Ø	Ø	Ø	C	C	G	G	Ø	T	Ø	Ø	Ø
hCoV-19/env/Belgium/Tienen-93036-I/2020	Ø	Ø	Ø	Ø	Ø	Ø	Ø	C	Ø	Ø	C	A
hCoV-19/env/Belgium/Tienen-93036-N/2020	Ø	Ø	Ø	C	C	G	G	Ø	T	T	C	A
hCoV-19/env/Belgium/Langemark-92943-N/2020	C	T	G	C	C	G	G	C	C	G	Ø	Ø
hCoV-19/env/Belgium/Lo-Reninge-92947-N/2020	C	G	G	C	C	G	G	C	T	G	C	A

Isolate	18998	19072	19086	19170	19273	19480	19509	19586	19839	20087	20234	20268
hCoV-19/env/Netherlands/Beverwijk-92721-I/2020	Ø	Ø	Ø	Ø	Ø	Ø	Ø	Ø	Ø	G	C	Ø
hCoV-19/env/Netherlands/Beverwijk-92721-N/2020	C	G	G	C	C	G	G	C	T	G	C	A
hCoV-19/env/Netherlands/Delft-92965-I/2020	Ø	Ø	Ø	T	C	G	G	C	Ø	Ø	Ø	A
hCoV-19/env/Netherlands/Delft-92965-N/2020	C	G	G	C	C	G	G	C	T	G	C	A
hCoV-19/env/Netherlands/Delft-97044-N/2020	Ø	Ø	Ø	T	C	G	A	Ø	Ø	Ø	Ø	Ø
hCoV-19/env/Netherlands/Delft-94982-N/2020	Ø	Ø	Ø	C	C	G	G	Ø	Ø	Ø	Ø	Ø
hCoV-19/env/Netherlands/Delft-93825-I/2020	Ø	Ø	Ø	Ø	C	G	G	C	T	Ø	C	A
hCoV-19/env/Netherlands/Delft-93825-N/2020	C	G	G	C	Ø	G	G	C	T	Ø	Ø	Ø
hCoV-19/env/Netherlands/Delft-94337-N/2020	Ø	Ø	Ø	C	C	G	G	Ø	Ø	Ø	Ø	Ø
hCoV-19/env/Netherlands/Delft-94607-N/2020	Ø	Ø	Ø	Ø	Ø	Ø	Ø	Ø	Ø	Ø	Ø	Ø
hCoV-19/env/Netherlands/Franeker-92719-I/2020	C	G	G	C	C	G	G	C	Ø	Ø	C	A
hCoV-19/env/Netherlands/Franeker-92719-N1/2020	C	G	G	C	C	G	G	C	T	G	C	A
hCoV-19/env/Netherlands/Franeker-92719-N2/2020	Ø	Ø	Ø	C	C	G	G	Ø	T	G	Ø	Ø
hCoV-19/env/Netherlands/HeeswijkDinther-93948-N/2020	Ø	Ø	Ø	Ø	Ø	Ø	Ø	Ø	Ø	Ø	Ø	Ø
hCoV-19/env/Netherlands/HeeswijkDinther-92499-N/2020	C	G	G	C	C	G	G	T	T	G	C	A
hCoV-19/env/Netherlands/Katwoude-92722-I/2020	Ø	Ø	Ø	Ø	Ø	G	G	C	T	G	C	A
hCoV-19/env/Netherlands/Katwoude-92722-N/2020	C	G	G	C	C	G	G	C	T	G	C	A
hCoV-19/env/Netherlands/Schiphol-92851-I/2020	Ø	Ø	Ø	Ø	Ø	G	G	C	T	G	C	A
hCoV-19/env/Netherlands/Schiphol-92851-N/2020	Ø	Ø	Ø	C	C	G	G	Ø	T	G	C	A
hCoV-19/env/Netherlands/Schiphol-96927-I/2020	Ø	Ø	Ø	Ø	Ø	Ø	Ø	Ø	Ø	Ø	Ø	Ø
hCoV-19/env/Netherlands/Schiphol-96927-N/2020	Ø	Ø	Ø	C	C	G	G	Ø	Ø	Ø	Ø	Ø
hCoV-19/env/Netherlands/Schiphol-93823-I/2020	Ø	Ø	Ø	Ø	Ø	Ø	Ø	Ø	Ø	Ø	Ø	Ø
hCoV-19/env/Netherlands/Schiphol-93823-N/2020	Ø	Ø	Ø	C	C	G	G	Ø	T	G	C	A
hCoV-19/env/Netherlands/Schiphol-94335-I/2020	Ø	Ø	Ø	Ø	Ø	Ø	Ø	Ø	Ø	Ø	Ø	A
hCoV-19/env/Netherlands/Schiphol-94335-N/2020	C	G	G	C	Ø	G	G	C	T	G	Ø	Ø
hCoV-19/env/Netherlands/Schiphol-92506-I/2020	Ø	Ø	Ø	Ø	C	G	G	C	Ø	G	C	A
hCoV-19/env/Netherlands/Schiphol-92506-N/2020	T	G	G	C	C	G	G	C	T	G	C	A
hCoV-19/env/Netherlands/Schiphol-94605-I/2020	Ø	Ø	Ø	C	C	G	G	Ø	Ø	Ø	Ø	Ø
hCoV-19/env/Netherlands/Schiphol-94605-N/2020	Ø	Ø	Ø	Ø	Ø	Ø	Ø	Ø	Ø	Ø	Ø	Ø
hCoV-19/env/Netherlands/Tilburg-92853-I/2020	C	G	G	C	C	G	G	C	Ø	G	C	A
hCoV-19/env/Netherlands/Tilburg-92853-N/2020	C	G	G	C	C	G	G	C	T	G	C	A
hCoV-19/env/Netherlands/Tilburg-93828-N/2020	Ø	Ø	Ø	Ø	Ø	Ø	Ø	Ø	Ø	Ø	Ø	Ø
hCoV-19/env/Netherlands/Tilburg-94339-I/2020	C	G	G	C	C	G	G	C	T	Ø	C	A
hCoV-19/env/Netherlands/Tilburg-94339-N/2020	Ø	Ø	Ø	Ø	Ø	Ø	Ø	Ø	Ø	Ø	Ø	Ø
hCoV-19/env/Netherlands/Tilburg-92509-I/2020	Ø	Ø	Ø	Ø	Ø	Ø	G	Ø	Ø	Ø	C	A
hCoV-19/env/Netherlands/Tilburg-92509-N/2020	C	G	T	C	Ø	Ø	Ø	Ø	T	G	C	A
hCoV-19/env/Netherlands/UtrechtOvervecht-92505-N/2020	C	G	G	C	C	G	G	C	T	G	C	A
hCoV-19/env/Netherlands/Utrecht-92849-I/2020	Ø	Ø	Ø	Ø	Ø	Ø	Ø	Ø	Ø	G	C	A
hCoV-19/env/Netherlands/Utrecht-92849-N/2020	Ø	Ø	Ø	C	C	G	G	Ø	T	G	Ø	Ø
hCoV-19/env/Netherlands/Utrecht-94976-N/2020	Ø	Ø	Ø	C	C	G	G	Ø	Ø	Ø	Ø	Ø
hCoV-19/env/Netherlands/Utrecht-93820-N/2020	Ø	Ø	Ø	C	C	G	G	Ø	Ø	Ø	Ø	Ø
hCoV-19/env/Netherlands/Utrecht-95550-N/2020	Ø	Ø	Ø	Ø	Ø	Ø	Ø	Ø	Ø	Ø	Ø	Ø
hCoV-19/env/Netherlands/Utrecht-94330-N/2020	C	G	G	C	C	A	G	Ø	Ø	Ø	Ø	Ø
hCoV-19/env/Netherlands/Utrecht-94331-N/2020	Ø	Ø	Ø	C	C	G	G	Ø	T	G	Ø	Ø
hCoV-19/env/Netherlands/Utrecht-92504-N/2020	C	G	G	C	C	G	G	C	T	G	C	A
hCoV-19/env/Netherlands/Utrecht-94602-N/2020	Ø	Ø	Ø	Ø	Ø	Ø	Ø	Ø	Ø	Ø	Ø	Ø
hCoV-19/env/Netherlands/Wervershoof-93950-N/2020	C	G	G	C	T	G	G	Ø	T	G	Ø	Ø
hCoV-19/env/Netherlands/Wervershoof-92723-I/2020	C	Ø	Ø	C	C	G	G	Ø	Ø	G	T	A
hCoV-19/env/Netherlands/Wervershoof-92723-N/2020	C	G	G	C	C	G	G	C	T	G	C	A

Isolate	20900	21137	21140	21142	21207	23009	23403	23622	23877	24077	24095	24159
Wuhan-Hu-1 (MN908947)	G	A	T	G	C	G	A	T	T	G	G	C
hCoV-19/env/Netherlands/Amersfoort-92848-I/2020	G	A	T	G	Ø	G	R	Ø	Ø	Ø	Ø	Ø
hCoV-19/env/Netherlands/Amersfoort-92848-N/2020	G	A	T	G	C	G	G	C	T	G	G	C
hCoV-19/env/Netherlands/Amersfoort-93818-N/2020	Ø	A	T	G	C	Ø	A	Ø	Ø	Ø	Ø	Ø
hCoV-19/env/Netherlands/Amersfoort-92503-N/2020	G	A	T	G	Ø	G	A	T	T	G	G	C
hCoV-19/env/Netherlands/AmsterdamWest-92852-I/2020	Ø	Ø	Ø	Ø	Ø	G	G	Ø	Ø	Ø	Ø	C
hCoV-19/env/Netherlands/AmsterdamWest-92852-N1/2020	G	A	T	G	C	G	G	T	T	G	G	C
hCoV-19/env/Netherlands/AmsterdamWest-92852-N2/2020	G	A	T	G	C	G	G	T	T	G	G	C
hCoV-19/env/Netherlands/AmsterdamWest-94978-N/2020	Ø	A	T	G	C	Ø	Ø	Ø	Ø	Ø	Ø	Ø
hCoV-19/env/Netherlands/AmsterdamWest-93822-N/2020	G	A	T	G	C	G	G	T	Ø	G	G	C
hCoV-19/env/Netherlands/AmsterdamWest-95552-N/2020	Ø	A	T	G	C	G	G	Ø	Ø	Ø	Ø	Ø
hCoV-19/env/Netherlands/AmsterdamWest-95794-N/2020	Ø	Ø	Ø	Ø	Ø	Ø	Ø	Ø	Ø	Ø	Ø	Ø
hCoV-19/env/Netherlands/AmsterdamWest-94334-N/2020	G	A	T	G	C	G	G	Ø	Ø	G	T	C
hCoV-19/env/Netherlands/AmsterdamWest-92508-N/2020	G	A	T	G	Ø	G	G	T	T	G	G	C
hCoV-19/env/Netherlands/AmsterdamWest-94604-N/2020	Ø	A	T	G	C	Ø	Ø	Ø	Ø	Ø	Ø	Ø
hCoV-19/env/Netherlands/Apeldoorn-92502-I/2020	Ø	Ø	Ø	Ø	Ø	G	G	Ø	Ø	G	G	C
hCoV-19/env/Netherlands/Apeldoorn-92502-N/2020	G	A	T	G	C	G	A	Ø	T	G	G	C
hCoV-19/env/Belgium/Properinge-92949-I/2020	G	Ø	Ø	Ø	Ø	G	G	Ø	Ø	G	G	C
hCoV-19/env/Belgium/Properinge-92949-N/2020	G	A	T	G	Ø	G	G	Ø	Ø	Ø	Ø	Ø
hCoV-19/env/Belgium/Aartselaar-93030-I/2020	G	A	T	G	Ø	G	G	Ø	Ø	G	G	C
hCoV-19/env/Belgium/Aartselaar-93030-N/2020	G	A	T	G	C	G	G	T	T	G	G	C
hCoV-19/env/Belgium/Gent-93032-I/2020	G	A	T	G	Ø	G	G	Ø	Ø	Ø	Ø	Ø
hCoV-19/env/Belgium/Gent-93032-N/2020	G	A	T	G	C	Ø	Ø	Ø	T	G	G	C
hCoV-19/env/Belgium/Leuven-93034-I/2020	T	A	T	G	Ø	G	G	Ø	Ø	Ø	Ø	Ø
hCoV-19/env/Belgium/Leuven-93034-N/2020	G	A	T	G	Ø	G	G	Ø	Ø	G	G	C
hCoV-19/env/Belgium/Tienen-93036-I/2020	G	A	T	G	Ø	G	R	Ø	Ø	Ø	Ø	Ø
hCoV-19/env/Belgium/Tienen-93036-N/2020	G	A	T	G	C	G	G	T	T	G	G	C
hCoV-19/env/Belgium/Langemark-92943-N/2020	Ø	A	T	G	C	G	A	Ø	T	G	G	C
hCoV-19/env/Belgium/Lo-Reninge-92947-N/2020	G	A	T	G	C	T	G	T	T	G	G	C
hCoV-19/env/Netherlands/Beverwijk-92721-I/2020	G	A	T	G	Ø	G	G	T	Ø	G	G	C
hCoV-19/env/Netherlands/Beverwijk-92721-N/2020	G	A	T	G	C	G	G	T	T	G	G	C
hCoV-19/env/Netherlands/Delft-92965-I/2020	G	A	T	G	Ø	G	G	Ø	Ø	G	G	C
hCoV-19/env/Netherlands/Delft-92965-N/2020	G	A	T	G	C	G	G	T	T	G	G	C
hCoV-19/env/Netherlands/Delft-97044-N/2020	Ø	Ø	Ø	Ø	Ø	Ø	Ø	Ø	Ø	Ø	Ø	Ø
hCoV-19/env/Netherlands/Delft-94982-N/2020	Ø	Ø	Ø	Ø	Ø	Ø	Ø	Ø	Ø	Ø	Ø	Ø
hCoV-19/env/Netherlands/Delft-93825-I/2020	G	A	T	G	Ø	G	G	T	Ø	Ø	Ø	C
hCoV-19/env/Netherlands/Delft-93825-N/2020	G	A	T	G	C	G	G	T	Ø	Ø	Ø	Ø
hCoV-19/env/Netherlands/Delft-94337-N/2020	Ø	A	T	G	C	Ø	Ø	Ø	T	G	G	C
hCoV-19/env/Netherlands/Delft-94607-N/2020	Ø	Ø	Ø	Ø	Ø	G	G	Ø	T	G	G	C
hCoV-19/env/Netherlands/Franeker-92719-I/2020	G	A	T	G	C	G	G	Ø	Ø	G	G	C
hCoV-19/env/Netherlands/Franeker-92719-N1/2020	G	A	T	G	C	G	G	T	T	G	G	C
hCoV-19/env/Netherlands/Franeker-92719-N2/2020	Ø	A	T	G	C	G	G	Ø	T	G	G	C
hCoV-19/env/Netherlands/HeeswijkDinther-93948-N/2020	G	Ø	Ø	Ø	Ø	G	G	Ø	Ø	Ø	Ø	Ø
hCoV-19/env/Netherlands/HeeswijkDinther-92499-N/2020	G	A	T	G	C	G	G	T	T	G	G	C
hCoV-19/env/Netherlands/Katwoude-92722-I/2020	G	A	T	G	Ø	G	G	Ø	Ø	G	G	C
hCoV-19/env/Netherlands/Katwoude-92722-N/2020	G	A	T	G	C	G	G	Ø	Ø	G	G	C

Isolate	20900	21137	21140	21142	21207	23009	23403	23622	23877	24077	24095	24159
hCoV-19/env/Netherlands/Schiphol-92851-I/2020	G	A	T	G	Ø	G	G	T	Ø	G	G	C
hCoV-19/env/Netherlands/Schiphol-92851-N/2020	G	A	T	G	C	G	G	T	Ø	G	G	C
hCoV-19/env/Netherlands/Schiphol-96927-I/2020	Ø	Ø	Ø	Ø	Ø	Ø	Ø	Ø	Ø	Ø	Ø	Ø
hCoV-19/env/Netherlands/Schiphol-96927-N/2020	Ø	Ø	Ø	Ø	Ø	Ø	Ø	Ø	Ø	Ø	Ø	Ø
hCoV-19/env/Netherlands/Schiphol-93823-I/2020	G	A	T	G	Ø	G	G	Ø	Ø	Ø	Ø	C
hCoV-19/env/Netherlands/Schiphol-93823-N/2020	G	A	T	G	T	G	Ø	Ø	Ø	Ø	Ø	Ø
hCoV-19/env/Netherlands/Schiphol-94335-I/2020	G	A	T	G	Ø	Ø	Ø	Ø	Ø	Ø	Ø	C
hCoV-19/env/Netherlands/Schiphol-94335-N/2020	G	A	T	G	Ø	Ø	Ø	Ø	Ø	Ø	Ø	Ø
hCoV-19/env/Netherlands/Schiphol-92506-I/2020	G	A	C	T	Ø	G	G	Ø	Ø	G	G	C
hCoV-19/env/Netherlands/Schiphol-92506-N/2020	G	A	T	G	Ø	G	G	T	T	G	G	T
hCoV-19/env/Netherlands/Schiphol-94605-I/2020	G	Ø	Ø	Ø	Ø	G	G	Ø	Ø	G	G	C
hCoV-19/env/Netherlands/Schiphol-94605-N/2020	Ø	Ø	Ø	Ø	Ø	G	G	Ø	Ø	Ø	Ø	Ø
hCoV-19/env/Netherlands/Tilburg-92853-I/2020	G	A	T	G	Ø	G	G	Ø	Ø	G	G	C
hCoV-19/env/Netherlands/Tilburg-92853-N/2020	G	A	T	G	C	G	A	T	T	G	G	C
hCoV-19/env/Netherlands/Tilburg-93828-N/2020	Ø	A	T	G	C	Ø	Ø	Ø	Ø	Ø	Ø	Ø
hCoV-19/env/Netherlands/Tilburg-94339-I/2020	G	A	T	G	Ø	G	G	Ø	G	G	G	C
hCoV-19/env/Netherlands/Tilburg-94339-N/2020	Ø	Ø	Ø	Ø	Ø	Ø	Ø	Ø	Ø	Ø	Ø	Ø
hCoV-19/env/Netherlands/Tilburg-92509-I/2020	G	A	T	G	Ø	G	A	Ø	Ø	G	G	C
hCoV-19/env/Netherlands/Tilburg-92509-N/2020	G	A	T	G	Ø	G	G	T	Ø	Ø	Ø	Ø
hCoV-19/env/Netherlands/UtrechtOvervecht-92505-N/2020	Ø	A	T	G	C	G	A	T	T	G	G	C
hCoV-19/env/Netherlands/Utrecht-92849-I/2020	G	A	T	G	Ø	Ø	G	T	Ø	T	G	C
hCoV-19/env/Netherlands/Utrecht-92849-N/2020	Ø	A	T	G	C	G	G	Ø	Ø	Ø	Ø	Ø
hCoV-19/env/Netherlands/Utrecht-94976-N/2020	Ø	Ø	Ø	Ø	Ø	G	G	Ø	Ø	Ø	Ø	Ø
hCoV-19/env/Netherlands/Utrecht-93820-N/2020	G	A	T	G	Ø	G	G	T	Ø	Ø	Ø	Ø
hCoV-19/env/Netherlands/Utrecht-95550-N/2020	Ø	Ø	Ø	Ø	Ø	Ø	Ø	Ø	Ø	Ø	Ø	Ø
hCoV-19/env/Netherlands/Utrecht-94330-N/2020	G	A	T	G	C	G	G	Ø	Ø	G	G	C
hCoV-19/env/Netherlands/Utrecht-94331-N/2020	G	A	T	G	Ø	G	G	T	Ø	Ø	Ø	Ø
hCoV-19/env/Netherlands/Utrecht-92504-N/2020	G	A	T	G	Ø	G	G	T	T	G	G	C
hCoV-19/env/Netherlands/Utrecht-94602-N/2020	G	G	T	G	Ø	Ø	G	T	Ø	G	G	C
hCoV-19/env/Netherlands/Wervershoof-93950-N/2020	Ø	A	T	G	C	G	G	T	Ø	G	G	C
hCoV-19/env/Netherlands/Wervershoof-92723-I/2020	Ø	Ø	Ø	Ø	Ø	G	A	Ø	Ø	G	G	C
hCoV-19/env/Netherlands/Wervershoof-92723-N/2020	G	A	T	G	C	G	A	T	T	G	G	C

Isolate	24419	24521	24862	25273	25459	25489	25575	25936	26133	26144	26444	26642
Wuhan-Hu-1 (MN908947)	A	G	A	G	G	G	A	C	C	G	C	C
hCoV-19/env/Netherlands/Amersfoort-92848-I/2020	Ø	Ø	Ø	Ø	Ø	Ø	Ø	C	C	G	Ø	Ø
hCoV-19/env/Netherlands/Amersfoort-92848-N/2020	A	G	Ø	G	G	G	A	C	C	G	C	C
hCoV-19/env/Netherlands/Amersfoort-93818-N/2020	A	G	Ø	G	G	G	Ø	Ø	Ø	Ø	Ø	Ø
hCoV-19/env/Netherlands/Amersfoort-92503-N/2020	A	G	Ø	T	G	G	C	C	C	G	C	C
hCoV-19/env/Netherlands/AmsterdamWest-92852-I/2020	Ø	Ø	Ø	G	G	G	Ø	C	C	G	C	C
hCoV-19/env/Netherlands/AmsterdamWest-92852-N1/2020	A	G	A	G	G	G	A	C	C	G	C	C
hCoV-19/env/Netherlands/AmsterdamWest-92852-N2/2020	A	G	A	G	G	G	A	C	C	G	C	C
hCoV-19/env/Netherlands/AmsterdamWest-94978-N/2020	Ø	Ø	Ø	G	G	G	Ø	C	C	G	Ø	Ø
hCoV-19/env/Netherlands/AmsterdamWest-93822-N/2020	A	G	A	G	G	G	A	C	C	G	C	T
hCoV-19/env/Netherlands/AmsterdamWest-95552-N/2020	Ø	Ø	Ø	Ø	Ø	Ø	Ø	Ø	Ø	Ø	Ø	Ø
hCoV-19/env/Netherlands/AmsterdamWest-95794-N/2020	Ø	Ø	Ø	Ø	Ø	Ø	Ø	Ø	Ø	Ø	Ø	Ø
hCoV-19/env/Netherlands/AmsterdamWest-94334-N/2020	A	G	A	G	G	G	Ø	C	C	T	C	C

Isolate	24419	24521	24862	25273	25459	25489	25575	25936	26133	26144	26444	26642
hCoV-19/env/Netherlands/AmsterdamWest-92508-N/2020	A	G	A	G	G	G	A	C	C	G	C	C
hCoV-19/env/Netherlands/AmsterdamWest-94604-N/2020	Ø	Ø	Ø	Ø	Ø	Ø	Ø	Ø	Ø	Ø	Ø	Ø
hCoV-19/env/Netherlands/Apeldoorn-92502-I/2020	Ø	Ø	Ø	Ø	Ø	Ø	Ø	C	C	G	C	C
hCoV-19/env/Netherlands/Apeldoorn-92502-N/2020	A	G	Ø	G	G	G	Ø	C	C	G	C	C
hCoV-19/env/Belgium/Properinge-92949-I/2020	A	G	A	Ø	Ø	Ø	Ø	C	C	G	C	C
hCoV-19/env/Belgium/Properinge-92949-N/2020	A	G	Ø	G	G	G	A	C	C	G	C	Ø
hCoV-19/env/Belgium/Aartselaar-93030-I/2020	A	G	A	Ø	Ø	Ø	Ø	C	C	G	Ø	Ø
hCoV-19/env/Belgium/Aartselaar-93030-N/2020	A	G	A	G	G	G	Ø	C	C	G	C	C
hCoV-19/env/Belgium/Gent-93032-I/2020	Ø	Ø	A	Ø	Ø	Ø	Ø	C	C	G	Ø	Ø
hCoV-19/env/Belgium/Gent-93032-N/2020	A	G	A	Ø	Ø	Ø	Ø	C	C	G	C	C
hCoV-19/env/Belgium/Leuven-93034-I/2020	Ø	Ø	Ø	Ø	Ø	Ø	Ø	Ø	Ø	Ø	Ø	Ø
hCoV-19/env/Belgium/Leuven-93034-N/2020	A	G	Ø	Ø	Ø	Ø	Ø	Ø	Ø	Ø	C	Ø
hCoV-19/env/Belgium/Tienen-93036-I/2020	A	Ø	A	Ø	Ø	Ø	Ø	C	C	G	Ø	Ø
hCoV-19/env/Belgium/Tienen-93036-N/2020	A	G	A	G	G	G	A	C	C	G	C	C
hCoV-19/env/Belgium/Langemark-92943-N/2020	A	G	A	G	G	G	Ø	Ø	Ø	Ø	C	C
hCoV-19/env/Belgium/Lo-Reninge-92947-N/2020	Ø	Ø	A	G	G	G	A	C	C	T	C	C
hCoV-19/env/Netherlands/Beverwijk-92721-I/2020	G	G	A	Ø	Ø	Ø	Ø	C	C	G	Ø	C
hCoV-19/env/Netherlands/Beverwijk-92721-N/2020	A	G	A	G	G	G	A	C	C	G	C	C
hCoV-19/env/Netherlands/Delft-92965-I/2020	A	G	A	Ø	Ø	Ø	Ø	C	C	G	Ø	Ø
hCoV-19/env/Netherlands/Delft-92965-N/2020	A	G	A	Ø	T	G	A	C	C	G	C	C
hCoV-19/env/Netherlands/Delft-97044-N/2020	Ø	Ø	Ø	Ø	Ø	Ø	Ø	Ø	Ø	Ø	Ø	Ø
hCoV-19/env/Netherlands/Delft-94982-N/2020	Ø	Ø	Ø	Ø	Ø	Ø	Ø	C	C	G	Ø	Ø
hCoV-19/env/Netherlands/Delft-93825-I/2020	A	Ø	A	Ø	Ø	Ø	Ø	C	C	G	C	C
hCoV-19/env/Netherlands/Delft-93825-N/2020	Ø	Ø	Ø	G	G	G	Ø	C	C	G	C	C
hCoV-19/env/Netherlands/Delft-94337-N/2020	Ø	Ø	Ø	G	G	G	Ø	C	C	G	Ø	Ø
hCoV-19/env/Netherlands/Delft-94607-N/2020	Ø	Ø	Ø	Ø	Ø	Ø	Ø	Ø	Ø	Ø	Ø	Ø
hCoV-19/env/Netherlands/Franeker-92719-I/2020	A	G	A	G	G	G	A	C	C	G	C	C
hCoV-19/env/Netherlands/Franeker-92719-N1/2020	A	G	A	G	G	G	A	C	C	G	C	C
hCoV-19/env/Netherlands/Franeker-92719-N2/2020	A	G	Ø	G	G	G	Ø	C	C	G	C	C
hCoV-19/env/Netherlands/HeeswijkDinther-93948-N/2020	Ø	Ø	Ø	Ø	Ø	Ø	Ø	Ø	Ø	Ø	Ø	Ø
hCoV-19/env/Netherlands/HeeswijkDinther-92499-N/2020	A	G	G	G	G	G	A	C	C	G	C	Ø
hCoV-19/env/Netherlands/Katwoude-92722-I/2020	A	Ø	A	Ø	Ø	Ø	Ø	C	C	G	C	C
hCoV-19/env/Netherlands/Katwoude-92722-N/2020	A	T	Ø	G	G	G	A	C	C	G	C	C
hCoV-19/env/Netherlands/Schiphol-92851-I/2020	A	G	A	G	G	T	Ø	C	C	G	Ø	Ø
hCoV-19/env/Netherlands/Schiphol-92851-N/2020	A	G	A	G	G	T	Ø	C	C	G	C	C
hCoV-19/env/Netherlands/Schiphol-96927-I/2020	Ø	Ø	A	Ø	Ø	Ø	Ø	C	C	G	Ø	Ø
hCoV-19/env/Netherlands/Schiphol-96927-N/2020	Ø	Ø	Ø	G	G	G	Ø	T	C	G	C	Ø
hCoV-19/env/Netherlands/Schiphol-93823-I/2020	A	Ø	A	G	G	G	Ø	C	C	G	Ø	Ø
hCoV-19/env/Netherlands/Schiphol-93823-N/2020	Ø	Ø	A	Ø	G	G	A	C	T	G	C	C
hCoV-19/env/Netherlands/Schiphol-94335-I/2020	Ø	Ø	A	G	G	G	Ø	C	C	G	Ø	Ø
hCoV-19/env/Netherlands/Schiphol-94335-N/2020	Ø	Ø	A	Ø	Ø	Ø	Ø	Ø	Ø	Ø	Ø	Ø
hCoV-19/env/Netherlands/Schiphol-92506-I/2020	A	Ø	A	G	Ø	Ø	Ø	C	C	G	Ø	Ø
hCoV-19/env/Netherlands/Schiphol-92506-N/2020	A	G	Ø	Ø	G	G	A	C	C	G	C	C
hCoV-19/env/Netherlands/Schiphol-94605-I/2020	A	G	Ø	Ø	Ø	Ø	Ø	C	C	G	Ø	Ø
hCoV-19/env/Netherlands/Schiphol-94605-N/2020	Ø	Ø	Ø	Ø	Ø	Ø	Ø	Ø	Ø	Ø	Ø	Ø
hCoV-19/env/Netherlands/Tilburg-92853-I/2020	A	Ø	A	G	G	G	A	C	C	G	Ø	Ø
hCoV-19/env/Netherlands/Tilburg-92853-N/2020	A	G	A	G	G	G	A	C	C	G	T	C
hCoV-19/env/Netherlands/Tilburg-93828-N/2020	Ø	Ø	Ø	G	G	G	Ø	Ø	Ø	Ø	Ø	Ø
hCoV-19/env/Netherlands/Tilburg-94339-I/2020	A	G	A	G	G	G	A	C	C	G	C	C

Isolate	24419	24521	24862	25273	25459	25489	25575	25936	26133	26144	26444	26642
hCoV-19/env/Netherlands/Tilburg-94339-N/2020	Ø	Ø	Ø	Ø	Ø	Ø	Ø	Ø	Ø	Ø	Ø	Ø
hCoV-19/env/Netherlands/Tilburg-92509-I/2020	A	G	A	Ø	Ø	Ø	Ø	C	C	G	Ø	C
hCoV-19/env/Netherlands/Tilburg-92509-N/2020	Ø	Ø	Ø	G	G	G	A	C	C	G	C	C
hCoV-19/env/Netherlands/UtrechtOvervecht-92505-N/2020	A	G	A	G	G	G	A	C	C	T	C	C
hCoV-19/env/Netherlands/Utrecht-92849-I/2020	A	Ø	A	Ø	Ø	Ø	Ø	C	C	G	Ø	Ø
hCoV-19/env/Netherlands/Utrecht-92849-N/2020	A	G	Ø	G	G	G	Ø	C	C	G	C	C
hCoV-19/env/Netherlands/Utrecht-94976-N/2020	Ø	Ø	Ø	Ø	Ø	Ø	Ø	C	C	G	Ø	Ø
hCoV-19/env/Netherlands/Utrecht-93820-N/2020	Ø	Ø	Ø	Ø	Ø	Ø	Ø	Ø	Ø	Ø	C	C
hCoV-19/env/Netherlands/Utrecht-95550-N/2020	Ø	Ø	Ø	Ø	Ø	Ø	Ø	C	C	G	Ø	Ø
hCoV-19/env/Netherlands/Utrecht-94330-N/2020	A	Ø	Ø	Ø	Ø	Ø	Ø	Ø	Ø	Ø	Ø	Ø
hCoV-19/env/Netherlands/Utrecht-94331-N/2020	A	G	A	Ø	Ø	Ø	Ø	Ø	Ø	Ø	Ø	Ø
hCoV-19/env/Netherlands/Utrecht-92504-N/2020	A	G	A	G	G	G	A	C	C	G	C	C
hCoV-19/env/Netherlands/Utrecht-94602-N/2020	A	G	Ø	Ø	Ø	Ø	Ø	C	C	G	C	C
hCoV-19/env/Netherlands/Wervershoof-93950-N/2020	A	G	A	Ø	Ø	Ø	Ø	Ø	Ø	Ø	C	C
hCoV-19/env/Netherlands/Wervershoof-92723-I/2020	A	G	Ø	G	G	G	Ø	C	C	G	Ø	Ø
hCoV-19/env/Netherlands/Wervershoof-92723-N/2020	A	G	A	G	G	G	A	C	C	G	C	C

Isolate	26735	26803	27046	27128	27296	27478	28085	28110	28115	28144	28736	28851
Wuhan-Hu-1 (MN908947)	C	G	C	C	T	G	G	T	C	T	G	G
hCoV-19/env/Netherlands/Amersfoort-92848-I/2020	Ø	Ø	C	C	T	G	G	T	C	C	G	G
hCoV-19/env/Netherlands/Amersfoort-92848-N/2020	C	G	C	C	T	G	G	T	C	T	G	G
hCoV-19/env/Netherlands/Amersfoort-93818-N/2020	Ø	Ø	C	C	T	G	G	T	C	T	Ø	Ø
hCoV-19/env/Netherlands/Amersfoort-92503-N/2020	C	G	C	T	T	G	G	T	C	C	G	G
hCoV-19/env/Netherlands/AmsterdamWest-92852-I/2020	Ø	Ø	C	C	T	G	G	T	C	T	G	G
hCoV-19/env/Netherlands/AmsterdamWest-92852-N1/2020	C	G	C	C	T	G	G	T	C	T	G	G
hCoV-19/env/Netherlands/AmsterdamWest-92852-N2/2020	C	G	C	C	T	G	G	T	C	T	G	G
hCoV-19/env/Netherlands/AmsterdamWest-94978-N/2020	Ø	Ø	Ø	Ø	Ø	Ø	G	T	C	Ø	G	Ø
hCoV-19/env/Netherlands/AmsterdamWest-93822-N/2020	C	G	C	C	T	G	G	T	C	T	G	G
hCoV-19/env/Netherlands/AmsterdamWest-95552-N/2020	Ø	Ø	Ø	Ø	Ø	Ø	Ø	Ø	Ø	Ø	Ø	Ø
hCoV-19/env/Netherlands/AmsterdamWest-95794-N/2020	Ø	Ø	Ø	Ø	Ø	Ø	Ø	Ø	Ø	Ø	Ø	Ø
hCoV-19/env/Netherlands/AmsterdamWest-94334-N/2020	C	G	C	C	T	G	G	T	C	T	G	T
hCoV-19/env/Netherlands/AmsterdamWest-92508-N/2020	C	G	C	C	T	G	G	T	C	T	G	G
hCoV-19/env/Netherlands/AmsterdamWest-94604-N/2020	Ø	Ø	Ø	Ø	Ø	Ø	Ø	Ø	Ø	Ø	Ø	Ø
hCoV-19/env/Netherlands/Apeldoorn-92502-I/2020	C	G	Ø	Ø	Ø	Ø	Ø	Ø	Ø	Ø	Ø	Ø
hCoV-19/env/Netherlands/Apeldoorn-92502-N/2020	C	G	C	C	T	G	G	T	C	T	G	G
hCoV-19/env/Belgium/Properinge-92949-I/2020	C	G	C	C	T	G	G	T	C	T	G	G
hCoV-19/env/Belgium/Properinge-92949-N/2020	C	G	C	C	Ø	G	Ø	Ø	Ø	Ø	G	G
hCoV-19/env/Belgium/Aartselaar-93030-I/2020	Ø	Ø	C	C	T	G	G	T	C	T	G	G
hCoV-19/env/Belgium/Aartselaar-93030-N/2020	C	G	C	C	T	G	G	T	C	T	G	G
hCoV-19/env/Belgium/Gent-93032-I/2020	Ø	Ø	C	C	T	G	G	T	C	T	G	G
hCoV-19/env/Belgium/Gent-93032-N/2020	C	G	C	C	T	G	G	T	C	T	G	G
hCoV-19/env/Belgium/Leuven-93034-I/2020	Ø	Ø	C	C	T	G	G	C	C	T	A	G
hCoV-19/env/Belgium/Leuven-93034-N/2020	C	G	C	C	T	G	G	T	C	T	G	G
hCoV-19/env/Belgium/Tienen-93036-I/2020	Ø	Ø	C	C	T	G	Ø	Ø	Ø	T	G	Ø
hCoV-19/env/Belgium/Tienen-93036-N/2020	C	G	C	C	T	G	G	T	C	T	G	G
hCoV-19/env/Belgium/Langemark-92943-N/2020	C	G	C	C	A	G	G	T	C	T	G	G
hCoV-19/env/Belgium/Lo-Reninge-92947-N/2020	C	G	T	C	T	G	G	T	C	T	G	G

Isolate	26735	26803	27046	27128	27296	27478	28085	28110	28115	28144	28736	28851
hCoV-19/env/Netherlands/Beverwijk-92721-I/2020	Ø	Ø	C	C	T	G	G	T	C	T	G	G
hCoV-19/env/Netherlands/Beverwijk-92721-N/2020	C	G	C	C	T	G	G	T	C	T	G	G
hCoV-19/env/Netherlands/Delft-92965-I/2020	Ø	Ø	C	C	T	G	G	T	T	T	G	G
hCoV-19/env/Netherlands/Delft-92965-N/2020	C	G	C	C	T	G	G	T	C	T	G	G
hCoV-19/env/Netherlands/Delft-97044-N/2020	Ø	Ø	C	C	T	G	Ø	Ø	Ø	Ø	Ø	Ø
hCoV-19/env/Netherlands/Delft-94982-N/2020	Ø	Ø	Ø	Ø	Ø	Ø	Ø	Ø	Ø	Ø	Ø	Ø
hCoV-19/env/Netherlands/Delft-93825-I/2020	C	G	C	C	T	G	G	T	C	T	G	G
hCoV-19/env/Netherlands/Delft-93825-N/2020	C	G	C	C	T	G	G	T	C	T	Ø	Ø
hCoV-19/env/Netherlands/Delft-94337-N/2020	Ø	Ø	C	C	T	G	G	T	C	T	Ø	Ø
hCoV-19/env/Netherlands/Delft-94607-N/2020	Ø	Ø	Ø	Ø	Ø	Ø	Ø	Ø	Ø	Ø	Ø	Ø
hCoV-19/env/Netherlands/Franeker-92719-I/2020	C	Ø	C	C	T	G	G	T	C	T	G	G
hCoV-19/env/Netherlands/Franeker-92719-N1/2020	C	G	C	C	T	G	G	T	C	T	G	G
hCoV-19/env/Netherlands/Franeker-92719-N2/2020	C	G	C	C	T	G	G	T	C	T	Ø	Ø
hCoV-19/env/Netherlands/HeeswijkDinther-93948-N/2020	Ø	Ø	C	C	T	G	Ø	Ø	Ø	Ø	G	Ø
hCoV-19/env/Netherlands/HeeswijkDinther-92499-N/2020	C	G	C	C	T	G	G	T	C	T	G	G
hCoV-19/env/Netherlands/Katwoude-92722-I/2020	C	G	C	C	T	G	G	T	C	T	G	G
hCoV-19/env/Netherlands/Katwoude-92722-N/2020	C	G	C	C	T	G	G	T	C	T	G	G
hCoV-19/env/Netherlands/Schiphol-92851-I/2020	Ø	Ø	C	C	T	G	Ø	Ø	Ø	T	G	G
hCoV-19/env/Netherlands/Schiphol-92851-N/2020	C	T	C	C	Ø	G	G	T	C	T	G	G
hCoV-19/env/Netherlands/Schiphol-96927-I/2020	C	G	C	C	T	T	Ø	Ø	Ø	T	G	G
hCoV-19/env/Netherlands/Schiphol-96927-N/2020	Ø	Ø	Ø	Ø	Ø	Ø	G	T	C	T	G	Ø
hCoV-19/env/Netherlands/Schiphol-93823-I/2020	Ø	Ø	Ø	C	T	Ø	Ø	Ø	Ø	T	G	G
hCoV-19/env/Netherlands/Schiphol-93823-N/2020	C	G	C	C	T	G	G	T	C	T	G	G
hCoV-19/env/Netherlands/Schiphol-94335-I/2020	Ø	Ø	Ø	C	T	Ø	Ø	Ø	Ø	T	G	Ø
hCoV-19/env/Netherlands/Schiphol-94335-N/2020	Ø	Ø	C	C	T	G	G	T	C	T	G	G
hCoV-19/env/Netherlands/Schiphol-92506-I/2020	Ø	G	C	C	T	G	G	T	C	T	G	G
hCoV-19/env/Netherlands/Schiphol-92506-N/2020	C	G	C	C	T	G	G	T	C	T	G	G
hCoV-19/env/Netherlands/Schiphol-94605-I/2020	Ø	Ø	Ø	Ø	Ø	Ø	G	T	C	T	Ø	Ø
hCoV-19/env/Netherlands/Schiphol-94605-N/2020	Ø	Ø	Ø	Ø	Ø	Ø	Ø	Ø	Ø	Ø	Ø	Ø
hCoV-19/env/Netherlands/Tilburg-92853-I/2020	C	Ø	C	C	T	G	G	T	C	T	G	G
hCoV-19/env/Netherlands/Tilburg-92853-N/2020	C	G	C	C	T	G	G	T	C	T	G	G
hCoV-19/env/Netherlands/Tilburg-93828-N/2020	Ø	Ø	C	C	T	G	G	T	C	T	G	G
hCoV-19/env/Netherlands/Tilburg-94339-I/2020	C	G	C	C	T	G	G	T	C	T	G	G
hCoV-19/env/Netherlands/Tilburg-94339-N/2020	Ø	Ø	Ø	Ø	Ø	Ø	Ø	Ø	Ø	Ø	Ø	Ø
hCoV-19/env/Netherlands/Tilburg-92509-I/2020	C	Ø	Ø	C	T	Ø	Ø	Ø	Ø	T	G	Ø
hCoV-19/env/Netherlands/Tilburg-92509-N/2020	C	G	C	C	T	G	G	T	C	T	G	G
hCoV-19/env/Netherlands/UtrechtOvervecht-92505-N/2020	C	G	C	C	T	G	G	T	C	T	G	G
hCoV-19/env/Netherlands/Utrecht-92849-I/2020	Ø	Ø	C	C	T	G	G	T	C	T	G	G
hCoV-19/env/Netherlands/Utrecht-92849-N/2020	C	G	C	C	T	G	G	T	C	T	G	G
hCoV-19/env/Netherlands/Utrecht-94976-N/2020	Ø	Ø	Ø	Ø	Ø	Ø	Ø	Ø	Ø	Ø	Ø	Ø
hCoV-19/env/Netherlands/Utrecht-93820-N/2020	C	G	C	C	T	G	T	T	C	T	G	G
hCoV-19/env/Netherlands/Utrecht-95550-N/2020	Ø	Ø	Ø	Ø	Ø	Ø	Ø	Ø	Ø	Ø	Ø	Ø
hCoV-19/env/Netherlands/Utrecht-94330-N/2020	Ø	Ø	C	C	T	G	G	T	C	T	Ø	Ø
hCoV-19/env/Netherlands/Utrecht-94331-N/2020	Ø	Ø	Ø	Ø	Ø	Ø	Ø	Ø	Ø	Ø	Ø	Ø
hCoV-19/env/Netherlands/Utrecht-92504-N/2020	C	G	C	C	T	G	G	T	C	T	G	G
hCoV-19/env/Netherlands/Utrecht-94602-N/2020	C	G	C	C	T	G	G	T	C	T	G	G
hCoV-19/env/Netherlands/Wervershoof-93950-N/2020	C	G	C	C	T	G	G	T	C	T	Ø	Ø
hCoV-19/env/Netherlands/Wervershoof-92723-I/2020	Ø	Ø	Ø	Ø	Ø	Ø	Ø	Ø	Ø	Ø	G	G
hCoV-19/env/Netherlands/Wervershoof-92723-N/2020	T	G	C	C	T	G	G	T	C	T	G	G

Isolate	28881	29058	29128	29253	29308	29366	29422	29520
Wuhan-Hu-1 (MN908947)	GGG	G	T	C	T	C	G	C
hCoV-19/env/Netherlands/Amersfoort-92848-I/2020	Ø	Ø	Ø	Ø	Ø	Ø	Ø	Ø
hCoV-19/env/Netherlands/Amersfoort-92848-N/2020	GGG	G	T	C	T	C	G	C
hCoV-19/env/Netherlands/Amersfoort-93818-N/2020	Ø	Ø	T	C	T	C	G	C
hCoV-19/env/Netherlands/Amersfoort-92503-N/2020	AAC	G	T	Ø	Ø	Ø	Ø	Ø
hCoV-19/env/Netherlands/AmsterdamWest-92852-I/2020	GGG	G	T	Ø	T	C	G	Ø
hCoV-19/env/Netherlands/AmsterdamWest-92852-N1/2020	GGG	G	T	C	T	C	G	C
hCoV-19/env/Netherlands/AmsterdamWest-92852-N2/2020	GGG	G	T	C	T	C	G	C
hCoV-19/env/Netherlands/AmsterdamWest-94978-N/2020	Ø	Ø	T	C	T	C	G	C
hCoV-19/env/Netherlands/AmsterdamWest-93822-N/2020	GGG	G	T	C	T	C	G	C
hCoV-19/env/Netherlands/AmsterdamWest-95552-N/2020	Ø	Ø	Ø	Ø	Ø	Ø	Ø	Ø
hCoV-19/env/Netherlands/AmsterdamWest-95794-N/2020	Ø	Ø	Ø	Ø	Ø	Ø	Ø	Ø
hCoV-19/env/Netherlands/AmsterdamWest-94334-N/2020	AAC	G	T	Ø	Ø	Ø	Ø	Ø
hCoV-19/env/Netherlands/AmsterdamWest-92508-N/2020	GGG	G	T	C	T	C	G	C
hCoV-19/env/Netherlands/AmsterdamWest-94604-N/2020	Ø	Ø	T	C	T	C	G	C
hCoV-19/env/Netherlands/Apeldoorn-92502-I/2020	Ø	Ø	Ø	Ø	Ø	Ø	Ø	Ø
hCoV-19/env/Netherlands/Apeldoorn-92502-N/2020	GGG	G	T	Ø	Ø	Ø	Ø	Ø
hCoV-19/env/Belgium/Properinge-92949-I/2020	Ø	G	T	C	T	C	G	Ø
hCoV-19/env/Belgium/Properinge-92949-N/2020	GGG	G	T	Ø	Ø	Ø	Ø	Ø
hCoV-19/env/Belgium/Aartselaar-93030-I/2020	AAC	G	T	C	T	C	G	C
hCoV-19/env/Belgium/Aartselaar-93030-N/2020	GGG	G	T	C	T	C	G	C
hCoV-19/env/Belgium/Gent-93032-I/2020	AAC	G	T	C	T	C	G	C
hCoV-19/env/Belgium/Gent-93032-N/2020	GGG	G	T	C	T	C	G	C
hCoV-19/env/Belgium/Leuven-93034-I/2020	GGG	G	T	Ø	T	Ø	G	Ø
hCoV-19/env/Belgium/Leuven-93034-N/2020	AAC	G	T	C	T	C	G	C
hCoV-19/env/Belgium/Tienen-93036-I/2020	Ø	Ø	T	C	T	C	G	Ø
hCoV-19/env/Belgium/Tienen-93036-N/2020	AAC	G	T	C	T	C	G	C
hCoV-19/env/Belgium/Langemark-92943-N/2020	AAC	G	T	C	T	C	G	C
hCoV-19/env/Belgium/Lo-Reninge-92947-N/2020	AAC	G	T	C	T	T	G	C
hCoV-19/env/Netherlands/Beverwijk-92721-I/2020	GGG	G	T	Ø	Ø	Ø	Ø	Ø
hCoV-19/env/Netherlands/Beverwijk-92721-N/2020	GGG	G	T	C	T	C	G	C
hCoV-19/env/Netherlands/Delft-92965-I/2020	AAC	G	T	C	T	C	G	C
hCoV-19/env/Netherlands/Delft-92965-N/2020	AAC	G	T	Ø	Ø	Ø	Ø	Ø
hCoV-19/env/Netherlands/Delft-97044-N/2020	Ø	Ø	Ø	Ø	Ø	Ø	Ø	Ø
hCoV-19/env/Netherlands/Delft-94982-N/2020	Ø	Ø	Ø	Ø	Ø	Ø	Ø	Ø
hCoV-19/env/Netherlands/Delft-93825-I/2020	GGG	G	T	C	T	C	G	Ø
hCoV-19/env/Netherlands/Delft-93825-N/2020	Ø	Ø	Ø	Ø	Ø	Ø	Ø	Ø
hCoV-19/env/Netherlands/Delft-94337-N/2020	Ø	Ø	T	C	T	C	T	C
hCoV-19/env/Netherlands/Delft-94607-N/2020	Ø	Ø	T	C	T	C	G	C
hCoV-19/env/Netherlands/Franeker-92719-I/2020	AAC	G	T	C	T	C	G	C
hCoV-19/env/Netherlands/Franeker-92719-N1/2020	AAC	G	T	C	T	C	G	C
hCoV-19/env/Netherlands/Franeker-92719-N2/2020	Ø	Ø	T	C	T	C	G	C
hCoV-19/env/Netherlands/HeeswijkDinther-93948-N/2020	Ø	Ø	T	C	T	C	G	C
hCoV-19/env/Netherlands/HeeswijkDinther-92499-N/2020	GGG	G	T	C	T	C	G	C
hCoV-19/env/Netherlands/Katwoude-92722-I/2020	Ø	G	T	Ø	Ø	Ø	Ø	Ø
hCoV-19/env/Netherlands/Katwoude-92722-N/2020	AAC	G	T	C	T	C	G	C

Isolate	28881	29058	29128	29253	29308	29366	29422	29520
hCoV-19/env/Netherlands/Schiphol-92851-I/2020	AAC	G	T	C	T	C	G	Ø
hCoV-19/env/Netherlands/Schiphol-92851-N/2020	GGG	G	T	C	T	C	G	C
hCoV-19/env/Netherlands/Schiphol-96927-I/2020	Ø	G	T	G	C	C	G	C
hCoV-19/env/Netherlands/Schiphol-96927-N/2020	Ø	Ø	T	C	T	C	G	C
hCoV-19/env/Netherlands/Schiphol-93823-I/2020	Ø	G	T	C	T	C	G	Ø
hCoV-19/env/Netherlands/Schiphol-93823-N/2020	AAC	G	T	Ø	Ø	Ø	Ø	Ø
hCoV-19/env/Netherlands/Schiphol-94335-I/2020	Ø	Ø	Ø	C	T	C	G	C
hCoV-19/env/Netherlands/Schiphol-94335-N/2020	AAC	G	T	C	T	C	G	C
hCoV-19/env/Netherlands/Schiphol-92506-I/2020	Ø	Ø	T	C	T	C	G	C
hCoV-19/env/Netherlands/Schiphol-92506-N/2020	GGG	G	T	C	T	C	G	C
hCoV-19/env/Netherlands/Schiphol-94605-I/2020	Ø	Ø	Ø	C	T	Ø	G	Ø
hCoV-19/env/Netherlands/Schiphol-94605-N/2020	Ø	Ø	T	C	T	C	G	C
hCoV-19/env/Netherlands/Tilburg-92853-I/2020	Ø	G	T	C	T	C	G	C
hCoV-19/env/Netherlands/Tilburg-92853-N/2020	GGG	G	T	C	T	C	G	T
hCoV-19/env/Netherlands/Tilburg-93828-N/2020	AAC	G	T	C	T	C	G	C
hCoV-19/env/Netherlands/Tilburg-94339-I/2020	AAC	G	T	C	T	C	G	C
hCoV-19/env/Netherlands/Tilburg-94339-N/2020	Ø	Ø	Ø	Ø	Ø	Ø	Ø	Ø
hCoV-19/env/Netherlands/Tilburg-92509-I/2020	Ø	Ø	T	C	T	C	G	C
hCoV-19/env/Netherlands/Tilburg-92509-N/2020	GGG	G	T	C	T	C	G	C
hCoV-19/env/Netherlands/UtrechtOvervecht-92505-N/2020	GGG	G	T	C	T	C	G	C
hCoV-19/env/Netherlands/Utrecht-92849-I/2020	GGG	G	T	C	T	C	G	C
hCoV-19/env/Netherlands/Utrecht-92849-N/2020	Ø	Ø	T	C	T	C	G	C
hCoV-19/env/Netherlands/Utrecht-94976-N/2020	Ø	Ø	Ø	Ø	Ø	Ø	Ø	Ø
hCoV-19/env/Netherlands/Utrecht-93820-N/2020	GGG	G	C	C	T	C	G	C
hCoV-19/env/Netherlands/Utrecht-95550-N/2020	Ø	Ø	Ø	Ø	Ø	Ø	Ø	Ø
hCoV-19/env/Netherlands/Utrecht-94330-N/2020	Ø	Ø	T	C	T	C	G	C
hCoV-19/env/Netherlands/Utrecht-94331-N/2020	Ø	Ø	T	C	T	C	G	C
hCoV-19/env/Netherlands/Utrecht-92504-N/2020	GGG	G	T	C	T	C	G	C
hCoV-19/env/Netherlands/Utrecht-94602-N/2020	GGG	A	T	Ø	Ø	Ø	Ø	Ø
hCoV-19/env/Netherlands/Wervershoof-93950-N/2020	Ø	Ø	Ø	Ø	Ø	Ø	Ø	Ø
hCoV-19/env/Netherlands/Wervershoof-92723-I/2020	Ø	G	T	C	T	C	G	C
hCoV-19/env/Netherlands/Wervershoof-92723-N/2020	GGG	G	T	C	T	C	G	C

*Ø did not meet the parameters set for generating a consensus; these positions are highlighted in grey. Mutations compared with the Wuhan-Hu-1 reference sequence are highlighted in yellow. SNP, single-nucleotide polymorphism.

Appendix Table 2. Summary of the mutations detected in SARS-CoV-2 genome consensus sequences from wastewater samples determined by both Nanopore and Illumina sequencing, the Netherlands and Belgium*

Position	Ref	Alt	Position coverage	Feature	Effect	AA Ref‡	AA Alt§	NL frequency, %¶	BE frequency, %¶	Global frequency, %¶
104	T	C	Illumina	3'-UTR	Unknown	-	-	0.000	0.000	0.000
187	A	G	High	3'-UTR	Unknown	-	-	1.360	0.450	0.750
241	C	T	High	3'-UTR	Unknown	-	-	80.181	87.950	74.273
331	G	T	High	ORF1ab	NS	Q	T	0.000	0.000	0.000
430	A	C	High	ORF1ab	NS	E	D	0.000	0.000	0.000
448	G	T	Illumina	ORF1ab	NS	L	F	0.000	0.000	0.002
493	T	C	High	ORF1ab	Syn	A	A	0.000	0.000	0.002
514	T	C	High	ORF1ab	Syn	H	H	11.593	1.689	0.572
515	G	T	Illumina	ORF1ab	NS	V	F	0.000	0.000	0.000
702	G	T	Illumina	ORF1ab	NS	G	V	0.000	0.000	0.000
1059	C	T	High	ORF1ab	NS	T	I	9.585	10.698	17.338
1191	C	T	High	ORF1ab	NS	P	L	0.000	0.000	0.073
1338	G	T	Illumina	ORF1ab	NS	G	V	0.000	0.000	0.000
1594	C	T	Illumina	ORF1ab	Syn	S	S	15.220	0.338	0.510
1605	ATG	---	High	ORF1ab	Del	D	-	12.370	1.577	2.397
1704	T	A	High	ORF1ab	NS	F	Y	0.000	0.000	0.000
1802	A	G	High	ORF1ab	NS	K	E	0.000	0.000	0.002
1839	A	C	Illumina	ORF1ab	NS	E	A	0.000	0.000	0.000
1944	G	T	Low	ORF1ab	NS	R	L	0.000	0.000	0.004
1997	C	T	High	ORF1ab	Syn	L	L	1.166	0.000	0.051
2144	G	A	High	ORF1ab	NS	V	I	0.000	0.000	0.009
2527	G	T	Illumina	ORF1ab	NS	E	D	0.194	0.000	0.009
3025	G	T	Low	ORF1ab	NS	M	I	0.000	0.000	0.002
3037	C	T	High	ORF1ab	Syn	F	F	80.181	87.950	75.148
3194	G	T	High	ORF1ab	NS	E	Stop	0.000	0.000	0.000
3373	C	A	High	ORF1ab	NS	D	E	0.453	1.014	0.512
3521	G	T	High	ORF1ab	NS	E	Stop	0.000	0.000	0.000
3778	A	G	High	ORF1ab	NS	T	R	0.389	0.000	0.015
4391	C	T	High	ORF1ab	NS	R	Stop	0.000	0.000	0.000
4655	C	T	High	ORF1ab	NS	R	W	0.194	0.113	0.033
4720	G	T	High	ORF1ab	Syn	A	A	0.065	0.000	0.038
4870	A	G	High	ORF1ab	Syn	V	V	0.000	0.000	0.004
5230	G	T	High	ORF1ab	NS	K	N	0.000	0.000	0.074
5633	G	T	High	ORF1ab	NS	G	C	0.000	0.000	0.000
6075	T	C	High	ORF1ab	NS	F	S	0.000	0.000	0.000
6077	G	T	High	ORF1ab	NS	A	S	0.000	0.000	0.000
7063	C	T	High	ORF1ab	Syn	Y	Y	0.065	0.000	0.004
7577	G	T	High	ORF1ab	NS	A	S	0.000	0.000	0.000
8266	C	T	High	ORF1ab	Syn	G	G	0.000	0.000	0.011
8290	C	T	High	ORF1ab	Syn	L	L	0.194	0.000	0.056
8322	G	T	High	ORF1ab	NS	R	L	0.000	0.000	0.000
8946	A	T	Illumina	ORF1ab	NS	N	I	0.000	0.000	0.000
9063	G	A	High	ORF1ab	NS	C	Y	0.000	0.000	0.000
9269	G	T	High	ORF1ab	NS	T	C	0.000	0.000	0.000
9426	C	T	Low	ORF1ab	NS	A	V	0.000	0.000	0.000
9477	T	A	Low	ORF1ab	NS	F	Y	0.842	0.000	1.685
9733	C	T	High	ORF1ab	Syn	F	F	0.389	0.000	0.040

Position	Ref	Alt	Position coverage	Feature	Effect	AA Ref‡	AA Alt§	NL frequency, %¶	BE frequency, %¶	Global frequency, %¶
10097	G	A	High	ORF1ab	NS	G	S	1.036	3.491	3.951
10349	C	T	Low	ORF1ab	NS	P	S	0.000	0.000	0.000
10525	C	T	High	ORF1ab	Syn	V	V	0.000	0.000	0.096
10561	T	C	High	ORF1ab	Syn	T	T	1.813	0.000	0.076
10819	T	C	Illumina	ORF1ab	Syn	A	A	0.000	0.000	0.000
10929	C	T	High	ORF1ab	NS	T	I	0.000	0.000	0.000
10933	T	C	Illumina	ORF1ab	Syn	P	P	0.000	0.000	0.004
11083	G	T	High	ORF1ab	NS	L	F	5.635	7.320	10.996
11484	T	C	Illumina	ORF1ab	NS	V	A	0.000	0.000	0.000
11596	G	T	Illumina	ORF1ab	NS	Q	H	3.303	0.113	0.109
11627	T	C	High	ORF1ab	NS	Y	H	0.000	0.000	0.000
11648	G	T	High	ORF1ab	NS	G	C	0.000	0.000	0.000
11839	A	G	High	ORF1ab	Syn	V	V	0.000	2.477	0.107
12754	C	T	Low	ORF1ab	Syn	C	C	0.000	0.000	0.016
12970	C	T	High	ORF1ab	Syn	N	N	0.000	0.000	0.029
13210	T	A	High	ORF1ab	NS	N	K	0.000	0.000	0.000
13214	G	T	High	ORF1ab	NS	D	Y	0.000	0.000	0.000
13237	A	G	High	ORF1ab	Syn	A	A	0.000	0.000	0.002
13242	G	T	High	ORF1ab	NS	C	F	0.000	0.000	0.002
13374	G	T	High	ORF1ab	NS	C	F	0.000	0.000	0.000
13536	C	T	High	ORF1ab	Syn	Y	Y	1.036	3.378	1.369
13779	T	C	High	ORF1ab	Syn	H	H	0.000	0.000	0.002
13972	T	G	High	ORF1ab	NS	L	V	0.000	0.000	0.000
14408	C	T	High	ORF1ab	NS	P	L	80.181	88.063	75.123
14618	C	T	High	ORF1ab	NS	T	I	0.000	0.000	0.002
15324	C	T	High	ORF1ab	Syn	N	N	2.526	22.748	2.330
15495	C	A	High	ORF1ab	Syn	A	A	0.000	0.000	0.000
15939	T	C	Illumina	ORF1ab	Syn	D	D	0.000	0.000	0.007
16017	C	T	High	ORF1ab	Syn	F	F	0.583	0.000	0.067
16061	A	C	High	ORF1ab	NS	N	T	0.000	0.000	0.000
16468	C	T	High	ORF1ab	NS	P	S	0.000	0.000	0.040
16538	ATA	---	High	ORF1ab	Del	N	-	0.000	0.000	0.000
16690	G	T	Illumina	ORF1ab	NS	A	S	0.000	0.000	0.000
17129	A	G	Illumina	ORF1ab	NS	Y	C	0.000	0.000	0.000
17212	C	A	High	ORF1ab	NS	P	T	0.000	0.000	0.000
17410	C	T	High	ORF1ab	NS	R	C	5.959	1.464	0.294
17504	A	G	Illumina	ORF1ab	NS	N	S	0.000	0.000	0.000
17822	C	T	Low	ORF1ab	NS	P	L	0.000	0.000	0.011
17827	C	T	High	ORF1ab	NS	Q	Stop	0.000	0.000	0.000
18029	C	T	High	ORF1ab	NS	A	V	0.130	0.000	0.071
18484	C	T	Illumina	ORF1ab	NS	L	F	0.000	0.000	0.000
18838	G	T	High	ORF1ab	NS	A	S	0.000	0.000	0.007
18998	C	T	High	ORF1ab	NS	A	V	0.000	0.000	0.719
19072	G	T	High	ORF1ab	NS	D	Y	0.000	0.000	0.044
19086	G	T	High	ORF1ab	NS	K	N	0.000	0.113	0.042
19170	C	T	High	ORF1ab	Syn	F	F	1.101	0.000	0.764
19273	C	T	High	ORF1ab	NS	P	L	0.000	0.113	0.016
19480	G	A	High	ORF1ab	NS	G	S	0.000	0.000	0.002
19509	G	A	High	ORF1ab	Syn	E	E	1.101	0.000	0.479

Position	Ref	Alt	Position coverage	Feature	Effect	AA Ref‡	AA Alt§	NL frequency, %¶	BE frequency, %¶	Global frequency, %¶
19586	C	T	High	ORF1ab	NS	T	I	0.065	0.000	0.013
19839	T	C	High	ORF1ab	Syn	N	N	0.777	8.221	1.843
20087	G	T	High	ORF1ab	NS	G	V	0.000	0.000	0.000
20234	C	T	Illumina	ORF1ab	NS	P	L	0.000	0.000	0.025
20268	A	G	Illumina	ORF1ab	Syn	L	L	6.801	1.802	5.057
20900	G	T	Illumina	ORF1ab	NS	G	V	0.000	0.000	0.000
21137	A	G	High	ORF1ab	NS	K	R	0.000	0.225	0.236
21140	T	C	Illumina	ORF1ab	NS	L	P	0.000	0.000	0.000
21142	G	T	Illumina	ORF1ab	NS	A	S	0.000	0.000	0.002
21207	C	T	Low	ORF1ab	Syn	L	L	0.000	0.000	0.013
23009	G	T	High	S	NS	V	F	0.000	0.113	0.013
23403	A	G	High	S	NS	D	G	80.117	87.950	75.308
23622	T	C	High	S	NS	V	A	0.000	0.000	0.000
23877	T	G	Illumina	S	NS	V	G	0.000	0.000	0.000
24077	G	T	Illumina	S	NS	D	Y	0.518	0.000	0.330
24095	G	T	High	S	NS	A	S	0.000	0.113	0.064
24159	C	T	High	S	NS	C	I	0.000	0.000	0.000
24419	A	G	Illumina	S	NS	N	Y	0.000	0.000	0.000
24521	G	T	High	S	NS	V	F	0.000	0.000	0.002
24862	A	G	High	S	Syn	T	T	8.614	0.338	0.461
25273	G	T	High	S	NS	M	I	0.000	0.000	0.038
25459	G	T	High	ORF3a	NS	A	S	0.000	0.000	0.118
25489	G	T	High	ORF3a	NS	A	S	0.000	0.113	0.016
25575	A	C	Low	ORF3a	NS	K	N	0.194	0.000	0.153
25936	C	T	High	ORF3a	NS	H	Y	0.000	0.000	0.020
26133	C	T	High	ORF3a	Syn	H	H	0.000	0.000	0.009
26144	G	T	High	ORF3a	NS	G	V	4.469	5.293	7.339
26444	C	T	High	E	NS	S	F	0.000	0.000	0.000
26642	C	T	Low	M	Syn	A	A	0.065	0.000	0.007
26735	C	T	High	M	Syn	Y	Y	0.518	0.000	1.037
26803	G	T	High	M	NS	S	I	0.000	0.000	0.002
27046	C	T	High	M	NS	T	M	9.391	3.378	1.438
27128	C	T	High	M	Syn	G	G	0.000	0.000	0.094
27296	T	A	High	ORF6	NS	I	N	0.000	0.000	0.000
27478	G	T	Illumina	ORF7a	NS	V	L	0.065	0.225	0.022
28085	G	T	Low	ORF8	NS	E	D	0.000	0.000	0.005
28110	T	C	Illumina	ORF8	NS	Y	H	0.065	0.000	0.016
28115	C	T	Illumina	ORF8	Syn	I	I	0.000	0.000	0.005
28144	T	C	High	ORF8	NS	L	S	1.231	0.338	7.601
28736	G	A	Illumina	N	NS	A	T	0.000	0.000	0.000
28851	G	T	Low	N	NS	S	I	0.130	0.000	0.962
28881	GGG	AAC	High	N	NS	RG	KR	18.977	30.856	29.931
29058	G	A	Low	N	NS	R	H	0.000	0.000	0.002
29128	T	C	High	N	Syn	N	N	0.000	0.000	0.002
29253	C	G	Illumina	N	NS	S	W	0.000	0.000	0.000
29308	T	C	Illumina	N	Syn	N	N	0.000	0.000	0.002
29366	C	T	High	N	NS	P	S	0.000	0.000	0.020
29422	G	T	High	N	Syn	P	P	3.497	0.000	0.154
29520	C	T	High	N	NS	S	L	0.000	0.000	0.013

Position	Ref	Alt	Position coverage	Feature	Effect	AA Ref‡	AA Alt§	NL frequency, %¶	BE frequency, %¶	Global frequency, %¶

*Positions are given with respect to Wuhan-Hu-1 (MN908947). Del, deletion; NS, non-synonymous; SARS-CoV-2, severe acute respiratory syndrome coronavirus 2; SYN, synonymous mutation.
†Coverage is indicated as Illumina if the mutation was found only in Illumina sequencing (coverage of the position >5X, Phred score >30), High if the nanopore coverage was >30X, and Low if the coverage was between ≥10X but ≤30X.
‡REF = nucleotide or amino acid in Wuhan-Hu-1 reference genome.
§ALT = nucleotide or amino acid in sewage samples.
¶Frequency of the detected SNP of the sample in GISAID database as of July 8, 2020.

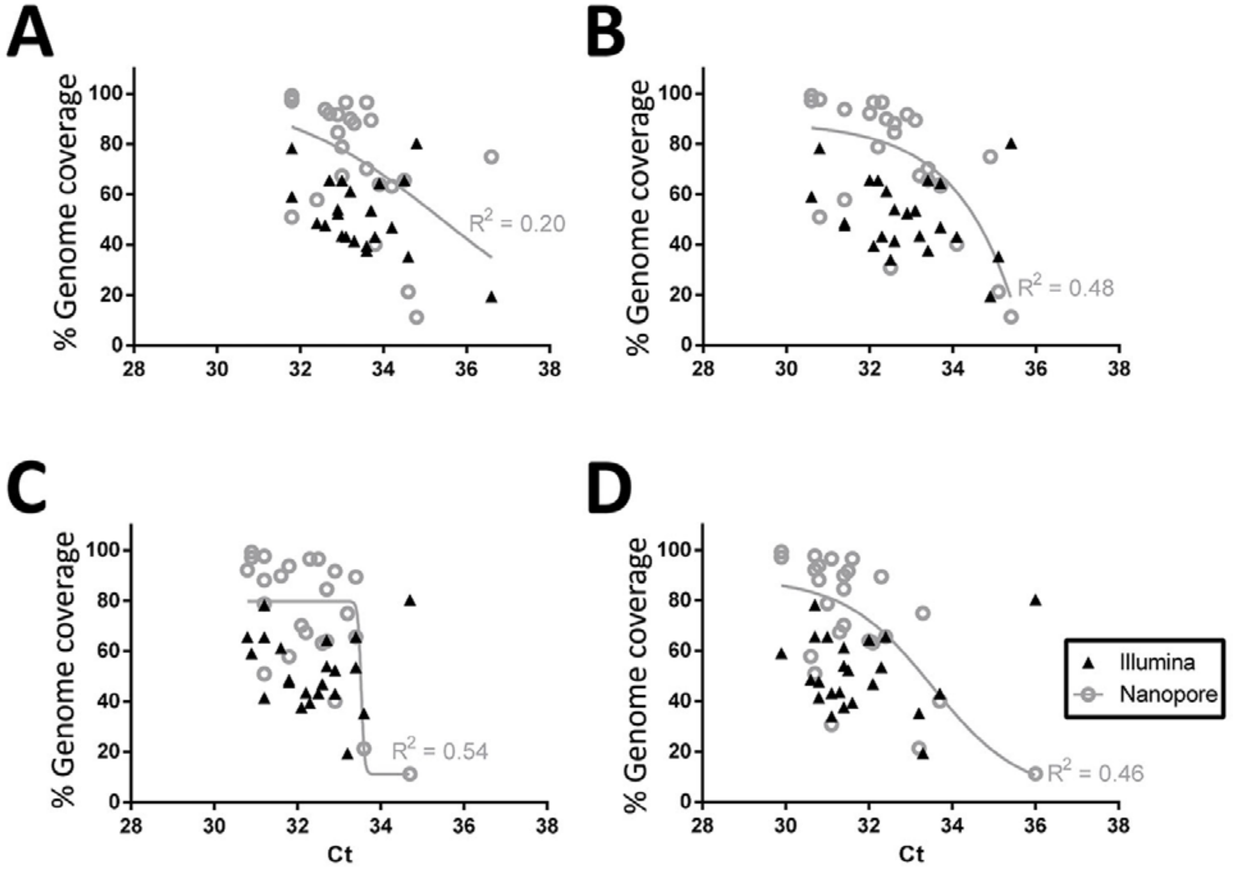

Appendix Figure 1. Reverse-transcription quantitative PCR C_t of severe acute respiratory syndrome coronavirus 2 RNA in sewage samples as determined by N gene (N1–N3) and E gene assays against the percentage of the genome covered by Illumina and nanopore reads of the same sample set, the Netherlands and Belgium. A) N1 gene. B) N2 gene. C) N3 gene. D) E gene. C_t, cycle threshold.

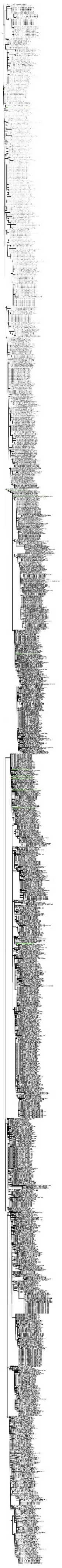

Appendix Figure 2. Fully extended phylogenetic tree of severe acute respiratory syndrome coronavirus 2 genome consensus sequences detected in sewage samples in the Netherlands and Belgium within the Netherlands–Belgium dataset.

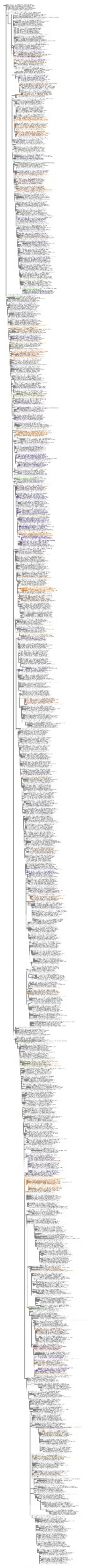

Appendix Figure 3. Fully extended phylogenetic tree of severe acute respiratory syndrome coronavirus 2 genome consensus sequences detected in sewage samples in the Netherlands and Belgium within the global subsample dataset.
